# Supplementary material for: A comprehensive, mechanistically detailed, and executable model of the cell division cycle in Saccharomyces cerevisiae
Source: Nat Commun. 2019 Mar 21;10:1308. doi: 10.1038/s41467-019-08903-w (PMC6428898; doi:10.1038/s41467-019-08903-w)
Supplement: Supplementary file 1 — Supplementay Information [file 41467_2019_8903_MOESM1_ESM.pdf]

Supplementary Information
A comprehensive, mechanistically detailed, and executable model of the Cell Division Cycle in *Saccharomyces cerevisiae*

Ulrike Münzner[1,2], Edda Klipp[1], and Marcus Krantz[1]

[1]*Theoretical Biophysics, Institute of Biology, Humboldt-Universität zu Berlin, 10115 Berlin, Germany*
[2]*Bioinformatics Center, Institute for Chemical Research, Kyoto University, Gokasho, Uji 611-0011, Japan*

Contents

1 **Supplementary Figure 1** 3

2 **Supplementary Table 1** 4

3 **Supplementary Table 2** 16

4 **Supplementary Methods** 18
- 4.1 Model architecture 18
  - 4.1.1 The molecular reaction network (MRN) 18
  - 4.1.2 The coarse-grained model (CGM) 18
  - 4.1.3 Modules 19
- 4.2 Gene expression 20
  - 4.2.1 The ECB module 22
  - 4.2.2 The Ace2/Swi5 module 24
  - 4.2.3 The SBF module 26
  - 4.2.4 The MBF module 29
  - 4.2.5 The Hcm1 module 31
  - 4.2.6 The Fkh2 module 33
  - 4.2.7 Unregulated genes module 35
- 4.3 Regulated degradation 36
  - 4.3.1 SCF-mediated degradation module 36
  - 4.3.2 Dma1-mediated degradation module 38
  - 4.3.3 APC/C-mediated degradation module 39
- 4.4 CDK assembly and activation 41
  - 4.4.1 The Pho85 module 41
  - 4.4.2 The Cdc28 module 43
  - 4.4.3 The Cdc14 module 46
- 4.5 DNA replication 48
  - 4.5.1 DNA licensing 48
  - 4.5.2 DNA Replication initiation 50
  - 4.5.3 Biology 50
  - 4.5.4 Implementation 50
  - 4.5.5 DNA replication module 52
  - 4.5.6 DNA separation module 55
- 4.6 SPB duplication and nuclear division 57
  - 4.6.1 SPB satellite formation module 57
  - 4.6.2 SPB duplication plaque formation module 59

4.6.3 SPB duplication module . . . 61
4.6.4 SPB separation module . . . 63
4.6.5 SPB spindle tension module . . . 66
4.7 Cell division . . . 70
4.7.1 Bud emergence module . . . 70
4.7.2 Bud growth module . . . 73
4.7.3 The bud morphology checkpoint module . . . 76
4.7.4 The cytokinesis module . . . 78
4.8 Additional modules . . . 81
4.8.1 The condensin module . . . 81
4.8.2 Unconnected CDK targets module . . . 82
4.8.3 Unconnected states module . . . 84
4.8.4 Test module . . . 86

Supplementary References 87

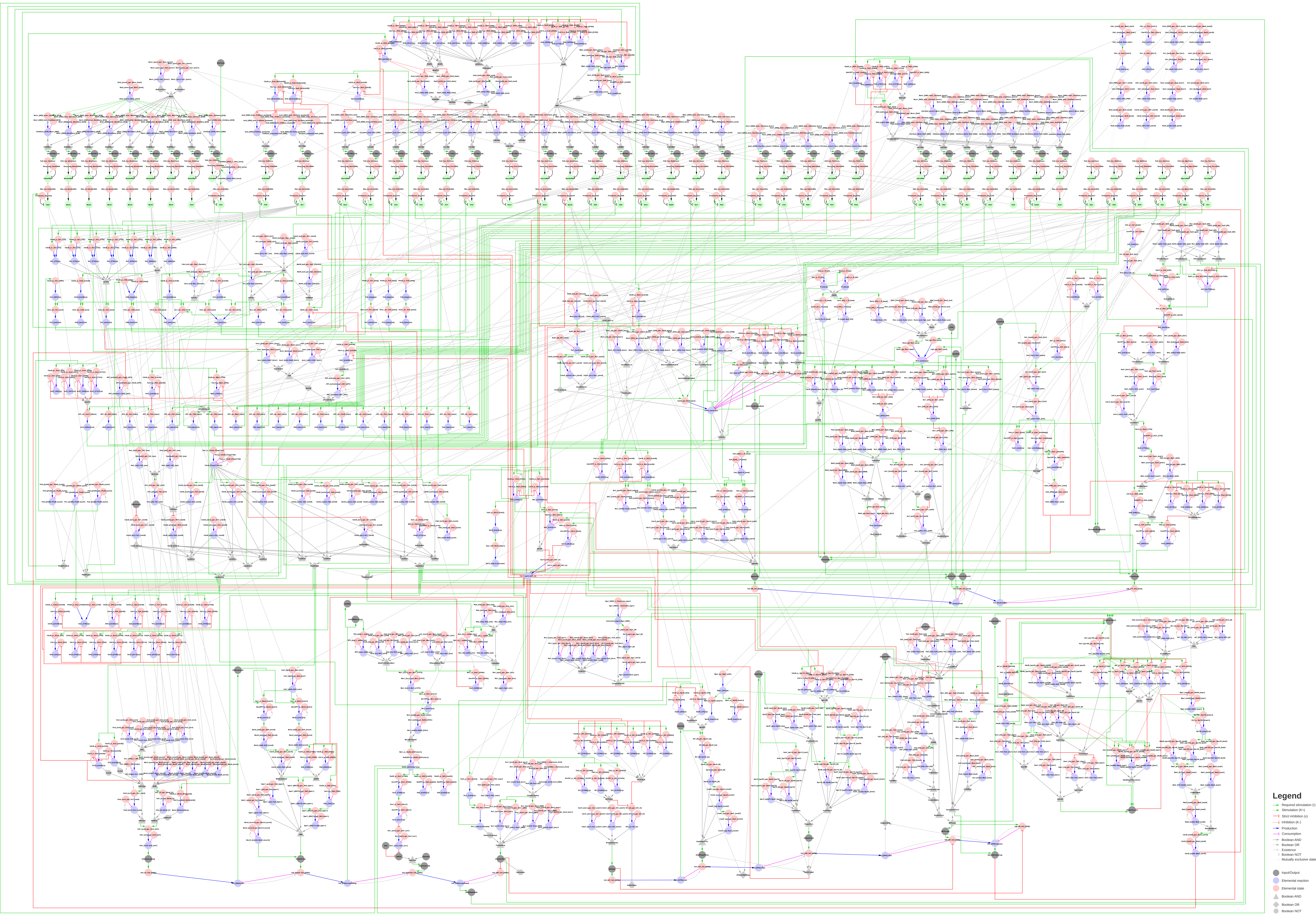

Supplementary Figure 1. The molecular architecture of the cell division cycle. Poster sized version of the network map displayed in Fig 2a. Also available at https://github.com/rxncon/models/ as CDC_S_cerevisiae_poster.pdf.

Supplementary Table 1

Model references. A list of all references used to construct the model.

[1] M. Abe, H. Qadota, A. Hirata, and Y. Ohya. Lack of gtp-bound rho1p in secretory vesicles of *Saccharomyces cerevisiae*. *The Journal of Cell Biology*, 162(1):85–97, 2003.

[2] R. Agarwal and O. Cohen-Fix. Phosphorylation of the mitotic regulator pds1/securin by cdc28 is required for efficient nuclear localization of esp1/separase. *Genes & Development*, 16(11):1371–1382, 2002.

[3] A. S. Alberts. Identification of a carboxyl-terminal diaphanous-related formin homology protein autoregulatory domain. *Journal of Biological Chemistry*, 276(4):2824–2830, 2001.

[4] A. A. Alcasabas, A. J. Osborn, J. Bachant, F. Hu, P. J. Werler, K. Bousset, K. Furuya, J. F. Diffley, A. M. Carr, and S. J. Elledge. Mrc1 transduces signals of dna replication stress to activate rad53. *Nature Cell Biology*, 3(11):958–965, 2001.

[5] G. Alexandru, F. Uhlmann, K. Mechtler, M.-A. Poupart, and K. Nasmyth. Phosphorylation of the cohesin subunit scc1 by polo/cdc5 kinase regulates sister chromatid separation in yeast. *Cell*, 105(4):459–472, 2001.

[6] V. Archambault, E. J. Chang, B. J. Drapkin, F. R. Cross, B. T. Chait, and M. P. Rout. Targeted proteomic study of the cyclin-cdk module. *Molecular Cell*, 14(6):699–711, 2004.

[7] A. Audhya and S. D. Emr. Stt4 pi 4-kinase localizes to the plasma membrane and functions in the pkc1-mediated map kinase cascade. *Developmental Cell*, 2(5):593–605, 2002.

[8] R. Azzam, S. L. Chen, W. Shou, A. S. Mah, G. Alexandru, K. Nasmyth, R. S. Annan, S. A. Carr, and R. J. Deshaies. Phosphorylation by cyclin b-cdk underlies release of mitotic exit activator cdc14 from the nucleolus. *Science*, 305(5683):516–519, 2004.

[9] N. C. Barbet, U. Schneider, S. B. Helliwell, I. Stansfield, M. F. Tuite, and M. N. Hall. Tor controls translation initiation and early g1 progression in yeast. *Molecular Biology of the Cell*, 7(1):25–42, 1996.

[10] V. I. Bashkirov, E. V. Bashkirova, E. Haghnazari, and W.-D. Heyer. Direct kinase-to-kinase signaling mediated by the fha phosphoprotein recognition domain of the dun1 dna damage checkpoint kinase. *Molecular and Cellular Biology*, 23(4):1441–1452, 2003.

[11] N. Bastajian, H. Friesen, and B. J. Andrews. Bck2 acts through the mads box protein mcm1 to activate cell-cycle-regulated genes in budding yeast. *PLoS Genetics*, 9(5):e1003507, 2013.

[12] J. M. Bean, E. D. Siggia, and F. R. Cross. High functional overlap between mlui cell-cycle box binding factor and swi4/6 cell-cycle box binding factor in the g1/s transcriptional program in *Saccharomyces cerevisiae*. *Genetics*, 171(1):49–61, 2005.

[13] S. P. Bell and K. Labib. Chromosome duplication in *Saccharomyces cerevisiae*. *Genetics*, 203(3):1027–1067, 2016.

[14] D. T. Bertazzi, B. Kurtulmus, and G. Pereira. The cortical protein lte1 promotes mitotic exit by inhibiting the spindle position checkpoint kinase kin4. *The Journal of Cell Biology*, 193(6):1033–1048, 2011.

[15] A. Bertin, M. A. McMurray, P. Grob, S.-S. Park, G. Garcia, I. Patanwala, H.-l. Ng, T. Alber, J. Thorner, and E. Nogales. *Saccharomyces cerevisiae* septins: supramolecular organization of heterooligomers and the mechanism of filament assembly. *Proceedings of the National Academy of Sciences*, 105(24):8274–8279, 2008.

[16] E. Bi and H.-O. Park. Cell polarization and cytokinesis in budding yeast. *Genetics*, 191(2):347–387, 2012.

[17] S. Biggins. The composition, functions, and regulation of the budding yeast kinetochore. *Genetics*, 194(4):817–846, 2013.

[18] S. J. Brill and S. Bastin-Shanower. Identification and characterization of the fourth single-stranded-dna binding domain of replication protein a. *Molecular and Cellular Biology*, 18(12):7225–7234, 1998.

[19] E. Bullitt, M. P. Rout, J. V. Kilmartin, and C. W. Akey. The yeast spindle pole body is assembled around a central crystal of spc42p. *Cell*, 89(7):1077–1086, 1997.

[20] A.-C. Butty, N. Perrinjaquet, A. Petit, M. Jaquenoud, J. E. Segall, K. Hofmann, C. Zwahlen, and M. Peter. A positive feedback loop stabilizes the guanine-nucleotide exchange factor cdc24 at sites of polarization. *The EMBO Journal*, 21(7):1565–1576, 2002.

[21] A. J. Caplan, D. M. Cyr, and M. G. Douglas. Ydj1p facilitates polypeptide translocation across different intracellular membranes by a conserved mechanism. *Cell*, 71(7):1143–1155, 1992.

[22] A. K. Caydasi, B. Kurtulmus, M. I. Orrico, A. Hofmann, B. Ibrahim, and G. Pereira. Elm1 kinase activates the spindle position checkpoint kinase kin4. *The Journal of Cell Biology*, 190(6):975–989, 2010.

[23] L. Y. Chan and A. Amon. The protein phosphatase 2a functions in the spindle position checkpoint by regulating the checkpoint kinase kin4. *Genes & Development*, 23(14):1639–1649, 2009.

[24] M. K. Chee and S. B. Haase. B-cyclin/cdks regulate mitotic spindle assembly by phosphorylating kinesins-5 in budding yeast. *PLoS Genetics*, 6(5):e1000935, 2010.

[25] I. M. Cheeseman, S. Anderson, M. Jwa, E. M. Green, J.-s. Kang, J. R. Yates, C. S. Chan, D. G. Drubin, and G. Barnes. Phospho-regulation of kinetochore-microtubule attachments by the aurora kinase ipl1p. *Cell*, 111(2):163–172, 2002.

[26] S. Chen, M. A. de Vries, and S. P. Bell. Orc6 is required for dynamic recruitment of cdt1 during repeated mcm2–7 loading. *Genes & Development*, 21(22):2897–2907, 2007.

[27] S.-h. Chen and H. Zhou. Reconstitution of rad53 activation by mec1 through adaptor protein mrc1. *Journal of Biological Chemistry*, 284(28):18593–18604, 2009.

[28] Y.-C. Chen, J. Kenworthy, C. Gabrielse, C. Hänni, P. Zegerman, and M. Weinreich. Dna replication checkpoint signaling depends on a rad53–dbf4 n-terminal interaction in *Saccharomyces cerevisiae*. *Genetics*, 194(2):389–401, 2013.

[29] A. Cheng, K. E. Ross, P. Kaldis, and M. J. Solomon. Dephosphorylation of cyclin-dependent kinases by type 2c protein phosphatases. *Genes & Development*, 13(22):2946–2957, 1999.

[30] O. Chilkova, P. Stenlund, I. Isoz, C. M. Stith, P. Grabowski, E.-B. Lundström, P. M. Burgers, and E. Johansson. The eukaryotic leading and lagging strand dna polymerases are loaded onto primer-ends via separate mechanisms but have comparable processivity in the presence of pcna. *Nucleic Acids Research*, 35(19):6588–6597, 2007.

[31] R. J. Cho, M. J. Campbell, E. A. Winzeler, L. Steinmetz, A. Conway, L. Wodicka, T. G. Wolfsberg, A. E. Gabrielian, D. Landsman, D. J. Lockhart, et al. A genome-wide transcriptional analysis of the mitotic cell cycle. *Molecular Cell*, 2(1):65–73, 1998.

[32] V. J. Cid, J. Jiménez, M. Molina, M. Sánchez, C. Nombela, and J. W. Thorner. Orchestrating the cell cycle in yeast: sequential localization of key mitotic regulators at the spindle pole and the bud neck. *Microbiology*, 148(9):2647–2659, 2002.

[33] R. Ciosk, W. Zachariae, C. Michaelis, A. Shevchenko, M. Mann, and K. Nasmyth. An esp1/pds1 complex regulates loss of sister chromatid cohesion at the metaphase to anaphase transition in yeast. *Cell*, 93(6):1067–1076, 1998.

[34] M. Costanzo, O. Schub, and B. Andrews. G1 transcription factors are differentially regulated in *Saccharomyces cerevisiae* by the swi6-binding protein stb1. *Molecular and Cellular Biology*, 23(14):5064–5077, 2003.

[35] M. Coué, S. L. Brenner, I. Spector, and E. D. Korn. Inhibition of actin polymerization by latrunculin a. *FEBS letters*, 213(2):316–318, 1987.

[36] L. Crabbé, A. Thomas, V. Pantesco, J. De Vos, P. Pasero, and A. Lengronne. Analysis of replication profiles reveals key role of rfc-ctf18 in yeast replication stress response. *Nature Structural & Molecular Biology*, 17(11):1391–1397, 2010.

[37] K. Crasta, P. Huang, G. Morgan, M. Winey, and U. Surana. Cdk1 regulates centrosome separation by restraining proteolysis of microtubule-associated proteins. *The EMBO Journal*, 25(11):2551–2563, 2006.

[38] D. M. Cyr and M. G. Douglas. Differential regulation of hsp70 subfamilies by the eukaryotic dnaj homologue ydj1. *Journal of Biological Chemistry*, 269(13):9798–9804, 1994.

[39] R. A. de Bruin, T. I. Kalashnikova, and C. Wittenberg. Stb1 collaborates with other regulators to modulate the g1-specific transcriptional circuit. *Molecular and Cellular Biology*, 28(22):6919–6928, 2008.

[40] J. M. Dial, E. V. Petrotchenko, and C. H. Borchers. Inhibition of apccdh1 activity by cdh1/acm1/bmh1 ternary complex formation. *Journal of Biological Chemistry*, 282(8):5237–5248, 2007.

[41] A. D. Donaldson and J. V. Kilmartin. Spc42p: a phosphorylated component of the *S. cerevisiae* spindle pole body (spb) with an essential function during spb duplication. *The Journal of Cell Biology*, 132(5):887–901, 1996.

[42] Y. Dong, D. Pruyne, and A. Bretscher. Formin-dependent actin assembly is regulated by distinct modes of rho signaling in yeast. *The Journal of cell biology*, 161(6):1081–1092, 2003.

[43] M. E. Douglas and J. F. Diffley. Recruitment of mcm10 to sites of replication initiation requires direct binding to the minichromosome maintenance (mcm) complex. *Journal of Biological Chemistry*, 291(11):5879–5888, 2016.

[44] E. R. Edenberg, K. G. Mark, and D. P. Toczyski. Ndd1 turnover by scfgrr1 is inhibited by the dna damage checkpoint in *Saccharomyces cerevisiae*. *PLoS Genetics*, 11(4):e1005162, 2015.

[45] S. Elliott, M. Knop, G. Schlenstedt, and E. Schiebel. Spc29p is a component of the spc110p subcomplex and is essential for spindle pole body duplication. *Proceedings of the National Academy of Sciences*, 96(11):6205–6210, 1999.

[46] S. Elsasser, Y. Chi, P. Yang, and J. L. Campbell. Phosphorylation controls timing of cdc6p destruction: a biochemical analysis. *Molecular Biology of the Cell*, 10(10):3263–3277, 1999.

[47] M. Elserafy, M. Šarić, A. Neuner, T.-c. Lin, W. Zhang, C. Seybold, L. Sivashanmugam, and E. Schiebel. Molecular mechanisms that restrict yeast centrosome duplication to one event per cell cycle. *Current Biology*, 24(13):1456–1466, 2014.

[48] M. Enquist-Newman, M. Sullivan, and D. O. Morgan. Modulation of the mitotic regulatory network by apc-dependent destruction of the cdh1 inhibitor acm1. *Molecular Cell*, 30(4):437–446, 2008.

[49] J. M. Enserink and R. D. Kolodner. An overview of Cdk1-controlled targets and processes. *Cell Division*, 5:11, 2010.

[50] C. B. Epstein and F. R. Cross. Clb5: a novel b cyclin from budding yeast with a role in s phase. *Genes & Development*, 6(9):1695–1706, 1992.

[51] S. Erlemann, A. Neuner, L. Gombos, R. Gibeaux, C. Antony, and E. Schiebel. An extended γ-tubulin ring functions as a stable platform in microtubule nucleation. *The Journal of Cell Biology*, 197(1):59–74, 2012.

[52] M. Evangelista, K. Blundell, M. S. Longtine, C. J. Chow, N. Adames, J. R. Pringle, M. Peter, and C. Boone. Bni1p, a yeast formin linking cdc42p and the actin cytoskeleton during polarized morphogenesis. *Science*, 276(5309):118–122, 1997.

[53] J. Fernius, O. O. Nerusheva, S. Galander, F. de Lima Alves, J. Rappsilber, and A. L. Marston. Cohesin-dependent association of scc2/4 with the centromere initiates pericentromeric cohesion establishment. *Current Biology*, 23(7):599–606, 2013.

[54] L. Francisco, W. Wang, and C. Chan. Type 1 protein phosphatase acts in opposition to ipl1 protein kinase in regulating yeast chromosome segregation. *Molecular and Cellular Biology*, 14(7):4731–4740, 1994.

[55] V. Fridman, A. Gerson-Gurwitz, O. Shapira, N. Movshovich, S. Lakämper, C. F. Schmidt, and L. Gheber. Kinesin-5 kip1 is a bi-directional motor that stabilizes microtubules and tracks their plus-ends in vivo. *J Cell Sci*, 126(18):4147–4159, 2013.

[56] J. Fu, I. M. Hagan, and D. M. Glover. The centrosome and its duplication cycle. *Cold Spring Harbor Perspectives in Biology*, 7(2):a015800, 2015.

[57] L. J. García-Rodríguez, G. De Piccoli, V. Marchesi, R. C. Jones, R. D. Edmondson, and K. Labib. A conserved polϵ binding module in ctf18-rfc is required for s-phase checkpoint activation downstream of mec1. *Nucleic Acids Research*, 43(18):8830–8838, 2015.

[58] M. Geymonat, A. Spanos, G. de Bettignies, and S. G. Sedgwick. Lte1 contributes to bfa1 localization rather than stimulating nucleotide exchange by tem1. *The Journal of Cell Biology*, 187(4):497–511, 2009.

[59] M. Geymonat, A. Spanos, S. J. Smith, E. Wheatley, K. Rittinger, L. H. Johnston, and S. G. Sedgwick. Control of mitotic exit in budding yeast *In vitro* regulation of tem1 gtpase by bub2 and bfa1. *Journal of Biological Chemistry*, 277(32):28439–28445, 2002.

[60] M. Glotzer. The 3ms of central spindle assembly: microtubules, motors and maps. *Nature Reviews. Molecular Cell Biology*, 10(1):9, 2009.

[61] M.-P. Gulli and M. Peter. Temporal and spatial regulation of rho-type guanine-nucleotide exchange factors: the yeast perspective. *Genes & Development*, 15(4):365–379, 2001.

[62] W. Guo, F. Tamanoi, and P. Novick. Spatial regulation of the exocyst complex by rho1 gtpase. *Nature Cell Biology*, 3(4):353–360, 2001.

[63] S. B. Haase and C. Wittenberg. Topology and control of the cell-cycle-regulated transcriptional circuitry. *Genetics*, 196(1):65–90, 2014.

[64] J. Hadwiger, C. Wittenberg, M. Mendenhall, and S. Reed. The *Saccharomyces cerevisiae* cks1 gene, a homolog of the *Schizosaccharomyces pombe* suc1+ gene, encodes a subunit of the cdc28 protein kinase complex. *Molecular and Cellular Biology*, 9(5):2034–2041, 1989.

[65] B.-K. Han, L. M. Bogomolnaya, J. M. Totten, H. M. Blank, L. J. Dangott, and M. Polymenis. Bem1p, a scaffold signaling protein, mediates cyclin-dependent control of vacuolar homeostasis in saccharomyces cerevisiae. *Genes & Development*, 19(21):2606–2618, 2005.

[66] B. He, F. Xi, X. Zhang, J. Zhang, and W. Guo. Exo70 interacts with phospholipids and mediates the targeting of the exocyst to the plasma membrane. *The EMBO Journal*, 26(18):4053–4065, 2007.

[67] A. M. Hegnauer, N. Hustedt, K. Shimada, B. L. Pike, M. Vogel, P. Amsler, S. M. Rubin, F. Van Leeuwen, A. Guénolé, H. Van Attikum, et al. An n-terminal acidic region of sgs1 interacts with rpa70 and recruits rad53 kinase to stalled forks. *The EMBO Journal*, 31(18):3768–3783, 2012.

[68] S. Hernández-Ortega, S. Bru, N. Ricco, S. Ramírez, N. Casals, J. Jiménez, M. Isasa, B. Crosas, and J. Clotet. Defective in mitotic arrest 1 (dma1) ubiquitin ligase controls g1 cyclin degradation. *Journal of Biological Chemistry*, 288(7):4704–4714, 2013.

[69] G. P. H. Heusden, D. J. Griffiths, J. C. Ford, P. A. Schrader, A. M. Carr, H. Y. Steensma, et al. The 14-3-3 proteins encoded by the bmh1 and bmh2 genes are essential in the yeast *Saccharomyces cerevisiae* and can be replaced by a plant homologue. *The FEBS Journal*, 229(1):45–53, 1995.

[70] E. R. Hildebrandt, L. Gheber, T. Kingsbury, and M. A. Hoyt. Homotetrameric form of cin8p, a *Saccharomyces cerevisiae* kinesin-5 motor, is essential for its in vivo function. *Journal of Biological Chemistry*, 281(36):26004–26013, 2006.

[71] Z. Hilioti, Y.-S. Chung, Y. Mochizuki, C. F. Hardy, and O. Cohen-Fix. The anaphase inhibitor pds1 binds to the apc/c-associated protein cdc20 in a destruction box-dependent manner. *Current Biology*, 11(17):1347–1352, 2001.

[72] S. M. Hinshaw, V. Makrantoni, A. Kerr, A. L. Marston, and S. C. Harrison. Structural evidence for scc4-dependent localization of cohesin loading. *Elife*, 4:e06057, 2015.

[73] K. J. Holmes, D. M. Klass, E. L. Guiney, and M. S. Cyert. Whi3, an *S. cerevisiae* rna-binding protein, is a component of stress granules that regulates levels of its target mrnas. *PLoS One*, 8(12):e84060, 2013.

[74] A. S. Howell and D. J. Lew. Morphogenesis and the cell cycle. *Genetics*, 190(1):51–77, 2012.

[75] S.-C. Hsu, D. TerBush, M. Abraham, and W. Guo. The exocyst complex in polarized exocytosis. volume 233 of *International Review of Cytology*, pages 243 – 265. Academic Press, 2004.

[76] D. Huang, S. Kaluarachchi, D. van Dyk, H. Friesen, R. Sopko, W. Ye, N. Bastajian, J. Moffat, H. Sassi, M. Costanzo, et al. Dual regulation by pairs of cyclin-dependent protein kinases and histone deacetylases controls g1 transcription in budding yeast. *PLoS Biology*, 7(9):e1000188, 2009.

[77] C. Iftode, Y. Daniely, and J. A. Borowiec. Replication protein a (rpa): the eukaryotic ssb. *Critical Reviews in Biochemistry and Molecular Biology*, 34(3):141–180, 1999.

[78] J. Imai, A. Toh-e, and Y. Matsui. Genetic analysis of the saccharomyces cerevisiae rho3 gene, encoding a rho-type small gtpase, provides evidence for a role in bud formation. *Genetics*, 142(2):359–369, 1996.

[79] S. Irniger. Cyclin destruction in mitosis: a crucial task of cdc20. *FEBS letters*, 532(1-2):7–11, 2002.

[80] M. Iwase, J. Luo, S. Nagaraj, M. Longtine, H. B. Kim, B. K. Haarer, C. Caruso, Z. Tong, J. R. Pringle, and E. Bi. Role of a cdc42p effector pathway in recruitment of the yeast septins to the presumptive bud site. *Molecular Biology of the Cell*, 17(3):1110–1125, 2006.

[81] L. P. Jackson, S. I. Reed, and S. B. Haase. Distinct mechanisms control the stability of the related s-phase cyclins clb5 and clb6. *Molecular and Cellular Biology*, 26(6):2456–2466, 2006.

[82] S. L. Jaspersen, B. J. Huneycutt, T. H. Giddings, K. A. Resing, N. G. Ahn, and M. Winey. Cdc28/cdk1 regulates spindle pole body duplication through phosphorylation of spc42 and mps1. *Developmental Cell*, 7(2):263–274, 2004.

[83] Y. Jiang and J. R. Broach. Tor proteins and protein phosphatase 2a reciprocally regulate tap42 in controlling cell growth in yeast. *The EMBO Journal*, 18(10):2782–2792, 1999.

[84] Y.-L. Juang, J. Huang, J.-M. Peters, M. E. McLaughlin, C.-Y. Tai, and D. Pellman. Apc-mediated proteolysis of ase1 and the morphogenesis of the mitotic spindle. *Science*, 275(5304):1311–1314, 1997.

[85] P. Kaiser, R. A. Sia, E. G. Bardes, D. J. Lew, and S. I. Reed. Cdc34 and the f-box protein met30 are required for degradation of the cdk-inhibitory kinase swe1. *Genes & Development*, 12(16):2587–2597, 1998.

[86] J.-s. Kang, I. M. Cheeseman, G. Kallstrom, S. Velmurugan, G. Barnes, and C. S. Chan. Functional cooperation of dam1, ipl1, and the inner centromere protein (incenp)–related protein sli15 during chromosome segregation. *The Journal of Cell Biology*, 155(5):763–774, 2001.

[87] P. J. Kang, M. E. Lee, and H.-O. Park. Bud3 activates cdc42 to establish a proper growth site in budding yeast. *The Journal of Cell Biology*, 206(1):19–28, 2014.

[88] N. Kantake, T. Sugiyama, R. D. Kolodner, and S. C. Kowalczykowski. The recombination-deficient mutant rpa (rfa1-t11) is displaced slowly from single-stranded dna by rad51 protein. *Journal of Biological Chemistry*, 278(26):23410–23417, 2003.

[89] M. A. Keaton, E. S. Bardes, A. R. Marquitz, C. D. Freel, T. R. Zyla, J. Rudolph, and D. J. Lew. Differential susceptibility of yeast s and m phase cdk complexes to inhibitory tyrosine phosphorylation. *Current Biology*, 17(14):1181–1189, 2007.

[90] J. M. Keck, M. H. Jones, C. C. Wong, J. Binkley, D. Chen, S. L. Jaspersen, E. P. Holinger, T. Xu, M. Niepel, M. P. Rout, et al. A cell cycle phosphoproteome of the yeast centrosome. *Science*, 332(6037):1557–1561, 2011.

[91] S. Kemmler, M. Stach, M. Knapp, J. Ortiz, J. Pfannstiel, T. Ruppert, and J. Lechner. Mimicking ndc80 phosphorylation triggers spindle assembly checkpoint signalling. *The EMBO Journal*, 28(8):1099–1110, 2009.

[92] J. o. Kim, A. Zelter, N. T. Umbreit, A. Bollozos, M. Riffle, R. Johnson, M. J. MacCoss, C. L. Asbury, and T. N. Davis. The ndc80 complex bridges two dam1 complex rings. *eLife*, 6:e21069, 2017.

[93] C. Koch, A. Schleiffer, G. Ammerer, and K. Nasmyth. Switching transcription on and off during the yeast cell cycle: Cln/cdc28 kinases activate bound transcription factor sbf (swi4/swi6) at start, whereas clb/cdc28 kinases displace it from the promoter in g2. *Genes & Development*, 10(2):129–141, 1996.

[94] H. Kohno, K. Tanaka, A. Mino, M. Umikawa, H. Imamura, T. Fujiwara, Y. Fujita, K. Hotta, H. Qadota, T. Watanabe, et al. Bni1p implicated in cytoskeletal control is a putative target of rho1p small gtp binding protein in *Saccharomyces cerevisiae*. *The EMBO Journal*, 15(22):6060, 1996.

[95] M. Kõivomägi, E. Valk, R. Venta, A. Iofik, M. Lepiku, D. O. Morgan, and M. Loog. Dynamics of cdk1 substrate specificity during the cell cycle. *Molecular Cell*, 42(5):610–623, 2011.

[96] S. I. Komarnitsky, Y.-C. Chiang, F. C. Luca, J. Chen, J. H. Toyn, M. Winey, L. H. Johnston, and C. L. Denis. Dbf2 protein kinase binds to and acts through the cell cycle-regulated mob1 protein. *Molecular and Cellular Biology*, 18(4):2100–2107, 1998.

[97] K. Kono, S. Nogami, M. Abe, M. Nishizawa, S. Morishita, D. Pellman, and Y. Ohya. G1/s cyclin-dependent kinase regulates small gtpase rho1p through phosphorylation of rhogef tus1p in *Saccharomyces cerevisiae*. *Molecular Biology of the Cell*, 19(4):1763–1771, 2008.

[98] R. Koren, L. Rainis, and T. Kleinberger. The scaffolding a/tpd3 subunit and high phosphatase activity are dispensable for cdc55 function in the *Saccharomyces cerevisiae* spindle checkpoint and in cytokinesis. *Journal of Biological Chemistry*, 279(47):48598–48606, 2004.

[99] F. Lampert, P. Hornung, and S. Westermann. The dam1 complex confers microtubule plus end–tracking activity to the ndc80 kinetochore complex. *The Journal of Cell Biology*, 189(4):641–649, 2010.

[100] B. D. Landry, J. P. Doyle, D. P. Toczyski, and J. A. Benanti. F-box protein specificity for g1 cyclins is dictated by subcellular localization. *PLoS Genetics*, 8(7):e1002851, 2012.

[101] K. S. Lee, S. Asano, J.-E. Park, K. Sakchaisri, and R. L. Erikson. Monitoring the cell cycle by multi-kinase-dependent regulation of swe1/wee1 in budding yeast. *Cell Cycle*, 4(10):1346–1349, 2005.

[102] T. Legal, J. Zou, A. Sochaj, J. Rappsilber, and J. P. Welburn. Molecular architecture of the dam1 complex–microtubule interaction. *Open Biology*, 6(3):150237, 2016.

[103] M. E. Liku, V. Q. Nguyen, A. W. Rosales, K. Irie, and J. J. Li. Cdk phosphorylation of a novel nls-nes module distributed between two subunits of the mcm2-7 complex prevents chromosomal rereplication. *Molecular Biology of the Cell*, 16(10):5026–5039, 2005.

[104] T.-c. Lin, A. Neuner, Y. T. Schlosser, E. Schiebel, A. N. Scharf, and L. Weber. Cell-cycle dependent phosphorylation of yeast pericentrin regulates γ-tusc-mediated microtubule nucleation. *Elife*, 3:e02208, 2014.

[105] C. Linke, A. Chasapi, A. González-Novo, I. Al Sawad, S. Tognetti, E. Klipp, M. Loog, S. Krobitsch, F. Posas, I. Xenarios, et al. A clb/cdk1-mediated regulation of fkh2 synchronizes clb expression in the budding yeast cell cycle. *npj Systems Biology and Applications*, 3:1, 2017.

[106] B. Liu, L. Larsson, A. Caballero, X. Hao, D. Öling, J. Grantham, and T. Nyström. The polarisome is required for segregation and retrograde transport of protein aggregates. *Cell*, 140(2):257–267, 2010.

[107] D. Liu, G. Vader, M. J. Vromans, M. A. Lampson, and S. M. Lens. Sensing chromosome bi-orientation by spatial separation of aurora b kinase from kinetochore substrates. *Science*, 323(5919):1350–1353, 2009.

[108] M. Loog and D. O. Morgan. Cyclin specificity in the phosphorylation of cyclin-dependent kinase substrates. *Nature*, 434(7029):104–108, 2005.

[109] M. Lõoke, M. F. Maloney, and S. P. Bell. Mcm10 regulates dna replication elongation by stimulating the cmg replicative helicase. *Genes & Development*, 31(3):291–305, 2017.

[110] J. Lopez-Mosqueda, N. L. Maas, Z. O. Jonsson, L. G. D. Eli, J. Wohlschlegel, and D. P. Toczyski. Damage-induced phosphorylation of sld3 is important to block late origin firing. *Nature*, 467(7314):479, 2010.

[111] H. Lou, M. Komata, Y. Katou, Z. Guan, C. C. Reis, M. Budd, K. Shirahige, and J. L. Campbell. Mrc1 and dna polymerase ϵ function together in linking dna replication and the s phase checkpoint. *Molecular Cell*, 32(1):106–117, 2008.

[112] D. Lu, J. Y. Hsiao, N. E. Davey, V. A. Van Voorhis, S. A. Foster, C. Tang, and D. O. Morgan. Multiple mechanisms determine the order of apc/c substrate degradation in mitosis. *The Journal of Cell Biology*, 207(1):23–39, 2014.

[113] A. S. Lyon, G. Morin, M. Moritz, K. C. B. Yabut, T. Vojnar, A. Zelter, E. Muller, T. N. Davis, and D. A. Agard. Higher-order oligomerization of spc110p drives γ-tubulin ring complex assembly. *Molecular Biology of the Cell*, 27(14):2245–2258, 2016.

[114] K. D. MacIsaac, T. Wang, D. B. Gordon, D. K. Gifford, G. D. Stormo, and E. Fraenkel. An improved map of conserved regulatory sites for *Saccharomyces cerevisiae*. *BMC Bioinformatics*, 7(1):113, 2006.

[115] H. Maekawa, C. Priest, J. Lechner, G. Pereira, and E. Schiebel. The yeast centrosome translates the positional information of the anaphase spindle into a cell cycle signal. *The Journal of Cell Biology*, 179(3):423–436, 2007.

[116] A. S. Mah, J. Jang, and R. J. Deshaies. Protein kinase cdc15 activates the dbf2-mob1 kinase complex. *Proceedings of the National Academy of Sciences*, 98(13):7325–7330, 2001.

[117] M. E. Malo, S. D. Postnikoff, T. G. Arnason, and T. A. Harkness. Mitotic degradation of yeast fkh1 by the anaphase promoting complex is required for normal longevity, genomic stability and stress resistance. *Aging (Albany NY)*, 8(4):810, 2016.

[118] S. M. Markus, K. A. Kalutkiewicz, and W.-L. Lee. Astral microtubule asymmetry provides directional cues for spindle positioning in budding yeast. *Experimental Cell Research*, 318(12):1400–1406, 2012.

[119] A. L. Marston. Chromosome segregation in budding yeast: sister chromatid cohesion and related mechanisms. *Genetics*, 196(1):31–63, 2014.

[120] H. Masumoto, A. Sugino, and H. Araki. Dpb11 controls the association between dna polymerases α and ϵ and the autonomously replicating sequence region of budding yeast. *Molecular and Cellular Biology*, 20(8):2809–2817, 2000.

[121] D. McCusker, C. Denison, S. Anderson, T. A. Egelhofer, J. R. Yates, S. P. Gygi, and D. R. Kellogg. Cdk1 coordinates cell-surface growth with the cell cycle. *Nature Cell Biology*, 9(5):506–515, 2007.

[122] V. Measday, L. Moore, R. Retnakaran, J. Lee, M. Donoviel, A. Neiman, and B. Andrews. A family of cyclin-like proteins that interact with the pho85 cyclin-dependent kinase. *Molecular and Cellular Biology*, 17(3):1212–1223, 1997.

[123] M. D. Mendenhall and A. E. Hodge. Regulation of cdc28 cyclin-dependent protein kinase activity during the cell cycle of the yeast *Saccharomyces cerevisiae*. *Microbiology and Molecular Biology Reviews*, 62(4):1191–1243, 1998.

[124] J. L. Miranda, D. S. King, and S. C. Harrison. Protein arms in the kinetochore-microtubule interface of the yeast dash complex. *Molecular Biology of the Cell*, 18(7):2503–2510, 2007.

[125] E. V. Mirkin and S. M. Mirkin. Replication fork stalling at natural impediments. *Microbiology and Molecular Biology Reviews*, 71(1):13–35, 2007.

[126] D. A. Mohl, M. J. Huddleston, T. S. Collingwood, R. S. Annan, and R. J. Deshaies. Dbf2–mob1 drives relocalization of protein phosphatase cdc14 to the cytoplasm during exit from mitosis. *The Journal of Cell Biology*, 184(4):527–539, 2009.

[127] T. Moll, G. Tebb, U. Surana, H. Robitsch, and K. Nasmyth. The role of phosphorylation and the cdc28 protein kinase in cell cycle-regulated nuclear import of the *S. cerevisiae* transcription factor sw15. *Cell*, 66(4):743–758, 1991.

[128] J. K. Moore and R. K. Miller. The cyclin-dependent kinase cdc28p regulates multiple aspects of kar9p function in yeast. *Molecular Biology of the Cell*, 18(4):1187–1202, 2007.

[129] E. M. Mortensen, W. Haas, M. Gygi, S. P. Gygi, and D. R. Kellogg. Cdc28-dependent regulation of the cdc5/polo kinase. *Current Biology*, 15(22):2033–2037, 2005.

[130] J. B. Moseley and B. L. Goode. The yeast actin cytoskeleton: from cellular function to biochemical mechanism. *Microbiology and Molecular Biology Reviews*, 70(3):605–645, 2006.

[131] P. Müller, S. Park, E. Shor, D. J. Huebert, C. L. Warren, A. Z. Ansari, M. Weinreich, M. L. Eaton, D. M. MacAlpine, and C. A. Fox. The conserved bromo-adjacent homology domain of yeast orc1 functions in the selection of dna replication origins within chromatin. *Genes & Development*, 24(13):1418–1433, 2010.

[132] Y. Nakajima, R. G. Tyers, C. C. Wong, J. R. Yates, D. G. Drubin, and G. Barnes. Nbl1p: a borealin/dasra/csc-1-like protein essential for aurora/ipl1 complex function and integrity in *Saccharomyces cerevisiae*. *Molecular Biology of the Cell*, 20(6):1772–1784, 2009.

[133] P. Nash, X. Tang, S. Orlicky, Q. Chen, F. B. Gertler, M. D. Mendenhall, F. Sicheri, T. Pawson, and M. Tyers. Multisite phosphorylation of a cdk inhibitor sets a threshold for the onset of dna replication. *Nature*, 414(6863):514–521, 2001.

[134] K. Nasmyth and L. Dirick. The role of swi4 and swi6 in the activity of g1 cyclins in yeast. *Cell*, 66(5):995–1013, 1991.

[135] E. Nazarova, E. O'Toole, S. Kaitna, P. Francois, M. Winey, and J. Vogel. Distinct roles for antiparallel microtubule pairing and overlap during early spindle assembly. *Molecular Biology of the Cell*, 24(20):3238–3250, 2013.

[136] B. Nelson, C. Kurischko, J. Horecka, M. Mody, P. Nair, L. Pratt, A. Zougman, L. D. McBroom, T. R. Hughes, C. Boone, et al. Ram: a conserved signaling network that regulates ace2p transcriptional activity and polarized morphogenesis. *Molecular Biology of the Cell*, 14(9):3782–3803, 2003.

[137] V. Q. Nguyen, J. J. Li, et al. Cyclin-dependent kinases prevent dna re-replication through multiple mechanisms. *Nature*, 411(6841):1068–1073, 2001.

[138] M. Nishizawa, M. Kawasumi, M. Fujino, and A. Toh-e. Phosphorylation of sic1, a cyclin-dependent kinase (cdk) inhibitor, by cdk including pho85 kinase is required for its prompt degradation. *Molecular Biology of the Cell*, 9(9):2393–2405, 1998.

[139] E. Nogales, M. Whittaker, R. A. Milligan, and K. H. Downing. High-resolution model of the microtubule. *Cell*, 96(1):79–88, 1999.

[140] Y. Oh, K.-J. Chang, P. Orlean, C. Wloka, R. Deshaies, and E. Bi. Mitotic exit kinase dbf2 directly phosphorylates chitin synthase chs2 to regulate cytokinesis in budding yeast. *Molecular Biology of the Cell*, 23(13):2445–2456, 2012.

[141] D. Ostapenko, J. L. Burton, and M. J. Solomon. Identification of anaphase promoting complex substrates in *S. cerevisiae*. *PLoS One*, 7(9):e45895, 2012.

[142] D. Ostapenko, J. L. Burton, R. Wang, and M. J. Solomon. Pseudosubstrate inhibition of the anaphase-promoting complex by acm1: regulation by proteolysis and cdc28 phosphorylation. *Molecular and Cellular Biology*, 28(15):4653–4664, 2008.

[143] D. Ostapenko and M. J. Solomon. Anaphase promoting complex–dependent degradation of transcriptional repressors nrm1 and yhp1 in *Saccharomyces cerevisiae*. *Molecular Biology of the Cell*, 22(13):2175–2184, 2011.

[144] G. Palou, R. Palou, F. Zeng, A. A. Vashisht, J. A. Wohlschlegel, and D. G. Quintana. Three different pathways prevent chromosome segregation in the presence of dna damage or replication stress in budding yeast. *PLoS Genetics*, 11(9):e1005468, 2015.

[145] P. Palumbo, M. Vanoni, V. Cusimano, S. Busti, F. Marano, C. Manes, and L. Alberghina. Whi5 phosphorylation embedded in the g1/s network dynamically controls critical cell size and cell fate. *Nature Communications*, 7, 2016.

[146] B. Pardo, L. Crabbé, and P. Pasero. Signaling pathways of replication stress in yeast. *FEMS Yeast Research*, 17(2), 2017.

[147] C. J. Park, J.-E. Park, T. S. Karpova, N.-K. Soung, L.-R. Yu, S. Song, K. H. Lee, X. Xia, E. Kang, I. Dabanoglu, et al. Requirement for the budding yeast polo kinase cdc5 in proper microtubule growth and dynamics. *Eukaryotic Cell*, 7(3):444–453, 2008.

[148] E. E. Patton, A. R. Willems, D. Sa, L. Kuras, D. Thomas, K. L. Craig, and M. Tyers. Cdc53 is a scaffold protein for multiple cdc34/skp1/f-box protein complexes that regulate cell division and methionine biosynthesis in yeast. *Genes & Development*, 12(5):692–705, 1998.

[149] K. Peplowska, A. U. Wallek, and Z. Storchova. Sgo1 regulates both condensin and ipl1/aurora b to promote chromosome biorientation. *PLoS Genetics*, 10(6):e1004411, 2014.

[150] A. M. Perez, G. C. Finnigan, F. M. Roelants, and J. Thorner. Septin-associated protein kinases in the yeast *Saccharomyces cerevisiae*. *Frontiers in Cell and Developmental Biology*, 4, 2016.

[151] G. Perkins, L. S. Drury, and J. F. Diffley. Separate scf cdc4 recognition elements target cdc6 for proteolysis in s phase and mitosis. *The EMBO Journal*, 20(17):4836–4845, 2001.

[152] M. Peter and I. Herskowitz. Direct inhibition of the yeast cyclin-dependent kinase cdc28-cln by far1. *Science*, 265(5176):1228–1232, 1994.

[153] B. Philip and D. E. Levin. Wsc1 and mid2 are cell surface sensors for cell wall integrity signaling that act through rom2, a guanine nucleotide exchange factor for rho1. *Molecular and Cellular Biology*, 21(1):271–280, 2001.

[154] D. Philipova, J. R. Mullen, H. S. Maniar, J. Lu, C. Gu, and S. J. Brill. A hierarchy of ssb protomers in replication protein a. *Genes & Development*, 10(17):2222–2233, 1996.

[155] A. Pic-Taylor, Z. Darieva, B. A. Morgan, and A. D. Sharrocks. Regulation of cell cycle-specific gene expression through cyclin-dependent kinase-mediated phosphorylation of the forkhead transcription factor fkh2p. *Molecular and Cellular Biology*, 24(22):10036–10046, 2004.

[156] O. Poch, E. Schwob, F. de Fraipont, A. Camasses, R. Bordonné, and R. P. Martin. Rpk1, an essential yeast protein kinase involved in the regulation of the onset of mitosis, shows homology to mammalian dual-specificity kinases. *Molecular and General Genetics MGG*, 243(6):641–653, 1994.

[157] T. Pramila, S. Miles, D. GuhaThakurta, D. Jemiolo, and L. L. Breeden. Conserved homeodomain proteins interact with mads box protein mcm1 to restrict ecb-dependent transcription to the m/g1 phase of the cell cycle. *Genes & Development*, 16(23):3034–3045, 2002.

[158] T. Pramila, W. Wu, S. Miles, W. S. Noble, and L. L. Breeden. The forkhead transcription factor hcm1 regulates chromosome segregation genes and fills the s-phase gap in the transcriptional circuitry of the cell cycle. *Genes & Development*, 20(16):2266–2278, 2006.

[159] D. Pruyne, M. Evangelista, C. Yang, E. Bi, S. Zigmond, A. Bretscher, and C. Boone. Role of formins in actin assembly: nucleation and barbed-end association. *Science*, 297(5581):612–615, 2002.

[160] D. Pruyne, L. Gao, E. Bi, and A. Bretscher. Stable and dynamic axes of polarity use distinct formin isoforms in budding yeast. *Molecular Biology of the Cell*, 15(11):4971–4989, 2004.

[161] H. Qadota, C. P. Python, S. B. Inoue, M. Arisawa, Y. Anraku, Y. Zheng, T. Watanabe, D. E. Levin, and Y. Ohya. Identification of yeast rho1p gtpase as a regulatory subunit of 1, 3-β-glucan synthase. *Science*, pages 279–281, 1996.

[162] E. Queralt, C. Lehane, B. Novak, and F. Uhlmann. Downregulation of pp2a cdc55 phosphatase by separase initiates mitotic exit in budding yeast. *Cell*, 125(4):719–732, 2006.

[163] I. Quilis and J. C. Igual. A comparative study of the degradation of yeast cyclins cln1 and cln2. *FEBS open bio*, 7(1):74–87, 2017.

[164] C. K. Rane and A. Minden. P21 activated kinases: structure, regulation, and functions. *Small GTPases*, 5(1):e28003, 2014.

[165] G. J. Reynard, W. Reynolds, R. Verma, and R. J. Deshaies. Cks1 is required for g1cyclin–cyclin-dependent kinase activity in budding yeast. *Molecular and Cellular Biology*, 20(16):5858–5864, 2000.

[166] H. E. Richardson, C. Wittenberg, F. Cross, and S. I. Reed. An essential g1 function for cyclin-like proteins in yeast. *Cell*, 59(6):1127–1133, 1989.

[167] T. J. Richman, K. A. Toenjes, S. E. Morales, K. C. Cole, B. T. Wasserman, C. M. Taylor, J. A. Koster, M. F. Whelihan, and D. I. Johnson. Analysis of cell-cycle specific localization of the rdi1p rhogdi and the structural determinants required for cdc42p membrane localization and clustering at sites of polarized growth. *Current Genetics*, 45(6):339–349, Jun 2004.

[168] C. G. Riedel, V. L. Katis, Y. Katou, S. Mori, T. Itoh, W. Helmhart, M. Gálová, M. Petronczki, J. Gregan, B. Cetin, et al. Protein phosphatase 2a protects centromeric sister chromatid cohesion during meiosis i. *Nature*, 441(7089):53–61, 2006.

[169] H.-S. Ro, S. Song, and K. S. Lee. Bfa1 can regulate tem1 function independently of bub2 in the mitotic exit network of *Saccharomyces cerevisiae*. *Proceedings of the National Academy of Sciences*, 99(8):5436–5441, 2002.

[170] X. Robellet, Y. Thattikota, F. Wang, T.-L. Wee, M. Pascariu, S. Shankar, É. Bonneil, C. M. Brown, and D. D'Amours. A high-sensitivity phospho-switch triggered by cdk1 governs chromosome morphogenesis during cell division. *Genes & Development*, 29(4):426–439, 2015.

[171] J. M. Rock, D. Lim, L. Stach, R. W. Ogrodowicz, J. M. Keck, M. H. Jones, C. C. Wong, J. R. Yates, M. Winey, S. J. Smerdon, et al. Activation of the yeast hippo pathway by phosphorylation-dependent assembly of signaling complexes. *Science*, 340(6134):871–875, 2013.

[172] J.-A. Rodriguez-Rodriguez, Y. Moyano, S. Játiva, and E. Queralt. Mitotic exit function of polo-like kinase cdc5 is dependent on sequential activation by cdk1. *Cell Reports*, 15(9):2050–2062, 2016.

[173] J. Roostalu, C. Hentrich, P. Bieling, I. A. Telley, E. Schiebel, and T. Surrey. Directional switching of the kinesin cin8 through motor coupling. *Science*, 332(6025):94–99, 2011.

[174] J. S. Rosenberg, F. R. Cross, and H. Funabiki. Knl1/spc105 recruits pp1 to silence the spindle assembly checkpoint. *Current Biology*, 21(11):942–947, 2011.

[175] K. E. Ross, P. Kaldis, and M. J. Solomon. Activating phosphorylation of the *Saccharomyces cerevisiae* cyclin-dependent kinase, cdc28p, precedes cyclin binding. *Molecular Biology of the Cell*, 11(5):1597–1609, 2000.

[176] J. Rouse and S. P. Jackson. Lcd1p recruits mec1p to dna lesions in vitro and in vivo. *Molecular Cell*, 9(4):857–869, 2002.

[177] D. Rüthnick and E. Schiebel. Duplication of the yeast spindle pole body once per cell cycle. *Molecular and Cellular Biology*, 36(9):1324–1331, 2016.

[178] M. Sbia, E. J. Parnell, Y. Yu, A. E. Olsen, K. L. Kretschmann, W. P. Voth, and D. J. Stillman. Regulation of the yeast ace2 transcription factor during the cell cycle. *Journal of Biological Chemistry*, 283(17):11135–11145, 2008.

[179] J. M. Scholey, G. Civelekoglu-Scholey, and I. Brust-Mascher. Anaphase b. *Biology*, 5(4):51, 2016.

[180] S. C. Schuyler, J. Y. Liu, and D. Pellman. The molecular function of ase1p. *The Journal of Cell Biology*, 160(4):517–528, 2003.

[181] E. Schwob and K. Nasmyth. Clb5 and clb6, a new pair of b cyclins involved in s phase and mitotic spindle formation in *S. cerevisiae*. *Genes & Development*, 7:1160–1175, 1993.

[182] J. H. Seol, R. R. Feldman, W. Zachariae, A. Shevchenko, C. C. Correll, S. Lyapina, Y. Chi, M. Galova, J. Claypool, S. Sandmeyer, et al. Cdc53/cullin and the essential hrt1 ring–h2 subunit of scf define a ubiquitin ligase module that activates the e2 enzyme cdc34. *Genes & Development*, 13(12):1614–1626, 1999.

[183] W. Shou, R. Azzam, S. L. Chen, M. J. Huddleston, C. Baskerville, H. Charbonneau, R. S. Annan, S. A. Carr, and R. J. Deshaies. Cdc5 influences phosphorylation of net1 and disassembly of the rent complex. *BMC Molecular Biology*, 3(1):3, 2002.

[184] W. Shou, J. H. Seol, A. Shevchenko, C. Baskerville, D. Moazed, Z. S. Chen, J. Jang, A. Shevchenko, H. Charbonneau, and R. J. Deshaies. Exit from mitosis is triggered by tem1-dependent release of the protein phosphatase cdc14 from nucleolar rent complex. *Cell*, 97(2):233–244, 1999.

[185] Y. Shu, H. Yang, E. Hallberg, and R. Hallberg. Molecular genetic analysis of rts1p, a b′regulatory subunit of *Saccharomyces cerevisiae* protein phosphatase 2a. *Molecular and Cellular Biology*, 17(6):3242–3253, 1997.

[186] R. Sia, H. A. Herald, and D. J. Lew. Cdc28 tyrosine phosphorylation and the morphogenesis checkpoint in budding yeast. *Molecular Biology of the Cell*, 7(11):1657–1666, 1996.

[187] R. F. Siegmund and K. A. Nasmyth. The *Saccharomyces cerevisiae* start-specific transcription factor swi4 interacts through the ankyrin repeats with the mitotic clb2/cdc28 kinase and through its conserved carboxy terminus with swi6. *Molecular and Cellular Biology*, 16(6):2647–2655, 1996.

[188] D. Skowyra, K. L. Craig, M. Tyers, S. J. Elledge, and J. W. Harper. F-box proteins are receptors that recruit phosphorylated substrates to the scf ubiquitin-ligase complex. *Cell*, 91(2):209–219, 1997.

[189] S. E. Smith, B. Rubinstein, I. Mendes Pinto, B. D. Slaughter, J. R. Unruh, and R. Li. Independence of symmetry breaking on bem1-mediated autocatalytic activation of cdc42. *The Journal of Cell Biology*, 202(7):1091–1106, 2013.

[190] S. Song and K. S. Lee. A novel function of *Saccharomyces cerevisiae* cdc5 in cytokinesis. *The Journal of Cell Biology*, 152(3):451–470, 2001.

[191] R. Sopko, D. Huang, J. C. Smith, D. Figeys, and B. J. Andrews. Activation of the cdc42p gtpase by cyclin-dependent protein kinases in budding yeast. *The EMBO Journal*, 26(21):4487–4500, 2007.

[192] C. Speck, Z. Chen, H. Li, and B. Stillman. Atpase-dependent, cooperative binding of orc and cdc6p to origin dna. *Nature Structural & Molecular Biology*, 12(11):965, 2005.

[193] P. T. Spellman, G. Sherlock, M. Q. Zhang, V. R. Iyer, K. Anders, M. B. Eisen, P. O. Brown, D. Botstein, and B. Futcher. Comprehensive identification of cell cycle–regulated genes of the yeast *Saccharomyces cerevisiae* by microarray hybridization. *Molecular Biology of the Cell*, 9(12):3273–3297, 1998.

[194] M. Sullivan, C. Lehane, and F. Uhlmann. Orchestrating anaphase and mitotic exit: separase cleavage and localization of slk19. *Nature Cell Biology*, 3(9):771–777, 2001.

[195] J. Sun, H. Kawakami, J. Zech, C. Speck, B. Stillman, and H. Li. Cdc6-induced conformational changes in orc bound to origin dna revealed by cryo-electron microscopy. *Structure*, 20(3):534–544, 2012.

[196] U. Surana, H. Robitsch, C. Price, T. Schuster, I. Fitch, A. B. Futcher, and K. Nasmyth. The role of cdc28 and cyclins during mitosis in the budding yeast *S. cerevisiae*. *Cell*, 65(1):145–161, 1991.

[197] S. Tanaka, Y. Komeda, T. Umemori, Y. Kubota, H. Takisawa, and H. Araki. Efficient initiation of dna replication in eukaryotes requires dpb11/topbp1-gins interaction. *Molecular and Cellular Biology*, 33(13):2614–2622, 2013.

[198] E. M. Teh, C. C. Chai, and F. M. Yeong. Retention of chs2p in the er requires n-terminal cdk1-phosphorylation sites. *Cell Cycle*, 8(18):2965–2976, 2009.

[199] C. N. Tennyson, J. Lee, and B. J. Andrews. A role for the pcl9-pho85 cyclin–cdk complex at the m/g1 boundary in *Saccharomyces cerevisiae*. *Molecular Microbiology*, 28(1):69–79, 1998.

[200] A. Travesa, D. Kuo, R. A. De Bruin, T. I. Kalashnikova, M. Guaderrama, K. Thai, A. Aslanian, M. B. Smolka, J. R. Yates, T. Ideker, et al. Dna replication stress differentially regulates g1/s genes via rad53-dependent inactivation of nrm1. *The EMBO Journal*, 31(7):1811–1822, 2012.

[201] F. Uhlmann, F. Lottspeich, and K. Nasmyth. Sister-chromatid separation at anaphase onset is promoted by cleavage of the cohesin subunit scc1. *Nature*, 400(6739):37, 1999.

[202] M. Valerio-Santiago and F. Monje-Casas. Tem1 localization to the spindle pole bodies is essential for mitotic exit and impairs spindle checkpoint function. *The Journal of Cell Biology*, pages jcb–201007044, 2011.

[203] E. Vergés, N. Colomina, E. Garí, C. Gallego, and M. Aldea. Cyclin cln3 is retained at the er and released by the j chaperone ydj1 in late g1 to trigger cell cycle entry. *Molecular Cell*, 26(5):649–662, 2007.

[204] M. Versele and J. Thorner. Septin collar formation in budding yeast requires gtp binding and direct phosphorylation by the pak, cla4. *The Journal of Cell Biology*, 164(5):701–715, 2004.

[205] R. Visintin, K. Craig, E. S. Hwang, S. Prinz, M. Tyers, and A. Amon. The phosphatase cdc14 triggers mitotic exit by reversal of cdk-dependent phosphorylation. *Molecular Cell*, 2(6):709–718, 1998.

[206] M. V. Wagner, M. B. Smolka, R. A. De Bruin, H. Zhou, C. Wittenberg, and S. F. Dowdy. Whi5 regulation by site specific cdk-phosphorylation in *Saccharomyces cerevisiae*. *PLoS One*, 4(1):e4300, 2009.

[207] H. Wang, E. Garí, E. Verges, C. Gallego, and M. Aldea. Recruitment of cdc28 by whi3 restricts nuclear accumulation of the g1 cyclin–cdk complex to late g1. *The EMBO Journal*, 23(1):180–190, 2004.

[208] H. Wang, D. Liu, Y. Wang, J. Qin, and S. J. Elledge. Pds1 phosphorylation in response to dna damage is essential for its dna damage checkpoint function. *Genes & Development*, 15(11):1361–1372, 2001.

[209] R. Wäsch and F. R. Cross. Apc-dependent proteolysis of the mitotic cyclin clb2 is essential for mitotic exit. *Nature*, 418(6897):556, 2002.

[210] D. Watanabe, M. Abe, and Y. Ohya. Yeast lrg1p acts as a specialized rhogap regulating 1, 3-β-glucan synthesis. *Yeast*, 18(10):943–951, 2001.

[211] E. L. Weiss. Mitotic exit and separation of mother and daughter cells. *Genetics*, 192(4):1165–1202, 2012.

[212] A. C. Wild, W. Y. Jong, M. A. Lemmon, and K. J. Blumer. The p21-activated protein kinase-related kinase cla4 is a coincidence detector of signaling by cdc42 and phosphatidylinositol 4-phosphate. *Journal of Biological Chemistry*, 279(17):17101–17110, 2004.

[213] M. Winey and K. Bloom. Mitotic spindle form and function. *Genetics*, 190(4):1197–1224, 2012.

[214] B. Woods, C.-C. Kuo, C.-F. Wu, T. R. Zyla, and D. J. Lew. Polarity establishment requires localized activation of cdc42. *The Journal of Cell Biology*, 211(1):19–26, 2015.

[215] H. Wu, C. Turner, J. Gardner, B. Temple, and P. Brennwald. The exo70 subunit of the exocyst is an effector for both cdc42 and rho3 function in polarized exocytosis. *Molecular biology of the cell*, 21(3):430–442, 2010.

[216] R. Wu, J. Wang, and C. Liang. Cdt1p, through its interaction with mcm6p, is required for the formation, nuclear accumulation and chromatin loading of the mcm complex. *J Cell Sci*, 125(1):209–219, 2012.

[217] J. Yaglom, M. Linskens, S. Sadis, D. M. Rubin, B. Futcher, and D. Finley. p34cdc28-mediated control of cln3 cyclin degradation. *Molecular and Cellular Biology*, 15(2):731–741, 1995.

[218] J. A. Yaglom, A. L. Goldberg, D. Finley, and M. Y. Sherman. The molecular chaperone ydj1 is required for the p34cdc28-dependent phosphorylation of the cyclin cln3 that signals its degradation. *Molecular and Cellular Biology*, 16(7):3679–3684, 1996.

[219] Y. Yamagishi, T. Sakuno, Y. Goto, and Y. Watanabe. Kinetochore composition and its function: lessons from yeasts. *FEMS Microbiology Reviews*, 38(2):185–200, 2014.

[220] J. T. Yeeles, T. D. Deegan, A. Janska, A. Early, and J. F. Diffley. Regulated eukaryotic dna replication origin firing with purified proteins. *Nature*, 519(7544):431–435, 2015.

[221] S. K. Yelamanchi, J. Veis, D. Anrather, H. Klug, and G. Ammerer. Genotoxic stress prevents ndd1-dependent transcriptional activation of g2/m-specific genes in *Saccharomyces cerevisiae*. *Molecular and Cellular Biology*, 34(4):711–724, 2014.

[222] H. Yin, D. Pruyne, T. C. Huffaker, and A. Bretscher. Myosin v orientates the mitotic spindle in yeast. *Nature*, 406(6799):1013, 2000.

[223] H.-J. Yoon and J. Carbon. Participation of bir1p, a member of the inhibitor of apoptosis family, in yeast chromosome segregation events. *Proceedings of the National Academy of Sciences*, 96(23):13208–13213, 1999.

[224] S. Yoshida, K. Kono, D. M. Lowery, S. Bartolini, M. B. Yaffe, Y. Ohya, and D. Pellman. Polo-like kinase cdc5 controls the local activation of rho1 to promote cytokinesis. *Science*, 313(5783):108–111, 2006.

[225] S. Yoshida and A. Toh-e. Budding yeast cdc5 phosphorylates net1 and assists cdc14 release from the nucleolus. *Biochemical and biophysical research communications*, 294(3):687–691, 2002.

[226] P. Zarzov, C. Mazzoni, and C. Mann. The slt2(mpk1) map kinase is activated during periods of polarized cell growth in yeast. *The EMBO Journal*, 15(1):83–91, 1996.

[227] X. Zhao, E. G. Muller, and R. Rothstein. A suppressor of two essential checkpoint genes identifies a novel protein that negatively affects dntp pools. *Molecular Cell*, 2(3):329–340, 1998.

[228] P. Zheng, D. Fay, J. Burton, H. Xiao, J. Pinkham, and D. Stern. Spk1 is an essential s-phase-specific gene of *Saccharomyces cerevisiae* that encodes a nuclear serine/threonine/tyrosine kinase. *Molecular and Cellular Biology*, 13(9):5829–5842, 1993.

[229] J. Zich and K. G. Hardwick. Getting down to the phosphorylated 'nuts and bolts' of spindle checkpoint signalling. *Trends in Biochemical Sciences*, 35(1):18–27, 2010.

Supplementary Table 2

List of mutants examined in this study. This table lists the 85 mutants used to test the model and indicates the phenotype (viable/lethal) as well as point of arrest and the DNA copy number and number of nuclei at arrest.

Mutant	Prediction	Arrest point (Fig 4B)	DNA	Nuclei	Reference
bub2	viable	N/A			[79]
cdc28Y19F	viable	N/A			[153]
CDC5	viable	N/A			[79]
cdc55	viable	N/A			[79]
clb1	viable	N/A			[156]
clb2	viable	N/A			[156]
clb6	viable	N/A			[146]
cln1	viable	N/A			[128]
cln1cln2	viable	N/A			[79]
cln2	viable	N/A			[128]
cln3bck2whi5	viable	N/A			[79]
cln3whi5	viable	N/A			[79]
lte1	viable	N/A			[79]
spc1102AMps1	viable	N/A			[86]
ssa1	viable	N/A			[79]
whi5	viable	N/A			[79]
whi512A	viable	N/A			[168]
whi512Acln3	viable	N/A			[168]
ydj1	viable	N/A			[79]
cdc28	inviable	1a	1	1	[51]
cdc28T169A	inviable	1a	1	1	[85]
cln1cln2cln3	inviable	1a	1	1	[79]
cln1cln2cln3cdh1	inviable	1a	1	1	[79]
cln1cln2cln3sic1	inviable	1a	1	1	[79]
cln1cln2cln3whi5	inviable	1a	1	1	[79]
cln3	inviable	1a	1	1	[79]
cln3bck2	inviable	1a	1	1	[79]
cln3bck2sic1	inviable	1a	1	1	[79]
cln3mbp1	inviable	1a	1	1	[79]
cln3swi4	inviable	1a	1	1	[79]
cln3swi6	inviable	1a	1	1	[79]
bck2swi6sic1	inviable	1b	1	1	[79]
cdc20pds1cdh1SIC1	inviable	1b	1	1	[79]
cdc20pds1clb5	inviable	1b	1	1	[79]
cdc20pds1clb5cdh1	inviable	1b	1	1	[79]
cdc20pds1clb5cdh1SIC1	inviable	1b	1	1	[79]
cdc34	inviable	1b	1	1	[165]
cdc4	inviable	1b	1	1	[48]

Continued on next page

Supplementary Table 2 – *Continued from previous page*

Mutant	Prediction	Arrest point (Fig 4B)	DNA	Nuclei	Reference
cdc53	inviable	1b	1	1	[165]
cdc7	inviable	1b	1	1	[51]
clb5	inviable	1b	1	1	[146]
clb5clb6	inviable	1b	1	1	[80]
cln1cln2swi4	inviable	1b	1	1	[79]
mbp1	inviable	1b	1	1	[79]
Sic17A	inviable	1b	1	1	[104]
Sld2A	inviable	1b	1	1	[157]
Sld32A	inviable	1b	1	1	[158]
Sld3S622A	inviable	1b	1	1	[158]
Sld3T600A	inviable	1b	1	1	[158]
spc1102ACDK	inviable	1b	1	1	[86]
swi4	inviable	1b	1	1	[79]
swi4mbp1	inviable	1b	1	1	[79]
swi4mbp1BCK2	inviable	1b	1	1	[79]
swi4swi6	inviable	1b	1	1	[79]
swi4swi6sic1	inviable	1b	1	1	[79]
swi6	inviable	1b	1	1	[79]
swi64A	inviable	1b	1	1	[168]
whi54Aswi6S4A	inviable	1b	1	1	[168]
whi512Aswi6S4A	inviable	1b	1	1	[168]
cdh1	inviable	1c	1	1	[167]
cln1cln2cdh1	inviable	1c	1	1	[79]
HU_CDC5	inviable	2	1	1	[79]
clb1clb2	inviable	3a	1	1	[156]
NOC_CDC5	inviable	3a	1	1	[79]
cdc31	inviable	3c	1	1	[51]
spc1104A	inviable	3d	1	1	[86]
cdc10	inviable	3e	1	1	[51]
cdc11	inviable	3e	1	1	[51]
cdc12	inviable	3e	1	1	[51]
cdc24	inviable	3e	1	1	[51]
cdc3	inviable	3e	1	1	[51]
cdc42	inviable	3e	1	1	[68]
cdc14	inviable	4	1	1	[79]
cdc14SIC1	inviable	4	1	1	[79]
cdc20	inviable	4	1	1	[79]
cdc5	inviable	4	1	1	[79]
esp1	inviable	4			[79]
cdc20pds1	inviable	5	1	1	[79]
pds1	inviable	5	2	2	[79]
cdc6	inviable	6	1	1	[51]
net1cdc15cdh1	inviable	6	2	2	[79]
cdc15	inviable	7	*	*	[79]
cdc15net1	inviable	7	*	*	[79]
tem1	inviable	7	*	*	[79]
tem1net1	inviable	7	*	*	[79]

Supplementary Methods

Model description. Detailed description of the biology and implementation of the cell division cycle network.

4.1 Model architecture

The presented model describes the cell division cycle (CDC) of *Saccharomyces cerevisiae*, and is formulated in rxncon language. The rxncon model base consists of two parts, the molecular reaction network (MRN), and the coarse-grained model (CGM). Together, these parts form a hybrid model. The two models are connected to each other, and hence, control each other, by sharing inputs and outputs. The rxncon model base encompasses 802 reactions and 972 lines of contingencies which were translated into a bipartite Boolean network and analysed.

4.1.1 The molecular reaction network (MRN)

The MRN describes the molecular events considered in the model and is based on published empirical findings. In total, the MRN includes 357 unique components, comprising 229 proteins, and genes and mRNA of 44 proteins. The MRN consists of 790 elemental reactions producing and consuming 1235 elemental states. The elemental states are regulated by 598 contingencies. In addition, 800 lines of contingencies are used to encode time delays that are necessary for time scale separation in the bBM (Figure 2a and Supplementary Figure 1).

4.1.2 The coarse-grained model (CGM)

The CGM accounts for macroscopic characteristics of a yeast cell during a cell cycle. These macroscopic characteristics describe DNA replication, spindle pole body (SPB) duplication and nuclear division, and morphological changes of the cell (bud emergence and growth). These processes and cell states cannot be described in a feasible way on the level of molecular elemental reactions. The CGM consists of 12 macroscopic reactions and 12 macroscopic states (Figures 2b, c).

General assumptions

The presented model is based on the following general assumptions.

1. Time scale separation. The bipartite Boolean network does not take different reaction rates into account. Hence, all reaction events are executed at equal rates. This does, however, not correspond to physiological observations. Furthermore, the order of reaction execution in the Boolean network depends on the length of a path, leading to faster execution of shorter paths, even when they operate on a slower time scale *in vivo*, and delays of other processes due to longer path lengths. To compensate, we assume that transcriptional processes are slower compared to modification reactions, and force these processes to integrate over 20 times steps. We implement this through a series of intermediate steps where each depends on both the initial trigger and the previous step.

2. Dephosphorylation reactions. The model includes phosphorylation reactions which act as toggle switches. Hence, these phosphorylations must be anatagonised in order for these switches to be reset. However, such dephosphorylation reactions are sparsely described in the literature. We assume that all phosphorylated residues are dephosphorylated, and introduce hypothetical phosphatases for proteins with unknown phosphatases.

3. Degradation. We only consider turnover for a few components, where transcription, translation and/or degradation are known to be cell cycle regulated. However, for all these components, we include both synthesis and degradation. The exceptions are highly stable complexes that are subject to periodic expression. For these, we do not include (unregulated) degradation, as this would lead to their disappearance between expression peaks. Similarly, the model accounts for regulated transcription and hence, the model should reflect transcriptional deactivation. This is assumed to happen by a hypothesised decay reaction of mRNA.

4. Model rules. Boolean modelling can only account for absolute effects. Hence, we need to decide if quantitative modifiers (K+/K-) are ignored or included as absolute requirements. Here, we apply the default bipartite Boolean model (bBM) assumption and ignore these modifiers (details see [134]).

5. Spatial resolution. The current incarnation of rxncon cannot describe spatial localisation at the level of complexes. Instead, we encode the effect of localisation by directly linking the states controlling localisation to those which are regulated by localisation. Hence, we preserve the regulatory logic without explicitly including localisation. We also assume that there are no spatial effects arising from the mother-daughter asymmetry.

4.1.3 Modules

In Figure 2 of the main manuscript, we present the seven parts of the complete cell cycle network: Gene expression (Section 4.2), regulated degradation

(Section 4.3), cyclin dependent kinase (CDK) assembly and activation (Section 4.4), DNA replication (Section 4.5), SPB duplication and nuclear division (Section 4.6), cell division (Section 4.7), and additional modules with unknown regulatory connection and a test module for validation (Section 4.8). Below, we divide these network parts into 30 distinct modules, which additionally includes a module describing the implemented error functions. This modular separation is for convenience of presentation, as there are no module boundaries inside the MRN. Each module contains a functional molecular unit. Each module is presented in a figure, which also includes its neighbours (inputs, outputs) in the model.

4.2 Gene expression

The gene expression modules account for seven groups of genes/proteins that are dynamically regulated throughout the cell division cycle. The six first groups are strongly cell cycle regulated at the transcriptional level, and many of the targets are also subject to regulated degradation. The members of the seventh group are all regulated at the level of protein degradation, but we found no conclusive evidence for cell cycle regulation at the transcriptional level. We should note that their degradation has no effect on their expression during the bBM simulation, as constant expression overrules degradation, but we consider the information relevant for our knowledge base.

The model accounts for a subset of the complete number of genes with periodic expression during the CDC [154], which were selected based on their role in cell cycle control and execution (Supplementary Table 3). We only included genes encoding proteins with a known mechanistic function in cell cycle control, and where the dynamic regulation is thought to be important for this role. Most components are considered constant in the model, which would be functionally equivalent to constitutive expression.

The gene expression module accounts for synthesis of mRNA and translation into the corresponding proteins, as well as for degradation of mRNA and proteins. Transcription is realised by RNA polymerase II, abbreviated with *PolII*. Translation reactions are realised by ribosomes, abbreviated with *Ribo*. mRNA turnover is attributed to a placeholder entity *Decay* and protein degradation to the *Proteasome*.

Supplementary Table 3 *Transcriptional clusters.* The seven groups of the gene expression modules are: The early cell cycle box (ECB), Ace2/Swi5, SBF, MBF, Hcm1 and Fkh2 targets, and a group of six genes that are posttranslationally regulated. SBF: Swi4, Swi6. MBF: Swi6, Mbp1. Fkh2: Fkh2, Mcm1, Ndd1. *repressors; **regulated by SBF or MBF.

ECB	Ace2Swi5	SBF	MBF	Hcm1	Fkh2	Unregulated
CDC6	*PCL9*	*CLN1*	*ACM1*	*CIN8*	*ACE2*	*ASE1*
CLN3	*SIC1*	*CLN2*	*CLB5*	*FKH1*	*CDC20*	*CLB4*
MCM2		*HCM1***	*CLB6*	*FKH2*	*CDC5*	*DBF4*
MCM3		*PCL1*	*HCM1***	*NDD1*	*CLB1*	*FAR1*
MCM4		*PCL2*	*NRM1**	*YHP1**	*CLB2*	*KIP1*
MCM5		*SWE1*	*PDS1*		*CLB3*	*MPS1*
MCM6		*YOX1**	*RAD53*		*SWI5*	
MCM7			*SCC1*			
SWI4			*SPC42*			

4.2.1 The ECB module

Biology

The Early Cell-cycle Box (ECB) genes are regulated by the Mcm1 transcription factor and the co-repressors Yox1 and Yhp1 [121]. Bck2 has a positive influence on Mcm1 transcription [10] by binding to Mcm1. The ECB target genes include *MCM2–7* and *CDC6* [121], later used in the DNA replication process (Section 4.5), *SWI4* [121], a transcription factor regulating the SBF cluster, and *CLN3* [121], the most upstream of the cyclins that triggers the START transition in response to favourable nutrient conditions [9].

These genes are transcribed from early G_1, to support DNA licensing during a time of low CDK activity, and to integrate environmental information and to assess whether or not to commit to cell cycle progression.

The proteins Mcm2–7 and Swi4 are considered to be stable throughout the cell cycle.

Implementation

The ECB module (Supplementary Figure 2) accounts for the transcription and translation of the above mentioned nine components. Transcription by *PolII* requires prior binding of Mcm1 to the ECB element of the target gene, and is blocked if either of the co-repressors Yox1 or Yhp1 is bound to the Mcm1 protein. Translation is unregulated and only requires the presence of the mRNA, with the exception of Cln3 that additionally requires the input *[Nutrients]* to account for the role of Cln3 translation in nutrient sensing [9].

Furthermore, we consider regulated degradation of Cdc6 and Cln3, both of which are phosphorylated and ubiquitylated by the Skp, Cullin, F–box (SCF) complex [117, 81] , targeting them for degradation by the proteasome. As Mcm2-7 and Swi4 are present throughout the CDC, we do not consider their turnover.

The activating effect of Bck2 is implemented as a stimulation through binding to Mcm1.

Interfaces

The ECB module is regulated by the expression of Yox1 and Yhp1, by the ubiquitylation of Cdc6 and Cln3, and by the model input *[Nutrients]*.

The ECB effects DNA licensing through expression of Cdc6 and Mcm2-Mcm7, SBF transcription through the expression of Swi4, and Cdc28 activation through expression of Cln3.

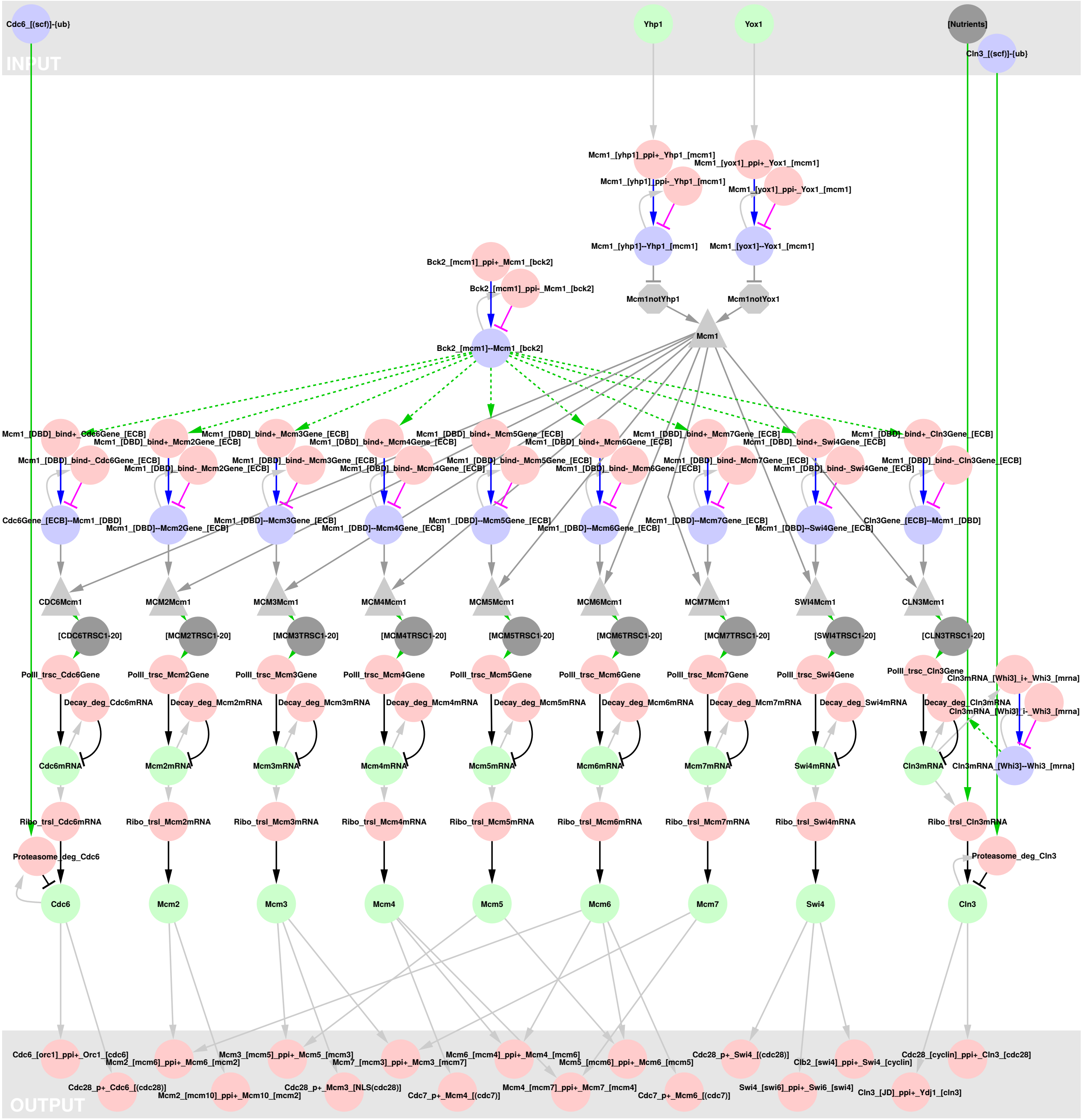

Supplementary Figure 2 *The ECB module.* The ECB module is regulated by Mcm1 in combination with the Yox1 and Yhp1 repressors, which in turn are regulated by MBF and Hcm1, respectively. The model considers nine ECB targets: *MCM2–7, CDC6, CLN3 and SWI4.* Translation of Cln3 depends on nutrient availability.

4.2.2 The Ace2/Swi5 module

Biology

Ace2 and Swi5 redundantly induce the transcription of *PCL9* [160] and *SIC1* [36]. Both transcription factors are positively regulated by Fkh2-Ndd1-Mcm1 [50]. Ace2 and Swi5 are downregulated by Cdc28-mediated phosphorylation, which results in nuclear exclusion, rendering Ace2 and Swi5 unable to bind their target promoters [141]. In addition to its nucleo-cytoplasmic regulation, Ace2 localises to the daughter nucleus, where it induces daughter-specific transcriptional programs [27]. Sic1 degradation requires both, phosphorylation at six residues [104] by Cdc28-Cln1/Cln2 [36] or Pho85 activated by Pcl1 [109], and ubiquitylation by SCF-Cdc4 [104]. Pcl9 is likely to be degraded in a similar manner to its homolog Pcl1 by ubiquitylation by Dma1 [54], which primes it for degradation by the proteasome.

Implementation

In the Ace2/Swi5 module (Supplementary Figure 3), transcription of *PCL9* and *SIC1* requires that Ace2 or Swi5 are bound to the promoter, which in turn requires that the CDK site in the nuclear localisation signal (NLS) is unphosphorylated (indicative of nuclear localisation). mRNA turnover and translation are constitutive, requiring only the presence of the mRNA, but protein degradation is regulated by ubiquitylation. Degradation for both proteins is implemented according to empirical findings: Sic1 is primed for degradation by phosphorylation by Cdc28 or Pho85, and ubiquitylation by SCF-Cdc4. Pcl9 is primed for degradation by ubiquitylation by Dma1.

Interfaces

The Ace2/Swi5 module is regulated by the expression and phosphorylation status (and hence localisation) of Ace2 and Swi5, which is controlled by Cdc28-Clb1/2 and Cdc14, as well as the ubiquitylation of Pcl9 and Sic1.

The Ace2/Swi5 module affects Pho85 activity through Pcl9 expression and Cdc28 activity through expression of the cyclin dependent kinase inhibitor (CDKI) Sic1.

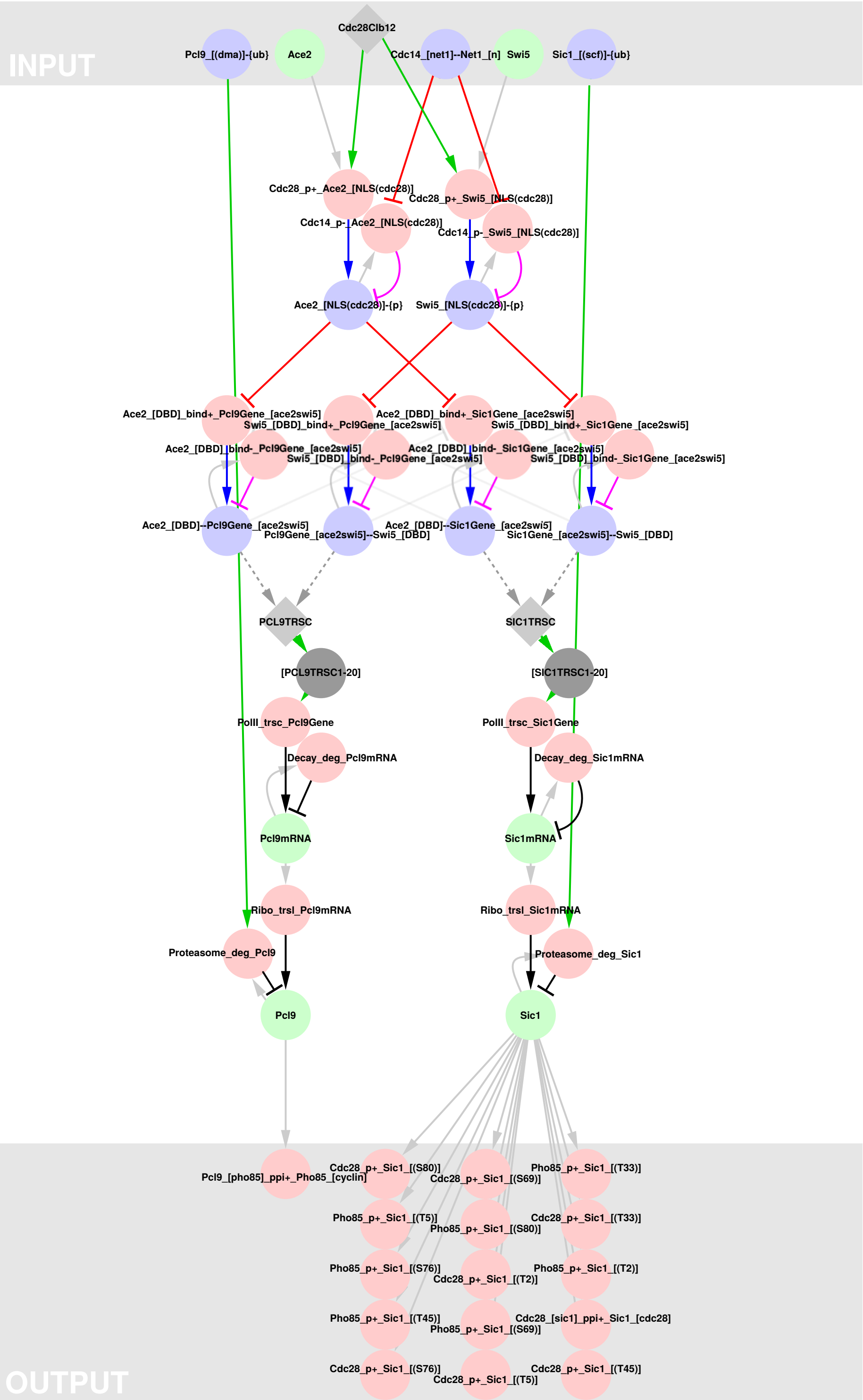

Supplementary Figure 3 *The Ace2/Swi5 module.* The transcription factors Ace2 and Swi5 redundantly regulate the genes *PCL9* and *SIC1*. Both transcription factors are spatially regulated via a nuclear localisation sequence (NLS). Cdc28-mediated phosphorylation of the NLS excludes both transcription factors from the nucleus, thereby inhibiting them from DNA binding. Cdc14 dephosphorylates Ace2 and Swi5, which enables them to enter the nucleus and bind to their target genes.

4.2.3 The SBF module

Biology

SBF is a Swi4–Swi6 dimer, which activates the transcription from Swi4/Swi6 cell cycle box (SCB) promoter elements. SBF dependent transcription is inhibited by Whi5 and the histone deacetylases (HDACs) Hos3 and Rpd3 [168]. SBF is activated through Whi5 phosphorylation by the CDK Cdc28 in complex with either of the G_1 cyclins: Cln1,Cln2, or Cln3. Hyperphosphorylated Whi5 dissociates from SCB promoters [168], and certain intermediate phosphorylation states appear to disrupt HDAC association and permit transcription [168, 62]. The SBF cluster is deactivated by Cdc28–Clb1/2-mediated phosphorylation of Swi4, which inhibits Swi4 promoter association [75].

CDK Pho85 has a partially redundant role in the activation of SBF. This phosphorylation is mediated by Pho85 interacting with either Pcl1 or Pcl9 [62]. However, the exact mechanism has not been demonstrated. It is tempting to speculate that Pho85 and Cdc28 share the same target residues in Whi5, as a Whi5 mutant with 12 CDK-site alanine substitutions in a Swi6-S4A background is not viable [168].

The SBF target genes include *SWE1, CLN1, CLN2, YOX1, PCL1* and *PCL2* [105, 25, 149, 50]. *CLN1, CLN2,* and *PCL1* are activated by SBF and contribute to Whi5 phosphorylation, constituting a positive feedback loop.

Implementation

In the SBF module (Supplementary Figure 4), SBF-mediated transcription requires the SBF heterodimer bound to the promoter, and is inhibited when the Whi5 repressor recruits the HDACs to the promoter, and through phosphorylation of Swi4 by Cdc28-Clb1/2. Hence, SBF only supports transcription if one of the Whi5 bonds to SBF and HDAC are broken. We implemented three possible mechanisms for this to happen: First, through phosphorylation of the critical sites identified by Wagner *et al.* [168] (*Whi5P1* or *Whi5P2*, combined in *Whi5P12*), which is considered to disrupt the HDAC recruitment. Second, through hyperphosphorylation of Whi5 (*Whi5Px10*), which disrupts the Swi6–Whi5 bond and excludes Whi5 from the nucleus. Third, through phosphorylation of Swi6 (*Swi6P*), which also leads to disruption of the Swi6–Whi5 bond, combined in the *Whi5release* node.

Since less is known about Pho85 regulation, the effect of its phosphorylation of Whi5 on Whi5 interactions with the HDACs was implemented as a negative influence instead of a strict inhibition.

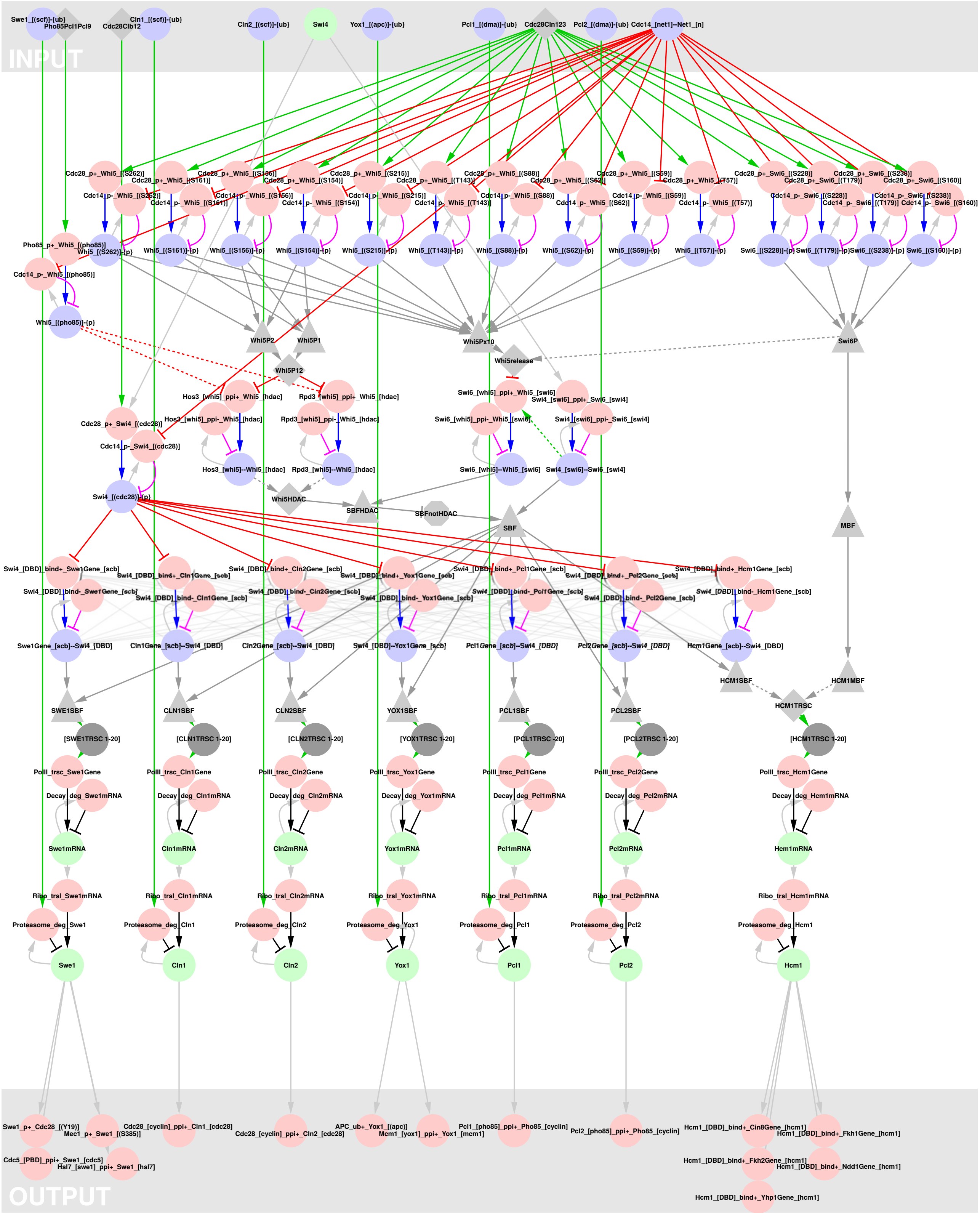

Supplementary Figure 4 *The SBF module.* SBF regulates the activation of *SWE1*, *CLN1*, *CLN2*, *YOX1*, *PCL1* and *PCL2*. SBF activity is repressed by Whi5 and activated by Cdc28–Cln1/2/3 and Pho85–Pcl1/9. Subsequent activation of Cdc28–Clb1/2 contributes to the deactivation of the module.

Interfaces

The SBF module is regulated by the expression of Swi4 and its phosphorylation by Cdc28-Clb1/2, the phosphorylation of Swi6 and Whi5 by G_1 CDK-cyclins Cdc28–Cln1/2/3 or Pho85–Pcl1/2/9, the dephosphorylation of Swi4, Swi6 and Whi5 by Cdc14, and the ubiquitylation status of Cln1, Cln2, Pcl1, Pcl2, Swe1 and Yox1.

The SBF module effects the activity of Cdc28 through expression of Cln1 and Cln2, Pho85 through expression of Pcl1 and Pcl2, ECB-mediated gene expression through the expression of the Yox1 repressor, Hcm1-mediated gene expression through expression of Hcm1, and to the morphogenesis/bud size checkpoint through expression of Swe1.

4.2.4 The MBF module

Biology

The MBF cluster is regulated by the heterodimeric MBF transcription factor, which consists of Mbp1 and Swi6. The exact mechanism of MBF activation is disputed [36], but activation of MBF regulated genes occurs slightly after SBF activation and requires binding of the Mbp1–Swi6 (MBF) dimer to Mlu1 cell cycle box (MCB) promoter elements [74]. The Stb1 protein is thought to enhance MBF transcription through binding to Swi6 [31].

MBF is regulated by a negative feedback loop through expression of the Nrm1 repressor. Nrm1 inhibition is counteracted by the Rad53 kinase, which phosphorylates Nrm1 and inhibits association of Nrm1 to Swi6 [162, 161]. Rad53 activity depends on ongoing DNA replication as described in Section 4.5. Only after DNA replication is finished, Rad53 is inactivated, resulting in the dephosphorylation of Nrm1, which can then bind to Swi6 and inhibit MBF activity [30].

Among the MBF regulated genes are *ACM1, SPC42, CLB5, CLB6, RAD53, NRM1, SCC1* and *PDS1* [111, 36, 91, 186].

Implementation

In the MBF module (Supplementary Figure 5), MBF-mediated transcription requires that MBF (Mbp1–Swi6) interacts with the MCB elements in the target promoters, as well as Cdc28–Cln1/2/3 mediated phosphorylation of Swi6 (*Swi6P*). Nrm1 inhibits MBF dependent transcription by binding to Swi6 [30]. Additionally, the model accounts for the binding of Stb1 to Swi6 as a positive effector on MBF transcription, and Stb1 phosphorylation by Cdc28 as a negative effector through decreased affinity for Swi6 [28].

The (unregulated) binding of MBF to MCB is not sufficient to explain the dynamic expression observed *in vivo*. However, literature research did not provide a fully conclusive mechanism of MBF regulation. Instead, we implemented a hypothetical requirement for Swi6 phosphorylation by Cdc28–Cln1/2/3 based on the study by Pic-Taylor *et al.* [114]. This hypothesis solves a technical problem, as the corresponding bipartite Boolean model requires absolute statements to convey signals. This link, however, cannot be a strict requirement *in vivo* as a Swi6 mutant with four alanine-substituted Cdc28 residues is viable [168]. Thus, the mechanistic regulation of MBF activation requires more experimental attention.

The influence of MBF on *RAD53* transcription is only activating (K+) instead of a requirement (!), to account for the constant presence of Rad53 throughout the CDC.

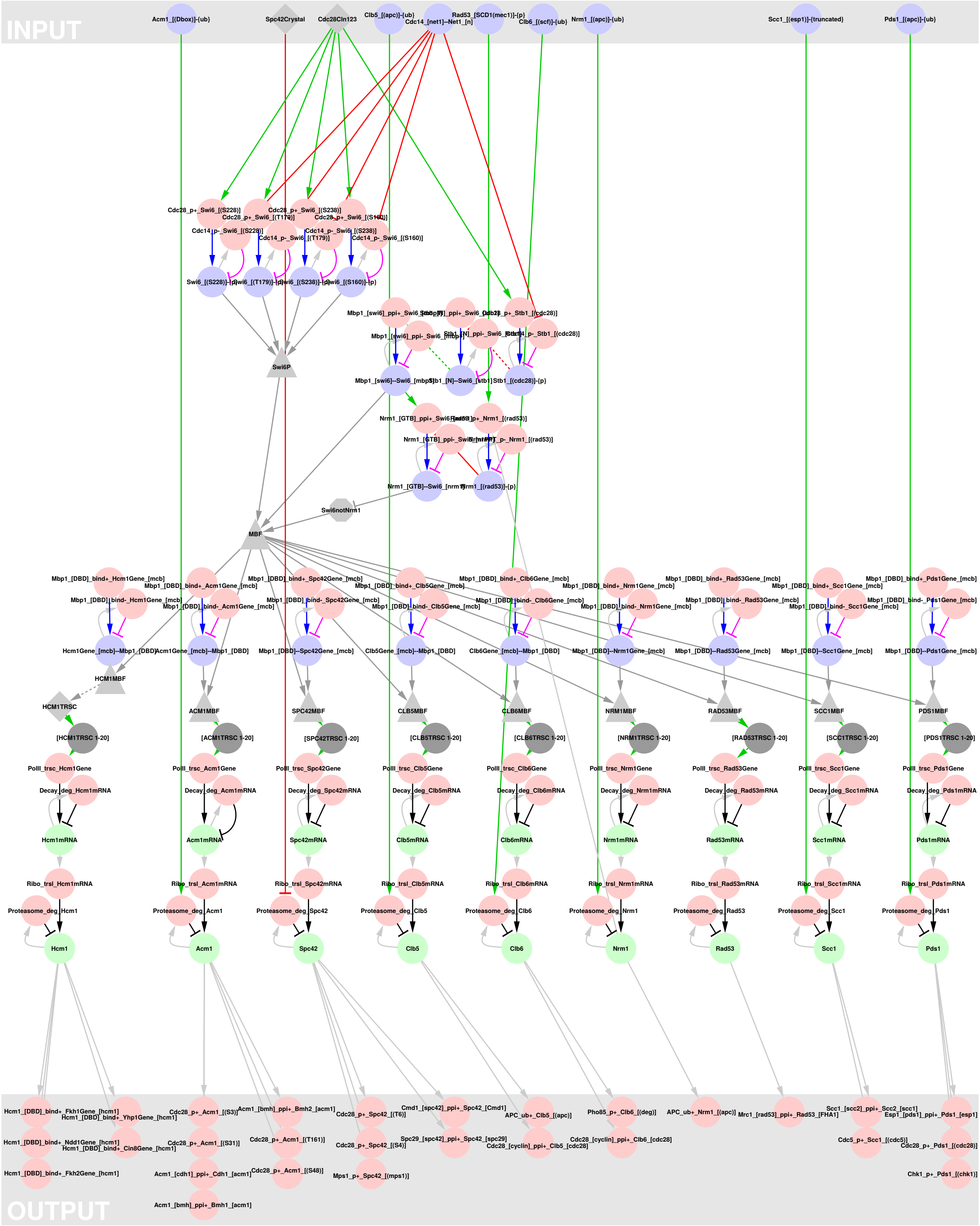

Supplementary Figure 5 *The MBF module.* MBF activates the transcription of *ACM1, SPC42, CLB5, CLB6, RAD53, NRM1, SCC1* and *PDS1*. MBF activation is positively regulated via Swi6, based on a model hypothesis. MBF activity is maintained while DNA replication is ongoing through Rad53 dependent phosphorylation of the repressor Nrm1. Rad53 is deactivated after DNA replication has finished, rendering Nrm1 unphosphorylated, enabling it to bind Swi6, resulting in the deactivation of the MBF cluster.

Interfaces

The MBF module is regulated by phosphorylation and dephosphorylation of Swi6 by Cdc28–Cln1/2/3 and Cdc14, respectively , and by the ubiquitylation status of Acm1, Clb5, Clb6, Nrm1, Scc1 and Pds1 , and by phosphorylation of Nrm1 by Rad53.

The cyclins Clb5 and Clb6 are required for initiation of DNA replication, while Rad53 monitors ongoing DNA replication (Section 4.5). Scc1 and Pds1 are involved in cohesin loading [94] during the chromosome cycle (Section 4.5). Acm1 is a pseudosubstrate inhibitor of the APC/C [32] and responsible for the inhibition of premature APC activation (Section 4.3.3). The protein Spc42 is an integral part of the SPB and used in the SPB replication cycle (Section 4.6).

4.2.5 The Hcm1 module

Biology

Hcm1 activates the transcription of *YHP1, CIN8, NDD1, FKH1* and *FKH2* [122]. Fkh1, Fkh2 and Ndd1 are themselves transcription factors that are necessary for the G2/M transition. *CIN8* encodes a kinesin motor protein [56] that is required for proper SPB separation (Section 4.6.4). Yhp1 is a transcriptional repressor of the upstream Mcm1 regulated cluster and thus, Yhp1 contributes indirectly to the deactivation of the Hcm1 cluster. Hcm1 is itself regulated by the transcription factors SBF and MBF [58, 11].

Implementation

In the Mcm1 module (Supplementary Figure 6), transcription of the target genes depends on Hcm1 binding, which in turn depends on the expression (existence) of Hcm1. All five targets genes included in the model are target of ubiquitylation, which is required for their degradation. Translation and mRNA decay are considered unregulated.

Interfaces

The Hcm1 module receives inputs from the SBF/MBF modules (*HCM1* expression) and the APC/C module (ubiquitylation). The module outputs are Yhp1, and inhibitor of Mcm1 transcription, Cin8, a kinesin motor protein, and the transcription factors *NDD1, FKH1* and *FKH2* that regulate the G2/M transition.

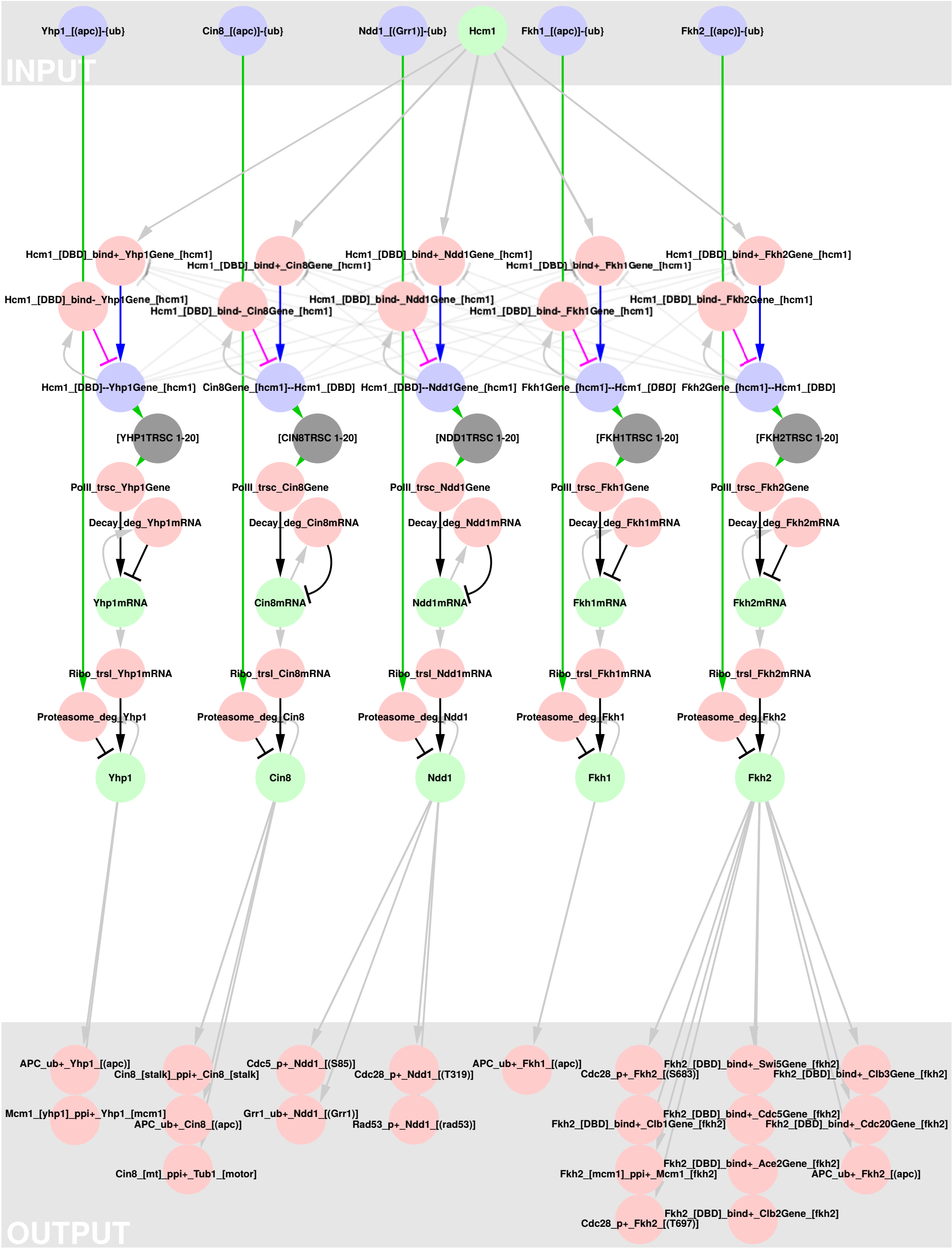

Supplementary Figure 6 *The Hcm1 module.* Hcm1 activates the transcription of *YHP1*, *CIN8*, *NDD1*, *FKH1* and *FKH2*. *HCM1* is regulated via SBF or MBF. The Hcm1 module is indirectly negatively regulated by transcription of the repressor Yhp1, which targets the upstream Mcm1 module.

4.2.6 The Fkh2 module

Biology

The Fkh2 gene cluster gates the transition to mitosis. The key target is *CLB2*, encoding the major cyclin driving mitotic entry. The target genes are activated by promoter recruitment of Fkh2 and Mcm1, and the recruitment of Ndd1 to Fkh2 [50]. The formation of the protein trimer Ndd1–Fkh2–Mcm1, represented by the *Fkh2Ndd1Mcm1* node, is regulated by Cdc28-mediated phosphorylation of Fkh2 and Ndd1 [120, 29]. The genes regulated by Fkh2, Ndd1, and Mcm1 (Supplementary Figure 7) considered in this study are the cyclins *CLB1,2,3*, the kinase *CDC5*, the APC/C regulatory subunit *CDC20*, and the transcription factors *ACE2* and *SWI5* [150, 50].

Similar to MBF regulation, Fkh2 cluster activation depends on the status of DNA replication. The phosphorylation of Ndd1 by Rad53 inhibits the association between Fkh2 and Ndd1 [180]. Upon termination of DNA replication, Rad53 is deactivated, Ndd1 becomes dephosphorylated at the Rad53 modified residue(s) and can bind Fkh2, which ultimately results in transcriptional activation of Fkh2Ndd1Mcm1 regulated genes. Activation of the Fkh2Ndd1Mcm1 cluster thus occurs after S phase.

Deactivation of the Fkh2 cluster is triggered by downregulation of Fkh2 and Ndd1, as well as the dephosphorylation of Fkh2 and Ndd1 towards the end of the cell cycle when Cdc14 is active.

Implementation

In the Fkh2 module (Supplementary Figure 7), phosphorylation of Fkh2 by Cdc28 at residues S683 and T697 is implemented as a strict requirement for the interaction between Fkh2 and Ndd1, and it requires either of the Clb1,2,5 or Clb6 cyclins [120]. Phosphorylation of Ndd1 by Cdc28–Clb1,2 [127] or Cdc5 [29] has a positive influence on Fkh2 transcriptional regulation. Thus, the transcriptional output of the Fkh2 cluster has a positive feedback loop via Clb1,2 [127].

The proteins activated by the Fkh2 cluster, except Ace2 and Swi5, are turned over in a regulated manner as described in Section 4.3.3.

Interfaces

The Fkh2 module depends on the expression levels (Section 4.2.5) and Cdc28 dependent phosphorylation (Sections 4.4.2 and 4.4.3) of Ndd1 and Fkh2, Rad53 dependent phosphorylation of Ndd1 (Section 4.5.5), Cdc5 dependent phosphorylation of Ndd1 (Cdc14 module), and APC/C-mediated ubiquitylation of Clb1, Clb2, Clb3, Cdc5 and Cdc20. The module output is the expression of Clb1, Clb2, Clb3, Cdc5, Cdc20, Ace2 and Swi5 . Clb1-3 are B-type cyclins that are essential for mitotic entry. Cdc5 is the Polo-like kinase with a critical role in mitosis. Cdc20 is a regulatory subunit of the APC/C. Swi5 and Ace2 are themselves transcription factors and described in Section 4.3.3.

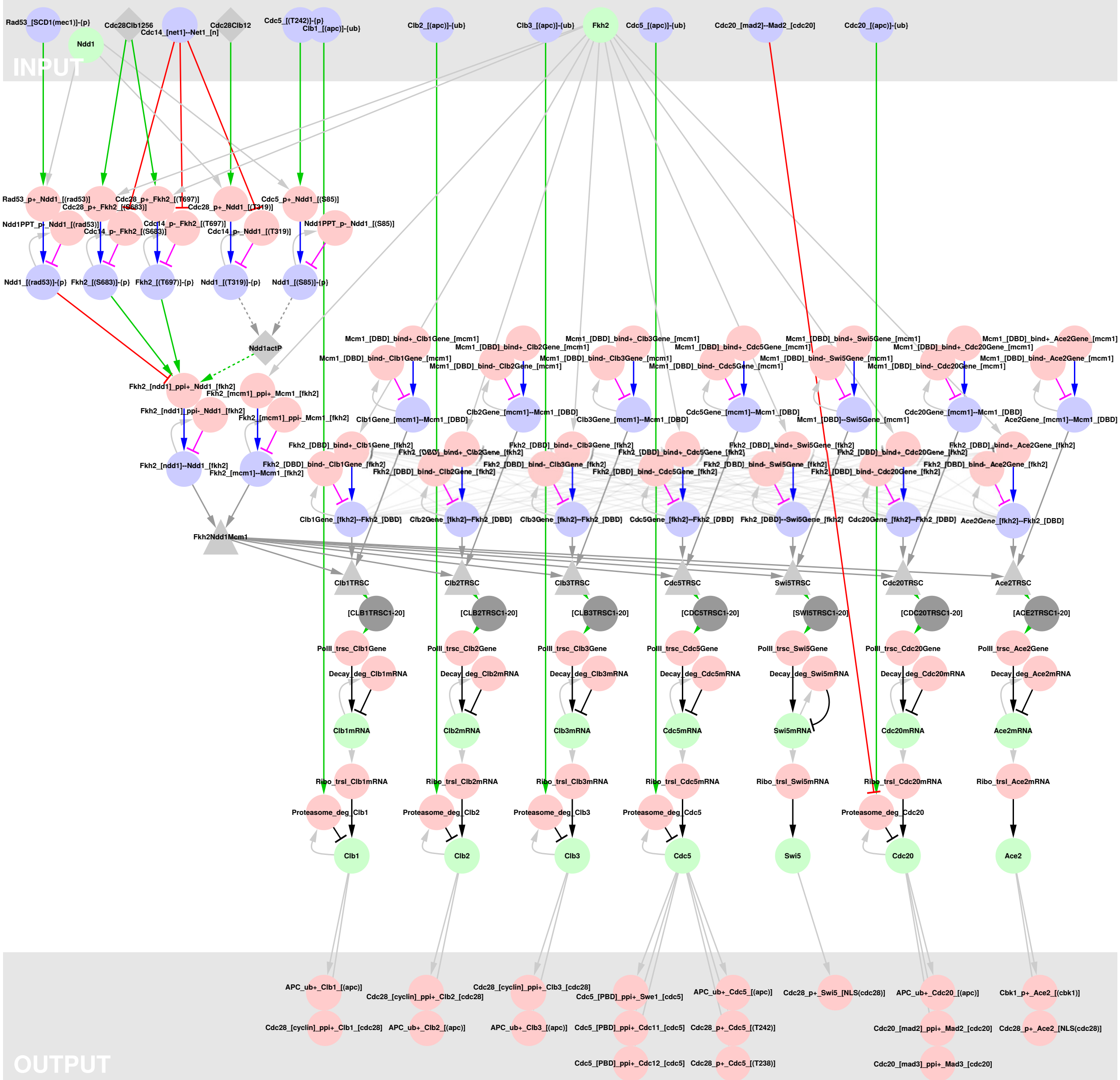

Supplementary Figure 7 *The Fkh2 module.* The model accounts for the transcription of *CLB1*, *CLB2*, *CLB3*, *CDC5*, *SWI5*, *CDC20* and *ACE2*, which are upregulated by the transcription factors Fkh2, Ndd1 and Mcm1. The Fkh2 cluster is positively influenced by the Hcm1 dependent transcription of *NDD1* and *FKH1*, and involvement of the kinases Cdc28 and Cdc5. The Rad53 kinase inhibits Fkh2Ndd1Mcm1 mediated transcription until DNA replication has finished.

4.2.7 Unregulated genes module

In addition to the above modules, we include five genes with constant transcription but whose gene products are targets of regulated degradation: *ASE1, CLB4, DBF4, FAR1, KIP1* and *MPS1* (Supplementary Figure 8). With the exception of Far1, no conclusive support could be found which demonstrates how these genes are regulated. However, these genes are involved in several cell cycle processes and expression must be included for all components that are turned over. Hence, this module has no real effect on the bBM simulation outcome (proteins will be constitutively expressed and hence present), but contains knowledge from literature.

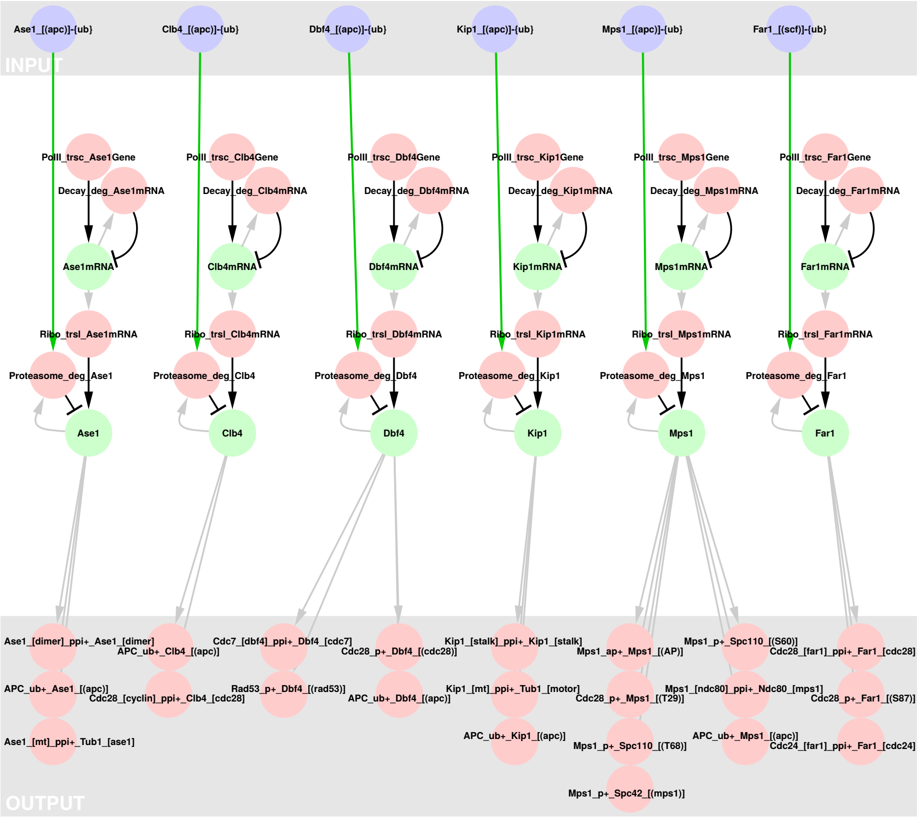

Supplementary Figure 8 *Unregulated genes module. ASE1, CLB4, DBF4, FAR1, KIP1* and *MPS1* are genes involved in cell cycle progression, but, except for *FAR1*, currently lack conclusively reported regulation.

4.3 Regulated degradation

This part describes regulated degradation by three proteasomal machineries and their regulation: The SCF machinery (Section 4.3.1), Dma1-mediated degradation (Section 4.3.2), and the APC/C machinery (Section 4.3.3).

4.3.1 SCF-mediated degradation module

Biology

SCF is a multimeric ubiquitin ligase that consists of the four core subunits Cdc34, Cdc53, Hrt1 and Skp1 [147], and which requires binding to an F-box protein to provide substrate specificity. Here, we account for the F-box proteins Cdc4, Grr1 and Met30 [151, 96, 69]. SCF recognises phosphorylated substrates, as e.g. in Sic1 [104, 109], priming them for degradation via the proteasome. However, in other cases, phosphorylation stabilises the target and inhibits its degradation. This applies to Mec1 phosphorylation of Swe1 at S385, which stabilises Swe1 during DNA replication [113].

Implementation

In the SCF-mediated degradation module (Supplementary Figure 9), ubiquitylation by SCF (*SCF* node) requires the core ligase, its binding to a targeting subunit (nodes *SCFCdc4*, *SCFGrr1*, *SCFMet30*) and the appropriate phosphorylation of the target substrate to generate the phospho-degron recognition sequences. Upon SCF-mediated ubiquitylation, the proteins are degraded by the proteasome. The module accounts for Sic1 stabilisation by phosphorylation at S385, preventing ubiquitylation and hence degradation.

Interfaces

The module depends on the expression of the target proteins and their phosphorylation by Cdc28, Pho85 and, in the case of Swe1, Mec1. The outputs of the model are the degradation of the target proteins.

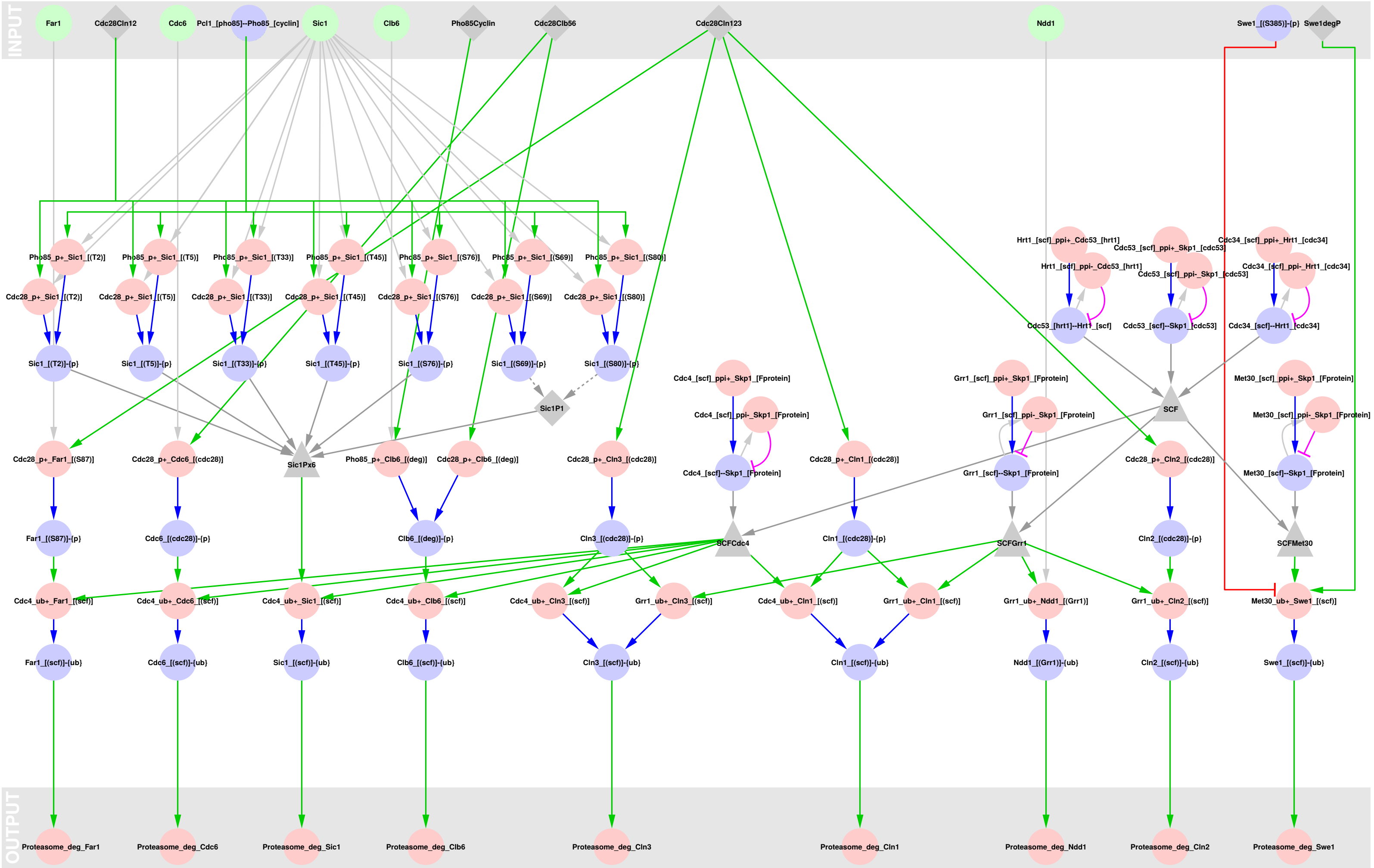

Supplementary Figure 9 *SCF-mediated degradation module.* The model accounts for the SCF and the three F-box proteins Cdc4, Grr1 and Met30, which provide substrate specificity. The SCF substrates considered here require phosphorylation for SCF recognition and subsequent degradation.

4.3.2 Dma1-mediated degradation module

Biology

Dma1 has been shown to ubiquitylate Pcl1 depending on prior phosphorylation by Pho85 [54], targeting Pcl1 for degradation. We hypothesise that this is also the mechanism for degradation of Pcl2 and Pcl9.

Implementation

In the Dma1-mediated degradation module (Supplementary Figure 10), Pcl1, Pcl2 and Pcl9 are continuously phosphorylated by Pho85-Pcl1/2/9, and consequently ubiquitylated by Dma1. Ubiquitylation by Dam1 primes Pcl1, Pcl2 and Pcl9 for degradation by the proteasome.

Interfaces

The module depends on Pho85-Pcl1/2/9. The output is the ubiquitylated states of the Pho85 cyclins and their subsequent degradation.

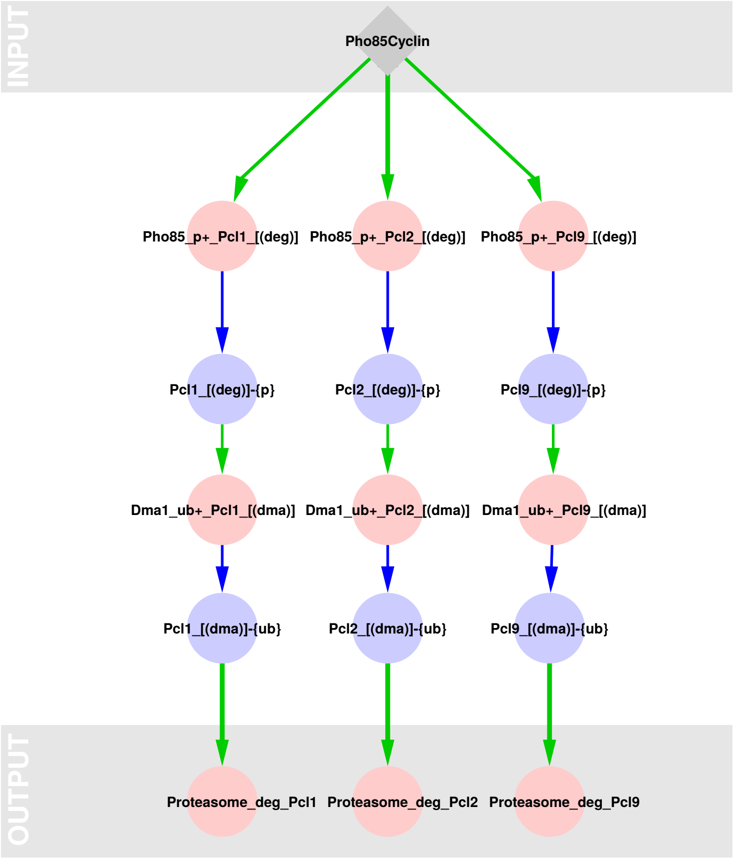

Supplementary Figure 10 *Dma1-mediated degradation module.* Pcl1, Pcl2 and Pcl9 are primed for degradation by ubiquitylation by Dam1. Dma1 recognition requires substrate phosphorylation by active Pho85.

4.3.3 APC/C-mediated degradation module

Biology

The APC/C is a multimeric ubiquitin ligase [144] that requires binding to a regulatory subunit, Cdc20 or Cdh1, for activity and substrate specificity. The APC/C has a third regulatory subunit, Ama1, which we do not consider as its function is thought to be limited to meiosis [41].

APC/C promotes progression through anaphase and its activity is tightly regulated, which is required to prevent premature cell cycle progression. Its activity is inhibited via pseudosubstrate binding of either of the two dimers Acm1–Bmh1 or Acm1–Bmh2 to Cdh1, blocking the substrate binding domain of Cdh1 [55, 32]. Acm1 is activated by MBF and phosphorylation by Cdc28–Cln1,2,3 during G_1 phase, but the contribution of each Acm1 phosphorylation has not been entirely determined [111]. Additionally, APC/C-Cdh1 activity is regulated by Cdc28–Clb5,6, which phosphorylates Cdh1 [36] and inhibits APC/C-Cdh1 activity [36, 172]. This inhibition is lifted by Cdc14-mediated dephosphorylation of Cdh1, allowing association between APC/C and Cdh1. APC/C-Cdc20 is kept inactive as long as Cdc20 is bound to Mad2, and Mad2 in complex with Mad1 and another Mad2 molecule [16], inhibiting Cdc20 interaction with the APC/C [16]. This interaction is regulated via a mechanism monitoring correct spindle alignment. Cdc20 itself is regulated by the Fkh2 module [50]. All ubiquitinated APC/C substrates are degraded by the proteasome.

In contrast to SCF targets, the APC/C targets seem to be stabilised by phosphorylation: The securin Pds1 is phosphorylated by Cdc28–Cln1,2,3 [2, 77], inhibiting its APC/C recognition [57]. Only after Cdc14 activation towards the end of the cell cycle, Pds1 is dephosphorylated and becomes susceptible for APC/C degradation. Mps1 phosphorylation has a negative influence on APC/C recognition [36], accounted for in the model.

Implementation

The APC/C-mediated degradation module (Supplementary Figure 11) accounts for regulation of the APC/C and priming of its targets for subsequent degradation. Although structural insights into APC/C topology have been reported [144], the information is insufficient to define it at the level of elemental states. Thus, the model accounts for the complex as a single component, *APC*, symbolising the complete multimeric APC/C without the regulatory subunit.

APC/C-Cdc20 is regulated by the recruitment of Cdc20 to APC/C. This in turn is inhibited by the bond between Cdc20 and Mad2, which tethers Cdc20 to the SPB. This bond in turn is released when the spindle is under stable tension.

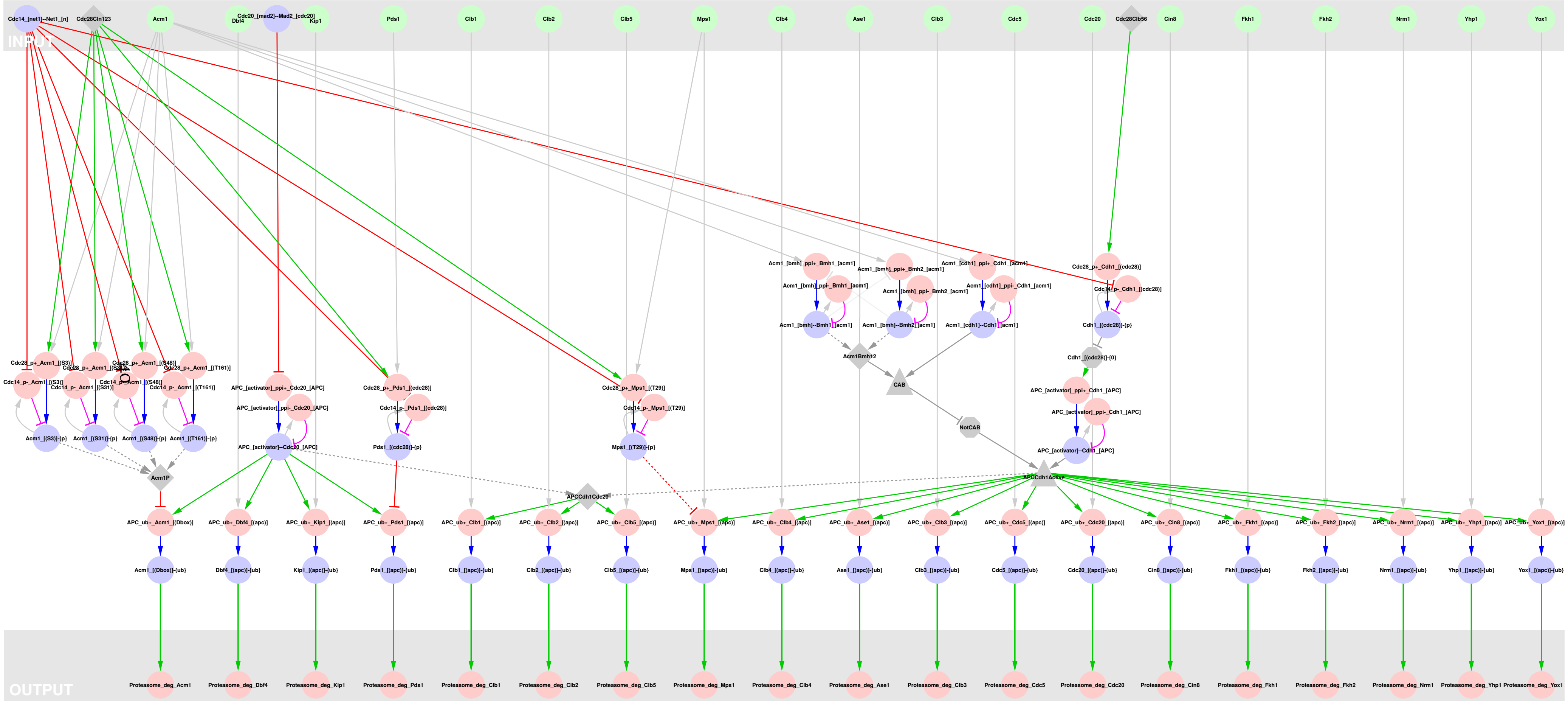

Supplementary Figure 11 *APC/C-mediated degradation module.* The model accounts for the APC/C regulatory subunits Cdc20 and Cdh1. APC/C–Cdh1 activity is inhibited by pseudosubstrate binding via Acm1, and furthermore by Cdc28-mediated phosphorylation. The phosphorylation is antagonised by Cdc14 towards the end of the cell cycle. APC/C-Cdc20 activity depends on the spindle assembly checkpoint (SAC).

APC/C-Cdh1 requires the recruitment of the regulatory subunit Cdh1 to APC, which is inhibited by phosphorylation by Cdc28-Clb5/6. In addition, the APC/C-Cdh1 complex can be inhibited by binding of Acm1 in complex with Bmh1 or Bmh2, implemented as *CAB*. This bond in turn is dependent on Acm1 availability only, and Acm1 is target for degradation by Cdc20 dependent on the phosphorylation status of Acm1. Cdc28-Cln1/2/3 phosphorylation stabilises Acm1 until the release of Cdc14 [35]. APC/C substrates accounted for here have transcriptional regulation and their degradation via the proteasome.

Interfaces

The APC/C module responds to phosphorylation of Acm1, Cdh1 and Mps1, and hence, depends on Cdc28-Cln1/2/3, Cdc28-Clb5/6 and Cdc14. Cdc20 recruitment depends on the Cdc20-Mad2 bond. Finally, the substrates must be present to be ubiquitylated.

The output of the module is the the ubiquitylation and subsequent degradation of the target proteins.

4.4 CDK assembly and activation

The two CDKs Pho85 and Cdc28 tightly regulate CDC progression. In turn, their activation is precisely timed and their substrates are dephosphorylated upon Cdc14 activation at mitotic entry. Section 4.4.1 describes the regulation of Pho85 and targets. Similarly, Section 4.4.2 describes activation of Cdc28 and its targets. Section 4.4.3 describes the regulation of Cdc14.

4.4.1 The Pho85 module

Biology

Pho85 is one of two CDKs that drive the cell division cycle. Pho85 requires cyclin binding in order to acquire kinase activity [95]. In contrast to the main CDK, Cdc28, Pho85 is dispensable for cell cycle progression. However, three of its cyclins show CDC dependent regulation expressed (Pcl1, Pcl2, Pcl9) and share redundant functions with the G_1-cyclins Cln1, Cln2 and Cln3. During G_1, Pho85 has a redundant role with Cdc28 in antagonising Whi5 and Sic1 [61]. Though it appears likely that Pho85 has a similar upstream regulation to Cdc28, empirical evidence is missing [110, 61].

Implementation

The Pho85 module (Supplementary Figure 12) accounts for Pho85 and its three cyclins Pcl1, Pcl2 and Pcl9. Pho85 is activated by binding to one of its cyclins Pcl1, Pcl2 or Pcl9. The Pho85-Pcl1,2,9 complexes have partially overlapping target specificities, which is accounted for in the model (nodes *Pho85Pcl1Pcl9* and *Pho85Cyclin*).

Interfaces

The module responds to expression of the Pcl1, Pcl2 and Pcl9 cyclins. The output of the module is phosphorylation of target proteins.

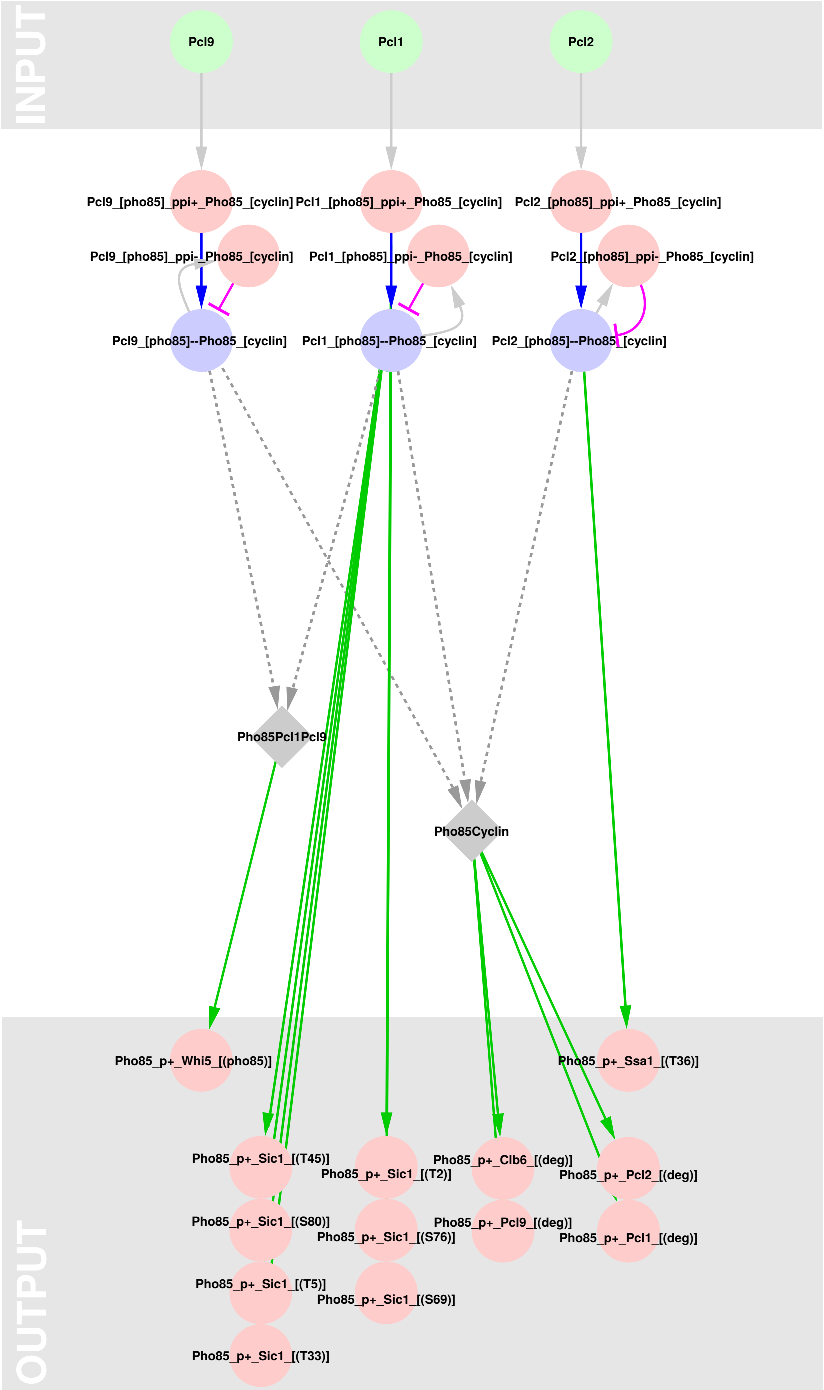

Supplementary Figure 12 *Pho85 module.* Pho85 binds to one of its activating cyclins Pcl1, Pcl2 or Pcl9, and phosphorylates Whi5, Sic1, Clb6, Pcl9, Ssa1, Pcl1 and Pcl2.

4.4.2 The Cdc28 module

Biology

The CDK Cdc28 is essential for cell cycle progression. Cdc28 activity depends on phosphorylation by Cak1 on residue T169, serving as a prerequisite for cyclin binding [137]. Cyclin binding to Cdc28 ensures substrate specificity. The different Cdc28-cyclin pairs promote different processes during the cell cycle and are differentially regulated, ensuring their temporally restricted activation. Nine cyclins activate Cdc28: Three G_1-cyclins, Cln1-3, and six B-type cyclins, Clb1-6.

There is functional redundancy between the cyclins [96]. In general, the cyclins can compensate for each other pairwise. These functional redundancies are supported by observations of single and multiple mutant phenotypes. The G_1 cyclins Cln1, Cln2 and Cln3 have redundant functions, and a triple deletion is lethal [128]. Similarly, the cyclin pair Clb1 and Clb2 has redundant functions, as the single mutants are viable, whereas a double mutant is lethal [156]. The cyclins Clb5 and Clb6 can also compensate for each other, as the single mutants are viable [80]. The double mutant is also viable, but the phenotype shows differences compared to the wild-type [80]. A mutational study indicates that the cyclin pair Clb3,4 can compensate for Clb5,6 functions [146].

The primary mechanism of regulation is cyclin expression. In addition, the Cdc28-Cln1/2/3 (G_1–cyclin) complexes require Cks1 binding to Cdc28 for activity [126]. Furthermore, Cln3 is regulated by the chaperone Ydj1 [177]. Ydj1 itself requires activation via Ssa1 or Ssa2, and Ssa1 is also a Cdc28 substrate [163], but the physiological relevance of this phosphorylation remains unclear. Furthermore, Cdc28 is regulated by Whi3, which binds to and restricts Cdc28 to the cytoplasm [170]. The interaction between Cln3 and Ydj1 and Cdc28 together relieves the interaction between Cdc28 and Whi3 [177].

The B-type cyclin complexes (Cdc28–Clb1/2/3/4/5/6) are also regulated by the CDKI Sic1, which binds to and inhibits the kinase activity of these complexes [96]. Sic1 in turn is antagonised by phosphorylation by the Cdc28-Cln1/2/3 and Pho85-Pcl1 [104] complexes, which primes it for degradation. Cdc28–Clb1,2 is additionally inhibited by phosphorylation of Cdc28 on residue Y19 by Swe1, which monitors bud emergence to prevent premature cell cycle progression.

In the presence of pheromone, Far1 binds the Cdc28-Cln1,2,3 complexes, thereby inhibiting Cdc28 kinase activity [118]. Thus, the cell cycle only reacts to pheromone treatment during G_1 phase when Cdc28 acts in collaboration with Cln1-3.

Implementation

The Cdc28 module (Supplementary Figure 13) accounts for Cdc28 regulation, as well as differential activation and target specificity by its cyclins. The module accounts for cyclin redundancy by assuming that cyclin pairs (Cln1/2; Clb1/2; Clb3/4 and Clb5/6) have the same targets, unless this has been explicitly shown not to be the case. The CDK-cyclin pairs and combinations are collected in

the nodes *Cdc28Cln12*, *Cdc28Cln123*, *Cdc28Clb12*, *Cdc28Clb56* and *Cdc28Clb1256*. The model accounts for Cdc28 activation by Cak1 phosphorylation at T169 as a prerequisite for cyclin binding. The model accounts for the regulation of Cln3 binding via a mechanism involving Ydj1 (node *Ydj1Active*), relieving the inhibitory bond between Cdc28 and Whi3 (effect of node *ERRelease* on Cdc28 and Whi3 dimerisation).

The mechanism of *CLB4* regulation has not yet been conclusively established. For this reason, the model does not account for Cdc28–Clb3,4 substrates, as this would render its targets constitutively phosphorylated, which is not observed.

The SCF substrate Swe1 inhibits Cdc28–Clb1,2 activity to prevent premature mitotic entry, and is itself regulated through degradation. The model takes two regulatory mechanisms for Swe1 degradation into account. First, Swe1 monitors bud morphology. Only after formation of a bud, Swe1 is phosphorylated and primed for degradation. Second, Swe1 is stabilised during DNA replication through Mec1 phosphorylation.

Sic1, another SCF substrate, inhibits Cdc28–Clb1,2,5,6 and requires phosphorylation by Cdc28–Cln1,2,3 or Pho85–Pcl1 for SCF recognition [104, 109]. Sic1 is phosphorylated at multiple residues as reported by Nash *et al.* [104]. These Sic1 phosphorylations at Cdc28 sites are hypothesised to be equivalent to Pho85 phosphorylation [109], with the same degradational consequences for Sic1. In the model, Sic1 is only a substrate of Cdc28–Cln1/2, as Cln3 activity is insufficient to target Sic1 for degradation.

The ubiquitylation of Sic1 and Swe1 are encoded as inhibitory for the binding to and phosphorylation of Cdc28, respectively. This is necessary for a dominant effect of ubiquitylation in the Boolean model, but may not be a necessary constraint in a quantitative model or *in vivo*.

The model also accounts for pheromone treatment by the input node *[Pheromone]*. The effect of pheromones is implemented as a dissociation block for Far1–Cdc28, depleting the Far1 free and (potentially) active form of Cdc28-Cln1,2,3.

Interfaces

The primary input to the Cdc28 module is the expression of the cyclins, the CDKIs Far1 and Sic1, and Swe1. In addition, it responds to pheromones and to the ubiquitylation status of Sic1 and Swe1, which are considered to be inhibited when ubiquitylated.

The module output is the phosphorylated states of the Cdc28 substrates.

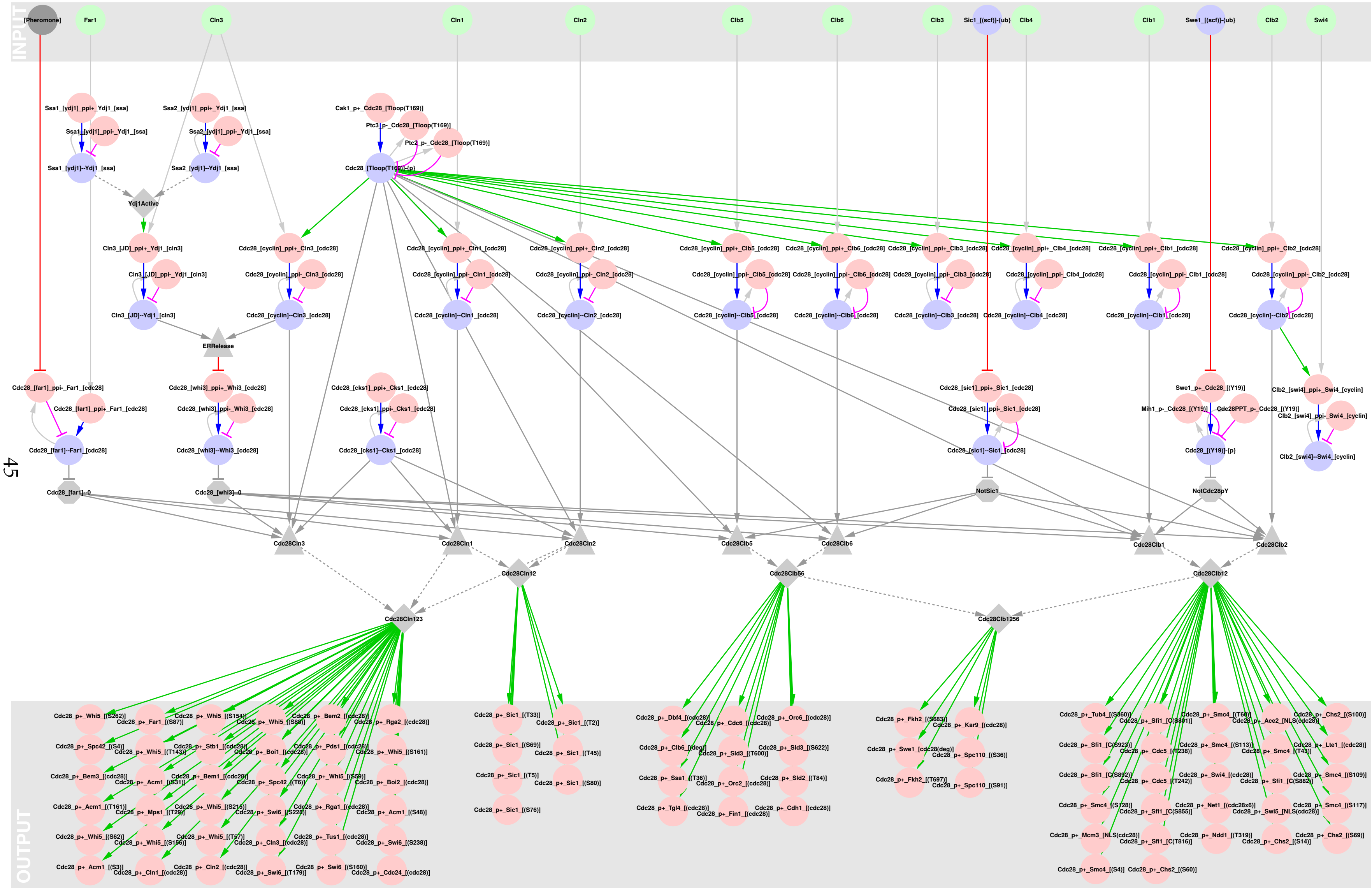

Supplementary Figure 13 *Cdc28 module.* Cdc28 activity is controlled by T169 phosphorylation by Cak1, dimerisation with one of nine different cyclins (Cln1-3; Clb1-6), two distinct CDKIs, Swe1 and Sic1, and Y19 phosphorylation by Swe1. The module accounts for Cdc28 target specificity by differential phosphorylation of Cdc28 targets dependent on the activating cyclin.

4.4.3 The Cdc14 module

Biology

The phosphatase Cdc14 antagonises Cdc28 and Pho85-mediated phosphorylations. The dephosphorylation of Cdc14 targets is temporally regulated and only occurs towards the end of the cell cycle. Cdc14 is kept inactive through sequestration by Net1, which retains Cdc14 at the nucleolus [148]. This interaction is relieved by phosphorylation of Net1 by Cdc28–Clb1,2 and Cdc5, as well as phosphorylation of Cdc14 by the kinase Dbf2 [98]. Dbf2 is activated upon correct SPB positioning, involving Cdc15 and Nud1 [172, 132, 92], as described in Section 4.7.4. The activational order of phosphorylation of Cdc14 by Cdc28 and Cdc5 is controversial, as the studies by [125] and [8] show. Net1 is dephosphorylated by PP2A [125].

Implementation

The Cdc14 module (Supplementary Figure 14) accounts for Cdc14 and Cdc5 regulation. Cdc14 bound to Net1 implies Cdc14 retention at the nucleolus [148] and hence, inhibits Cdc14 substrate access. Relief of the interaction between Cdc14 and Net1 requires phosphorylation of Net1 by Cdc28 and Cdc5, where Cdc28-Clb1,2 phosphorylates Net1 at six unspecified sites, priming it for phosphorylation by Cdc5, as based on [125]. These phosphorylations are combined in the node *Net1PP*. Cdc5 is activated by Cdc28-Clb1/2-mediated phosphorylation on residue T242 [133]. The model also takes Dbf2-mediated phosphorylation of Cdc14 into account. Dbf2 activation is regulated by Cdc15 as described in Section 4.7.4.

Interfaces

The Cdc14 module responds to Cdc5 expression, Cdc28-Clb1/2 kinase activity, Dbf2 activity and the phosphatase PP2A. The module output is phosphorylation of Cdc5 substrates and dephosphorylation of Cdc14, and hence, Cdc28 and Pho85, targets.

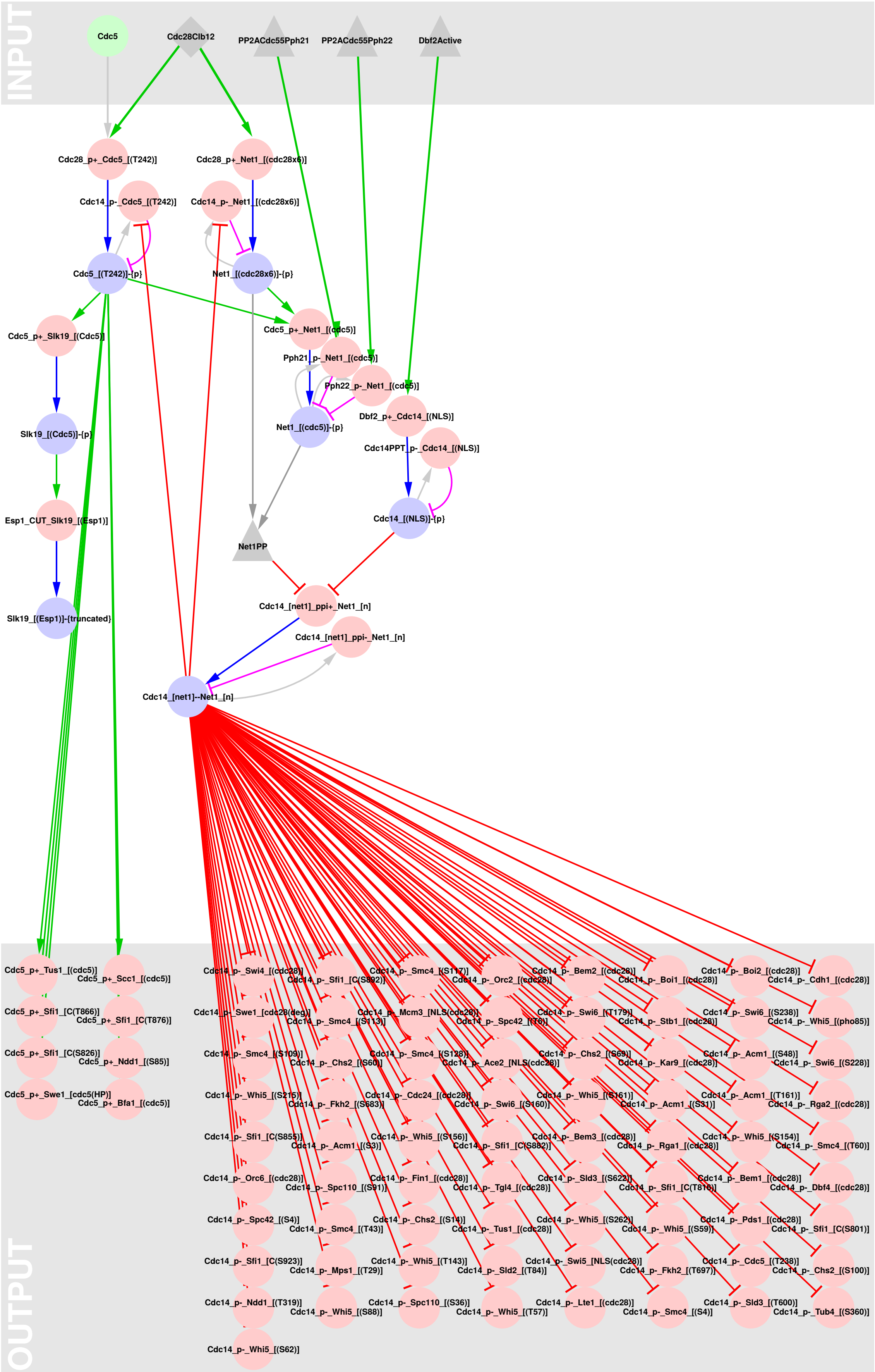

Supplementary Figure 14 *Cdc14 module.* Cdc14 is retained and kept inactive at the nucleolus, through its interaction with Net1. Release from the nucleolus requires Net1 phosphorylation by Cdc28–Clb1,2 and Cdc5, and Cdc14 phosphorylation by Dbf2 in response to the spindle position checkpoint (SPOC, Supplementary Figure 27). Cdc14 activation leads to the dephosphorylation of Cdc28 and Pho85 substrates.

4.5 DNA replication

DNA replication is the first of three macroscopic duplication processes that are covered by the CGM. We break this process down into four distinct steps: DNA licensing (Section 4.5.1), DNA replication initiation (Section 4.5.2), DNA replication (Section 4.5.5), and sister chromatid separation (Section 4.5.6).

4.5.1 DNA licensing

Biology

DNA licensing requires the recruitment of two complexes, the Origin recognition complex (ORC), and the Mcm2–7 helicase complex. ORC consists of six subunits, Orc1-6, and binds to origin DNA via Orc1 [102]. ORC in turn recruits the helicase Mcm2–7 to the origins via interaction between Orc1 and Cdc6, as well as Cdt1 and Cdc6 [12].

Recruitment of ORC is restricted to a time window before Cdc28–Clb5,6 becomes active, as the phosphorylation of Orc2 and Orc6 by Cdc28-Clb5/6 inhibits ORC recruitment to the origins [108]. Relicensing is additionally inhibited by Cdc28–Clb1/2 phosphorylation of Mcm3, inhibiting nuclear localisation of the Mcm2–7 complex [84]. Binding between Cdt1 and Orc6 is the crucial step to link the Mcm2–7 complex to the ORC complex, resulting in helicase loading at the origins.

Implementation

The DNA replication cycle starts with the macroscopic *DNA licensing* reaction *Cell_LIC_Cell_[(DNA)]* (Supplementary Figure 15). The model accounts for regulation of helicase recruitment, but not explicitly for helicase dimerisation [40]. Instead, it is assumed that dimerisation occurs upon recruitment of the Mcm2–7 complex. Successful loading of the helicase to the origins is implemented as output *[HelicaseLoading]*, which is a requirement for the *Cell_LIC_Cell_[(DNA)]* reaction to execute. This reaction produces the *Cell_[(DNA)]-{lic}* state, representing licensed DNA.

Interfaces

CDC6 and *MCM2-7* are regulated by the Mcm1 module, although only Cdc6 turn-over is considered in the model as Mcm2-7 are constantly present and regulated through nuclear exclusion.

In addition to Cdc6 expression, the module is regulated by the phosphorylation of Orc2, Orc6 and Mcm3.

The output of the module is *[HelicaseLoading]*, which changes the DNA status to licensed DNA.

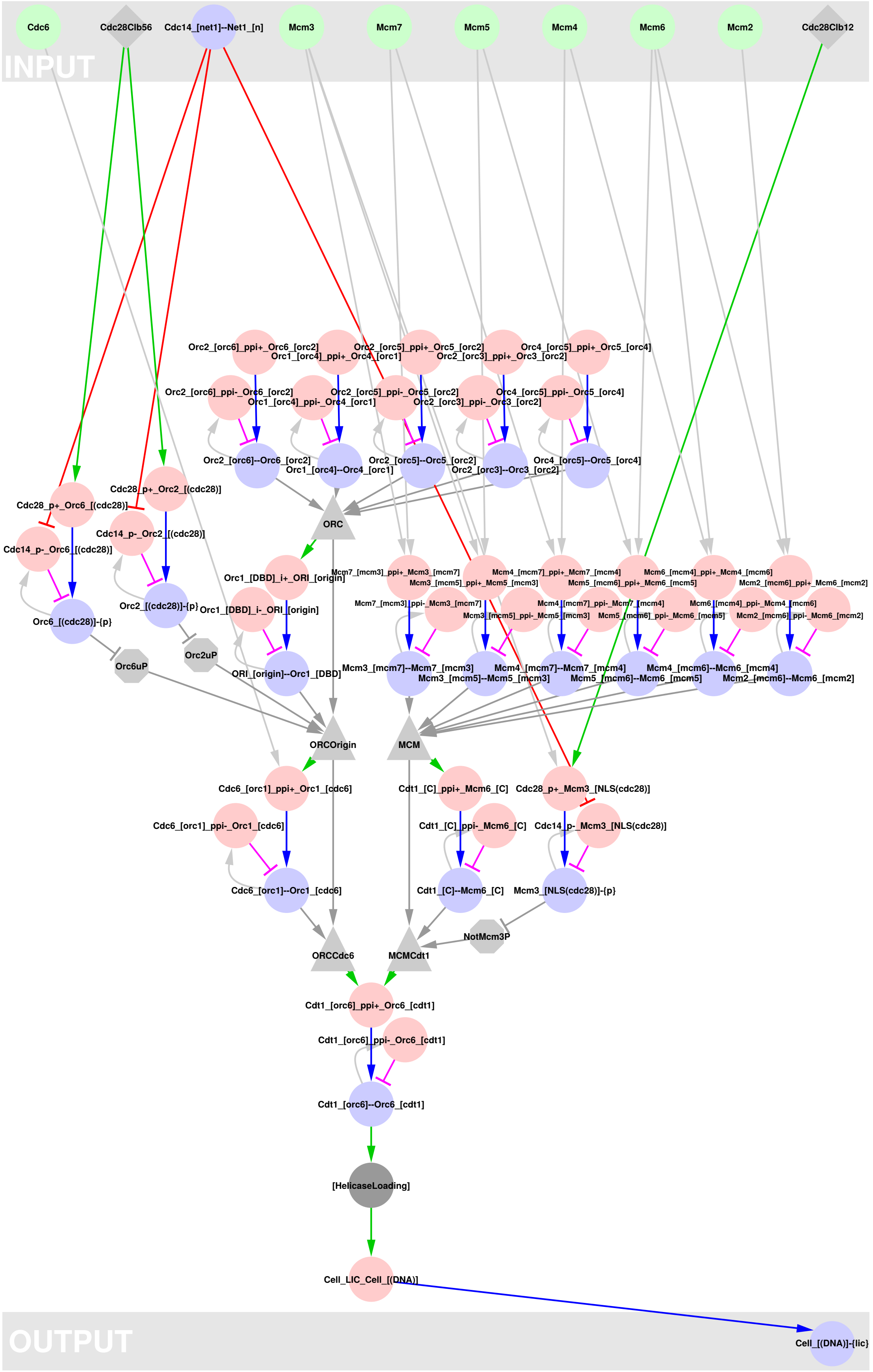

Supplementary Figure 15 *DNA licensing module.* DNA licensing requires recruitment of the ORC complex and the Mcm2-7 complex. Licensing is restricted to a time window between deactivation of Cdc28-Clb1/2 and the activation of Cdc28-Clb5/6, as phosphorylation of Orc2, Orc6 and Mcm3 prevents licensing.

4.5.2 DNA Replication initiation

4.5.3 Biology

DNA replication can only be initiated upon formation of licensed DNA. In this step, several factors are recruited to the licensed origins: Cdc45 and Sld3 are recruited to the MCM helicase via Mcm4 and Mcm6, resulting in assembly of the MCM-Cdc45-Sld3 complex. This recruitment strictly depends on phosphorylation of Mcm4 and Mcm6 by the Dbf4–dependent kinase (DDK) Cdc7 [12].

The MCM-Cdc45-Sld3 complex constitutes the platform for the recruitment of Dpb11, Pol2, Psf1 and Sld2 to form the Cdc45/Mcm2–7/GINS (CMG) complex [12]. CMG assembly is further regulated by Cdc28-Clb5,6-mediated phosphorylation of Sld2 and Sld3 [89, 36], allowing their binding to Dpb11 [179, 12]. Origin firing also requires Mcm10 [140, 90].

4.5.4 Implementation

The DNA replication initiation module (Supplementary Figure 16) accounts for the recruitment of additional factors to the licensed origins in order to prepare DNA replication. This step is implemented with the macroscopic *Replication initiation* reaction *Cell_RepInit_Cell_[(DNA)]*. The model accounts for the role of the DDK Cdc7 in helicase assembly. DDK activity has no regulation in the model and is permanently present, as it is the output of the unregulated *DBF4* gene. A possible cell cycle regulated transcription pattern of *DBF4*, which may contribute to DDK regulation, is disputed [154]. DDK substrates, however, may require priming by other kinases [12]. The timely regulation by DDK is ensured in the model by the dependence of licensed DNA, i.e. the phosphorylation of Mcm4 and Mcm6 only occurs in the context of the loaded double hexamer.

Mcm10 stimulates helicase activity, which the model accounts for by making its recruitment via Mcm2 a requirement for origin firing.

Upon recruitment of all necessary factors and formation of the CMG complex (node *CMG*), origin firing starts, implemented as the output node *[OriginFiring]* as a requirement for the reaction *Replication initiation*. This reaction produces the *Cell_[(DNA)]-{replicating}* state, which represents ongoing DNA replication.

Interfaces

The module is regulated by (de)phosphorylation of Mcm4, Mcm6, Sld2 and Sld3, which depends on DDK, and Cdc28-Clb5/6.

The output of the model is *[OriginFiring]* and DNA replication initiation.

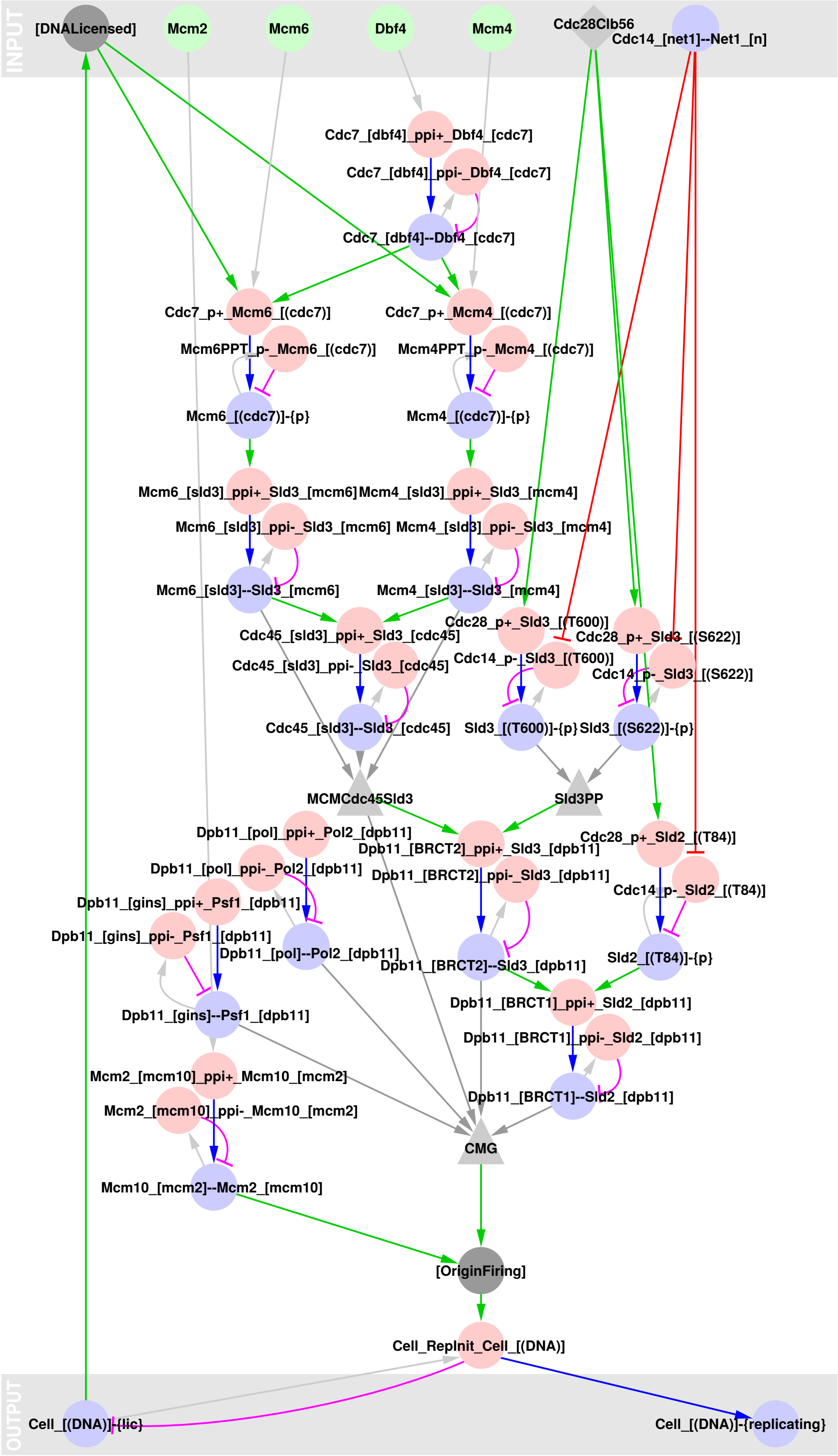

Supplementary Figure 16 *DNA replication initiation module.* Cdc7–Dbf4 and Cdc28–Clb5/6 initiate the recruitment of Cdc45, Sld2, Sld3, Dpb11, Pol2 and Psf1 to licensed origins. The recruitment of these and additional factors leads to the establishment of the CMG complex, which together with Mcm10 initiates origin firing.

4.5.5 DNA replication module

Biology

DNA replication starts upon replication initiation. Ongoing DNA replication creates replication forks, which are characterised by single-stranded/double-stranded (ss/ds) DNA junctions. The proteins Rfa1-3 form the RPA complex [63], which is recruited to ssDNA via Rfa1 and Rfa2 [63]. RPA is recruited to ssDNA together with the DNA replication checkpoint proteins Lcd1 and Mec1 [169, 139].

The replication forks recruit several additional replication factors, including the replication factor C (RFC), Ctf18 and Mrc1 [24, 46]. The RFC complex binds to ssDNA at the ssDNA/dsDNA junctions. RFC in turn recruits Ctf18 and binds to Pol2, the catalytic subunit of DNA polymerase II. The assembly of this complex together with Lcd1-Mec1 recruitment to ssDNA has the potential to bring Mec1 and Mrc1 together and is hypothesised to be the key regulatory step in Mec1/Rad53 signalling based on [46] and [53].

Mec1/Rad53 signalling proceeds through a phosphorylation cascade. First, Mec1 phosphorylates Mrc1 [115, 23]. Phosphorylated Mrc1 binds to Rad53 [23], a prerequisite for Mec1 phosphorylation of Rad53 [23]. This phosphorylation by Mec1 activates Rad53 [22], which in turn signals ongoing DNA replication by phosphorylating its substrates. Thus, Rad53 is activated and remains active during DNA replication.

Active Rad53 phosphorylates and inhibits the MBF repressor Nrm1 [162], maintaining MBF regulated transcription until DNA replication has finished. Simultaneously, Rad53 phosphorylates Ndd1 [22], inhibiting activation of the genes regulated by Ndd1, Fkh2 and Mcm1. These phosphorylations occur in the context of activated Rad53 [22]. Furthermore, activated Rad53 phosphorylates Dun1 [22], which in turn phosphorylates Sml1 [23, 115]. Phosphorylated Sml1 dissociates from the ribonucleotide reductase (RNR) Rnr1, thereby allowing RNR activity, which is required for synthesis of deoxynucleoside triphosphate (dNTP). dNTP synthesis requires the activation of the DNA replication checkpoint through Mec1/Rad53 dependent signalling from single stranded DNA.

In parallel, Mec1 phosphorylation stabilises Swe1 [113], providing an additional mechanism for inhibition of mitotic entry. DNA replication also requires histone synthesis to package the replicated genome.

Implementation

The DNA replication module (Supplementary Figure 17) accounts for the completion of DNA replication as well as activation of the signalling cascade monitoring ongoing DNA replication via Mec1/Rad53. This module regulates the macroscopic *DNA replication termination* reaction *Cell_RepFinish_Cell_[(DNA)]*. The state *Cell_[(DNA)]-{replicating}* from the previous module is a prerequisite to create replication forks leading both to exposed ssDNA and ssDNA/dsDNA junctions, which are implemented as the nodes *[ssDNA]* and *[ssdsDNAjunctions]*. To express the dependence on RPA recruitment to ssDNA, the interaction

between Rfa1,2 and ssDNA additionally requires the *[ssDNA]* node.

The existence of ss/ds DNA junctions induces the recruitment RFC which associates with additional factors, including the polymerase II catalytic subunit Pol2. The different complexes are represented by the nodes *RPA*, *RPAssDNA*, *RPAssDNALcd1Mec1*, and *DNARFCCtf18Pol2Mrc1*. Activation of the Mec1-Mrc1-Rad53 pathway requires the assembly of both the RFC-Crf18-Pol2-Mrc1 complex at replication forks, and the assembly of the RPA-Lcd1-Mec1 complex on ssDNA. The simultaneous recruitment of these complexes triggers Mec1-Mrc1 interaction and pathway activation. This phosphorylation of Mrc1 triggers recruitment of Rad53 and a phosphorylation cascade over Rad53 and Dun1 to Sml1. Phosphorylation of Sml1 disrupts its binding to Rnr1, relieving its inhibition of the RNR complex, and allowing the dNTP (*[dNTP]* node) synthesis required for DNA replication. This cascade also leads to Swe1 phosphorylation at S385 by Rad53, thereby inhibiting Swe1 ubiquitylation and preventing its degradation.

The model does not explicitly account for histones due to limited mechanistic knowledge on the regulation of histone synthesis and assembly, and uses instead *[Histones]* as input to the macroscopic DNA replication reaction. This placeholder needs to be true in any Boolean simulation of the network model. The model accounts for the effects of hydroxyurea (HU) by inhibiting dNTP synthesis, and thereby preventing DNA replication, leading to cell cycle arrest in the S-phase. A time-scale adjustment was introduced to the replication module to make DNA replication slower than Mec1/Rad53 signalling.

The *Cell_RepFinish_Cell_[(DNA)]* reaction produces the state *Cell_[(DNA)]-{replicated}*, which represents completed DNA replication. Simultaneously, the *Cell_[(DNA)]-{replicating}* state disappears and along with it, the indicators of ongoing DNA replication (ssDNA, ssDNA/dsDNA junctions). This deactivates the DNA replication pathway and leads to the inactivation of MBF regulated transcription, and to the relief of Ndd1 inhibition which makes the transcription of the genes regulated by the Fkh2 module possible.

Interfaces

The DNA replication module depends on ongoing replication, and the ssDNA and ssDNA/dsDNA junctions that trigger the Mec1/Rad53 signalling cascade.

The output is a completely replicated genome, represented by the state *Cell_[(DNA)]-{replicated}*.

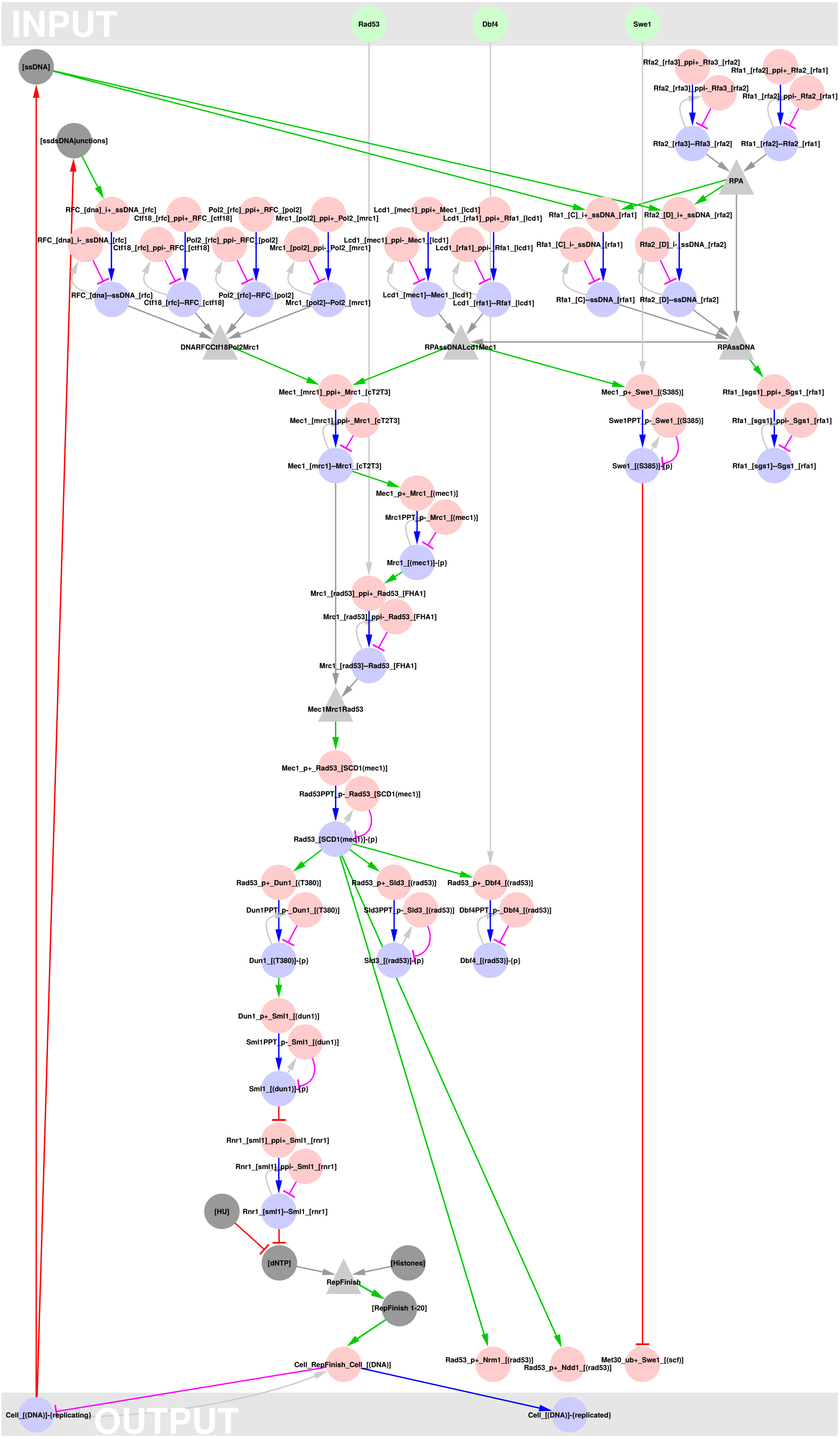

Supplementary Figure 17 *DNA replication module.* Ongoing DNA replication creates replication forks and hence both ssDNA and ss-DNA/dsDNA junctions. Replication checkpoint proteins are recruited to these replication forks, leading to the activation of the kinase Rad53. The checkpoint is active until DNA replication has finished.

4.5.6 DNA separation module

Biology

The two sister chromatids created through DNA replication are held together by cohesin rings. In anaphase, these cohesin rings must be destroyed to allow separation of the sister chromatids and ultimately nuclear division (ND). Correct chromosome separation and segregation is a prerequisite for chromosome stability. Hence, DNA separation must be performed in the correct cell cycle phase to ensure survival of the cell.

Disintegration of the cohesin ring requires proteolytic cleavage of the Scc1 subunit. Scc1 is cleaved by the separase Esp1 [164]. Scc1 cleavage is regulated both by Scc1 phosphorylation and Esp1 availability in order to accomplish timely cohesin ring destruction. Esp1 is inhibited by binding to Pds1 [26, 164]. This inhibition is relieved by APC/C-mediated degradation of Pds1 (Section 4.3.3). *PDS1* is MBF regulated and the protein Pds1 becomes phosphorylated by Cdc28–Cln1/2/3 in G_1 phase, which protects it from degradation. Upon activation of Cdc14, Pds1 is dephosphorylated and can be degraded. Scc1 must furthermore be phosphorylated by active Cdc5 [4, 133]. These processes are restricted to a time window with Cdc14 activity, during which it is assured that DNA replication has finished and the bipolar spindle is correctly aligned and under tension (Section 4.6).

Implementation

The DNA separation module (Supplementary Figure 18) accounts for assembly and disintegration of the cohesin rings, allowing for sister chromatid separation and ND. The model does not explicitly account for chromatids. Instead, the model describes the regulation of the cohesin rings and their impact on the two macroscopic reactions *Cell_SEP_Cell_[(DNA)]*, the *DNA separation* reaction, and *Cell_ND_Cell_[(SPB)]*, the *Nuclear division* reaction. The model accounts for cohesin ring assembly with two steps. First, in an initial cohesin loading step, combined in the *CohesinLoading* node, the cohesin ring subunits Scc1, Scc2, Scc4 are recruited. These associate with each other and bind to centromeric regions of the DNA via Scc4 [94]. Second, the subunits Irr1, Smc1 and Smc3 are recruited, forming the complete ring, combined in the node *[CohesinRing]*.

The model accounts for the regulation of cohesin ring destruction with two prerequisites: phosphorylation of Scc1 by active Cdc5, and activation of the separase Esp1, by degradation of its inhibitor Pds1. Esp1 truncates Scc1, thereby destroying the *[CohesinRing]*. This allows the *DNA segregation* and *Nuclear division* reactions to execute, resetting the macroscopic states of the DNA and SPB cycles to their neutral values.

Interfaces

The DNA separation module depends on the expression of Scc1 and Pds1 and the activity of Cdc5. The output of the model is destruction of the *[CohesinRing]*, which is required for both DNA separation and ND.

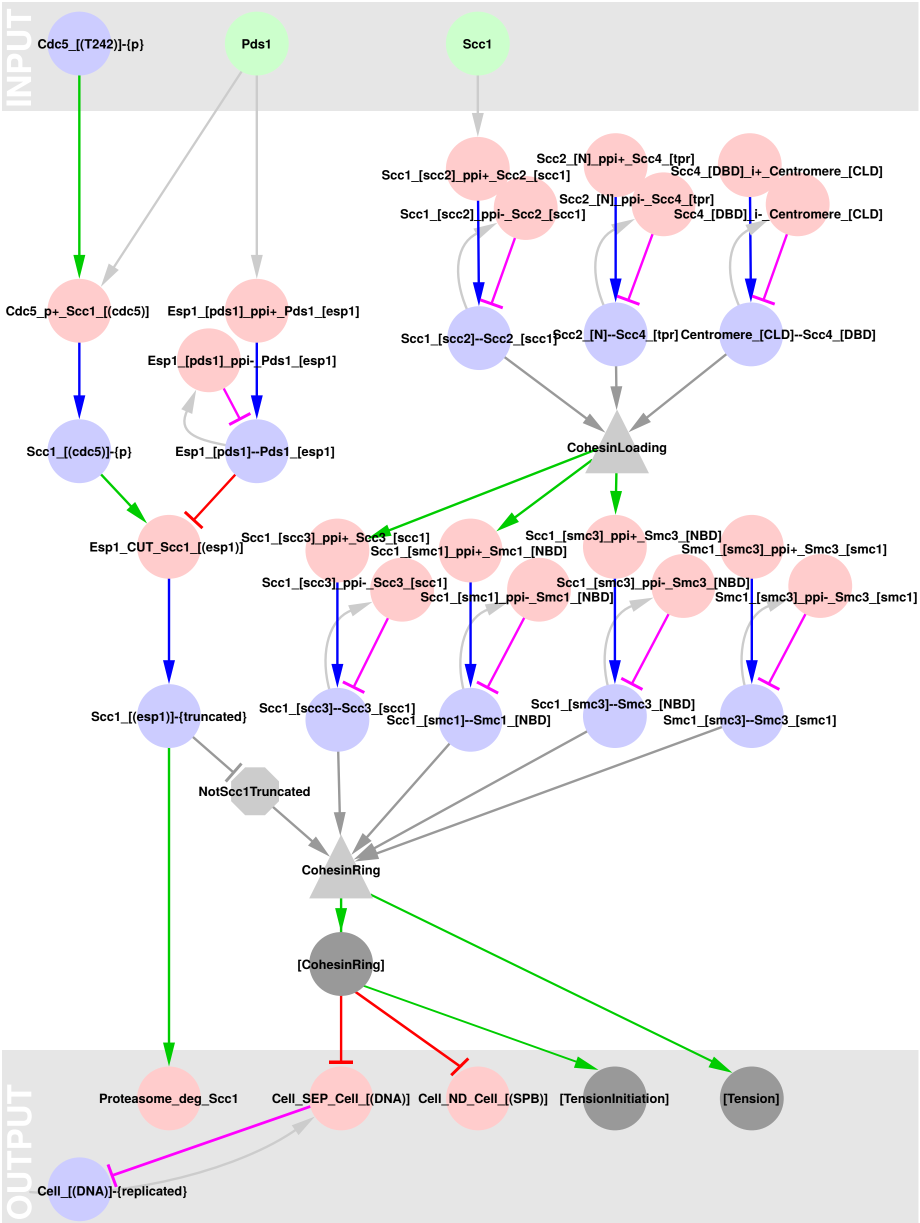

Supplementary Figure 18 *DNA separation module.* DNA separation requires the disassembly of the cohesin ring. This is regulated by activation of Esp1, which truncates the cohesin ring subunit Scc1, leading to the disintegration of the cohesin ring. Upon DNA separation, the sister chromatids can be separated, which is a prerequisite for nuclear division (ND).

4.6 SPB duplication and nuclear division

The spindle pole bodies (SPBs) are the microtubule organising centres in baker's yeast. As yeast has a closed mitosis, the SPBs must be embedded in the nuclear envelope. During G_1, a single SPB with short cytoplasmic and nuclear microtubule MTs holds the centrosomes of the unreplicated genome and positions the nucleus. The SPB is duplicated during S-phase and the two SPBs separate as the cells enter mitosis to form a bipolar spindle. Bipolar attachments of sister chromatids stabilise the MTs, and once the spindle is properly formed (as determined by tension at all kinetochores) and positioned (one SPB in the mother, one in the bud), the cell rapidly separates the chromosomes and undergoes nuclear division. We divide these processes into five modules: SPB satellite formation (Section 4.6.1), SPB duplication plaque formation (Section 4.6.2), SPB duplication (Section 4.6.3), SPB separation (Section 4.6.4), and SPB tension establishment (Section 4.6.5).

4.6.1 SPB satellite formation module

Biology

In this first step, the half-bridge at the inherited SPB, formed by Sfi1 molecules with free C-termini, transforms into a full bridge by recruiting a second set of Sfi1 molecules. The Sfi1 molecules bind C-terminal to C-terminal, thereby forming the bridge [140]. The Sfi1 dimerisation is controlled by Cdc5 and Cdc28-Clb1/2 phosphorylation of Sfi1, so that SPB duplication only occurs once per cell cycle [34].

There are six Cdc28 sites in Sfi1 which are thought to contribute to the inhibition of Sfi1 dimerisation [34]. The kinase Mps1 also appears to be involved in regulation of Sfi1 dimerisation [34]. However, its role in bridge formation and its regulation on a mechanistic level is not yet established. Along with Sfi1 recruitment, the additional components Cdc31 and Kar1 associate with the bridge [140].

Implementation

The SPB satellite formation module accounts for the transformation of the half-bridge at the inherited SPB into a bridge, priming it for satellite formation (Supplementary Figure 19). The *SPB satellite initiation* reaction *Cell_SAT_Cell_[(SPB)]* refers to this bridge formation, priming it for satellite formation by producing the *Cell_[(SPB)]-{sat}* state. The Sfi1 dimer, together with Cdc31 and Kar1, constitutes the bridge, combined in the node *[Bridge]*, forming the prerequisite for the *Cell_SAT_Cell_[(SPB)]* reaction to execute.

Interfaces

The SPB satellite formation module depends on the (de)phosphorylation of Sfi1 by Cdc5, Cdc14 and Cdc28-Clb1/2. The output of the model is the bridge, which is a prerequisite for the formation of the SPB satellite.

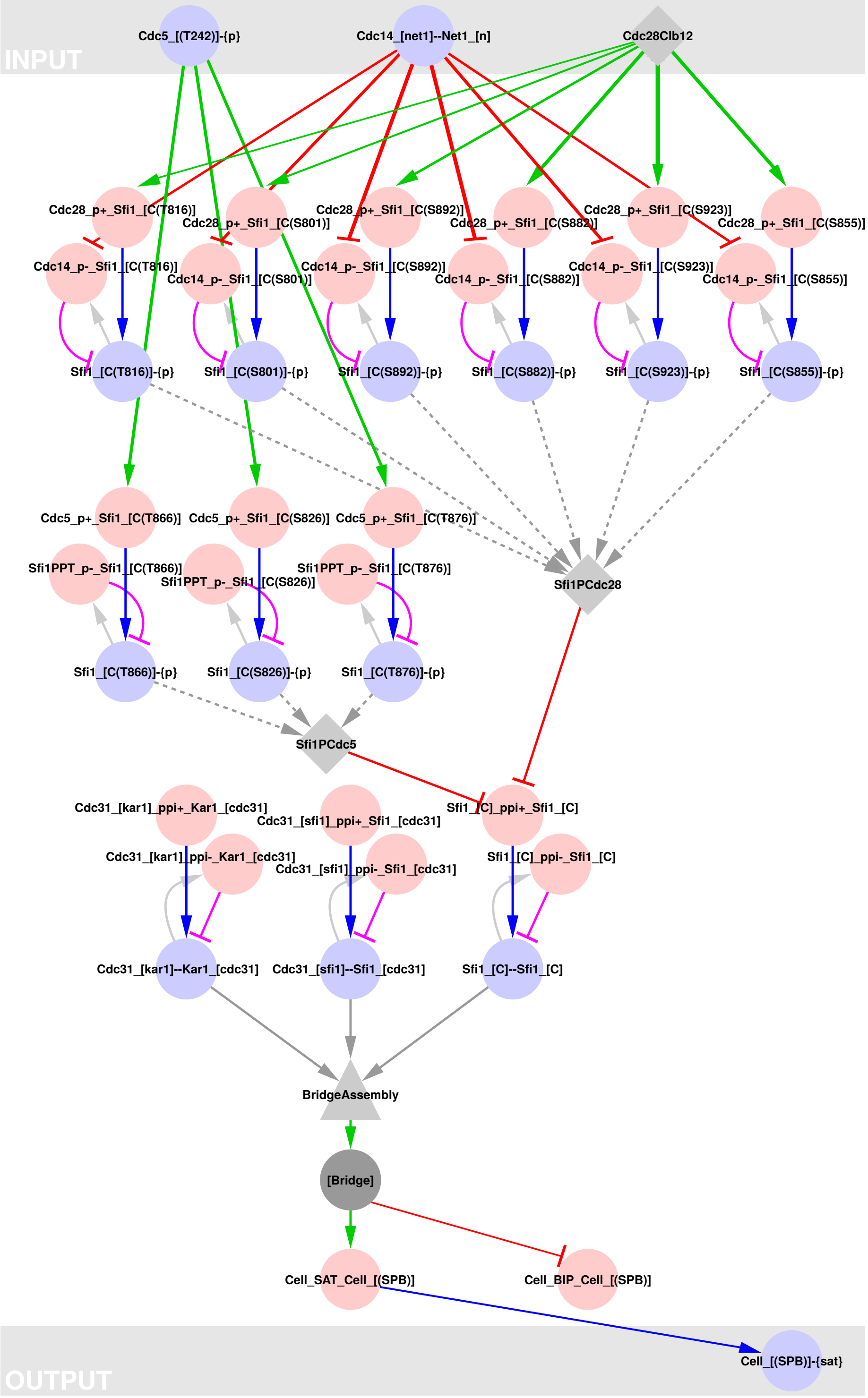

Supplementary Figure 19 *SPB satellite formation module.* At mitosis, SPB separation exposes half-bridges formed by Sfi1, Cdc31, and Kar1 molecules, where the C-termini of the Sfi1s are phosphorylated by Cdc5 and Cdc28-Clb1,2. Dephosphorylation of these exposed ends by Cdc14 at cytokinesis initiates a new round of SPB duplication. The bridge formation involves binding of a second set of Sfi1/Kar1/Cdc31 molecules. The bridge assembly primes the SPB to initiate satellite formation.

4.6.2 SPB duplication plaque formation module

Biology

Assembly of the second SPB continues with the formation of a satellite and duplication plaque on the initial Sfi1/Kar1/Cdc31 bridge. The satellite forms at the bridge by polymerisation of Spc42 on the free N–terminal of Sfi1 [140], which is regulated by Cdc28-Cln1/2/3 or Mps1-mediated phosphorylation of Spc42 [67]. Spc42 forms a crystalline structure that constitutes the central plaque in the SPB [17].

Furthermore, Spc42 recruits Cnm67 and Nud1, thereby expanding the satellite into the duplication plaque [45].

Implementation

The SPB duplication plaque formation module accounts for the regulation of the *SPB duplication plaque formation* reaction *Cell_DUP_Cell_[(SPB)]* (Supplementary Figure 20), which describes the formation of the duplication plaque, resulting in the production of the *Cell_[(SPB)]-{dup}* state. The transition from satellite to duplication plaque has no empirically identified regulatory mechanism. Hence, these processes are captured in a single step. Duplication plaque formation is represented by a series of protein-protein interactions. First, phosphorylated Spc42 binds the SPB bridge, where Spc42–Spc42 bonds leads to crystallisation of the central plaque, to which Cnm67 and Nud1 molecules are recruited.

The model accounts for transcriptional regulation of *SPC42* by MBF and turnover of Spc42 by degradation. However, there is no evidence for turnover of the Spc42 embedded in the central plaque. Hence, the model excludes degradation of molecules in the Spc42 crystal *Spc42Crystal*, and hence only turns over free Spc42.

The recruitment of duplication plaque components are the prerequisite for the *DuplicationPlaqueForm* node, which is required for the *Cell_DUP_Cell_[(SPB)]* reaction to execute, producing the state *Cell_[(SPB)]-{dup}*. This state represents the SPB with a duplication plaque, ready to mature into a complete SPB. Both SPBs are still connected to each other via the bridge.

Interfaces

The module depends on the expression and (de)phosphorylation of Spc42 by Cdc28-Cln1/2/3, Mps1 and Cdc14. However, the mechanisms of Mps1 regulation are unknown, leading to constant phosphorylation of Spc42. Hence, in practice, the module responds to the Spc42 expression and the existence of the bridge.

The output of the module is duplication plaque formation, represented by the state *Cell_[(SPB)]-{dup}*.

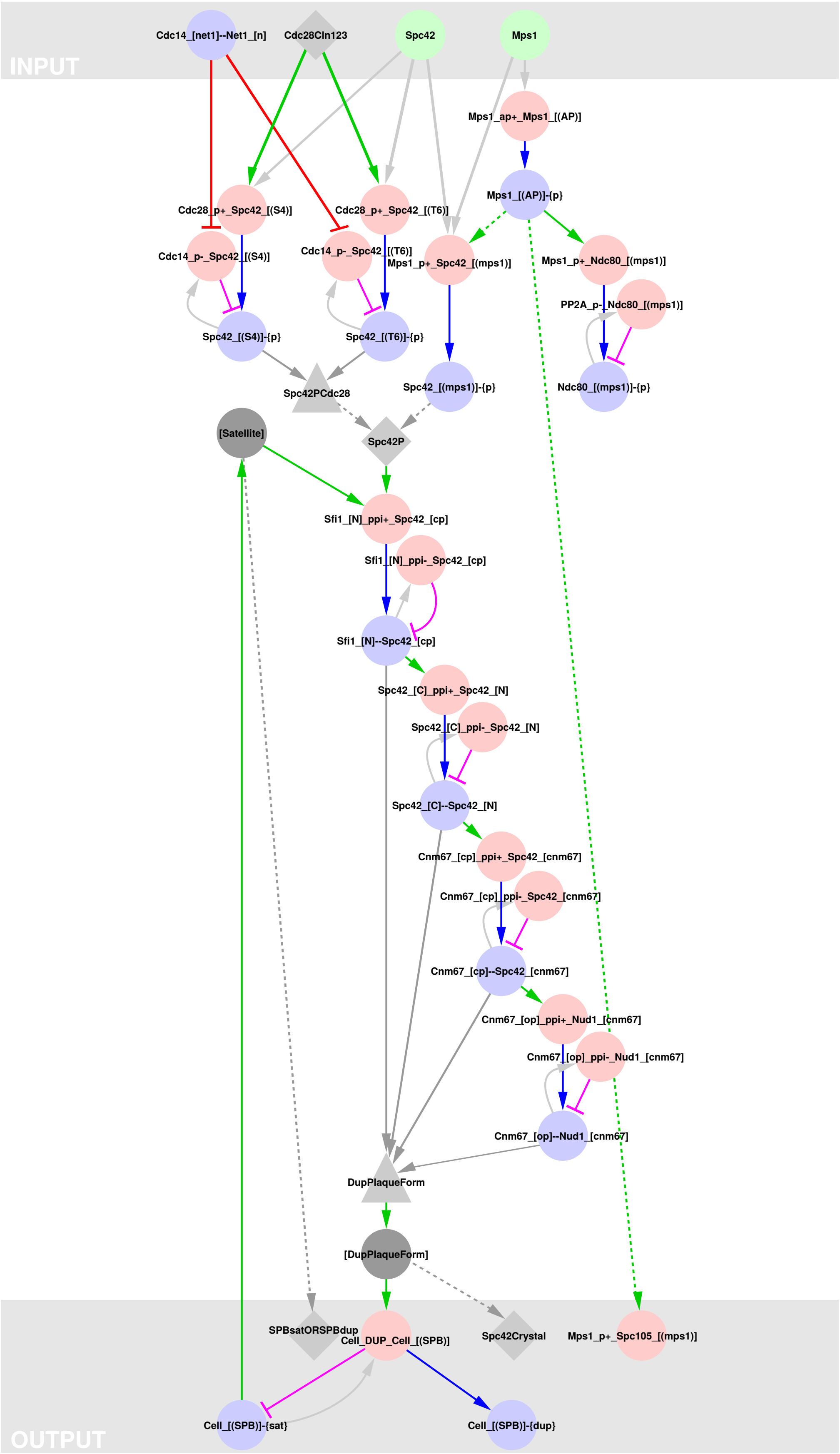

Supplementary Figure 20 *SPB duplication plaque formation module.* The dimerised Sfi1 with a free N-terminal recruits Spc42. Spc42 forms a crystalline structure, here simplified as a chain of Spc42 dimerisation events between the C- and N-termini of two Spc42 molecules. The dimerisation of Spc42 represents the crystallisation process leading to the formation of a duplication plaque which requires the recruitment of Cnm67 and Nud1. Satellite formation is regulated via Cdc28–Cln1/2/3 and Mps1 mediated phosphorylation.

4.6.3 SPB duplication module

Biology

The duplication plaque serves as a platform for the recruitment of the proteins forming the inner and outer plaques of the SPB. Spc72 is recruited to the outer plaque via Nud1 [174, 45]. Similarly, Spc29, Cmd1 and Spc110 are recruited to the inner plaque via Spc42 [174, 45].

Additionally, both the inner and outer plaques serve as a docking platform for the recruitment of the γ–tubulin complex, consisting of Spc97, Spc98 and Tub4 [174]. The γ–tubulin complex binds to the receptor proteins Spc72 at the outer plaque and Spc110 at the inner plaque [37, 174]. This complex serves as the platform for microtubule polymerisation.

Recruitment of the γ–tubulin complex to Spc110 is regulated by phosphorylation. It was demonstrated that Cdc28-Clb1/2/5/6 phosphorylation of Spc110 is important for its ability to recruit and activate the γ–tubulin complex [86].

Implementation

The SPB duplication module (Supplementary Figure 21) accounts for regulation of the *SPB duplication* reaction *Cell_SPB_Cell_J(SPB)]*. The *[DuplPlaque]* established in the previous step represents the duplication plaque, which is the prerequisite for the inner and outer plaque proteins to assemble into mature plaques. Plaque formation occurs via unregulated assembly of the γ–tubulin complex, which is accounted for with the subunits Spc97, Spc98 and Tub4, combined in the node *yTubSmallComp*. The γ–tubulin complex serves as a prerequisite for the formation of the inner and outer plaques.

The model accounts for recruitment of the inner plaque subunits Spc110, Spc29, and Cmd1, combined in the node *[InnerPlaque]*. The model also accounts for the positive influence of Spc100 phosphorylation by Cdc28-Clb1/2/5/6, combined in the node *Spc110Phos*, on Spc98 and Spc110 association.

The model accounts for recruitment of the outer plaque subunits Nud1 and Spc72.

Upon successful formation of the inner and outer plaques, combined in the node *[PlaqueForm]*, the *SPB duplication* reaction can execute, producing the state *Cell_J(SPB)]-{spb}*. This state represents the two SPBs, which are still connected to each other via the bridge.

Interfaces

The module requires the prior formation of the duplication plaque and responds to Cdc28-Clb1/2/5/6 and Cdc14-mediated (de)phosphorylation of Spc110.

The output of the model is the formation is the completion of SPB duplication (but not separation), represented by the state *Cell_J(SPB)]-{spb}*.

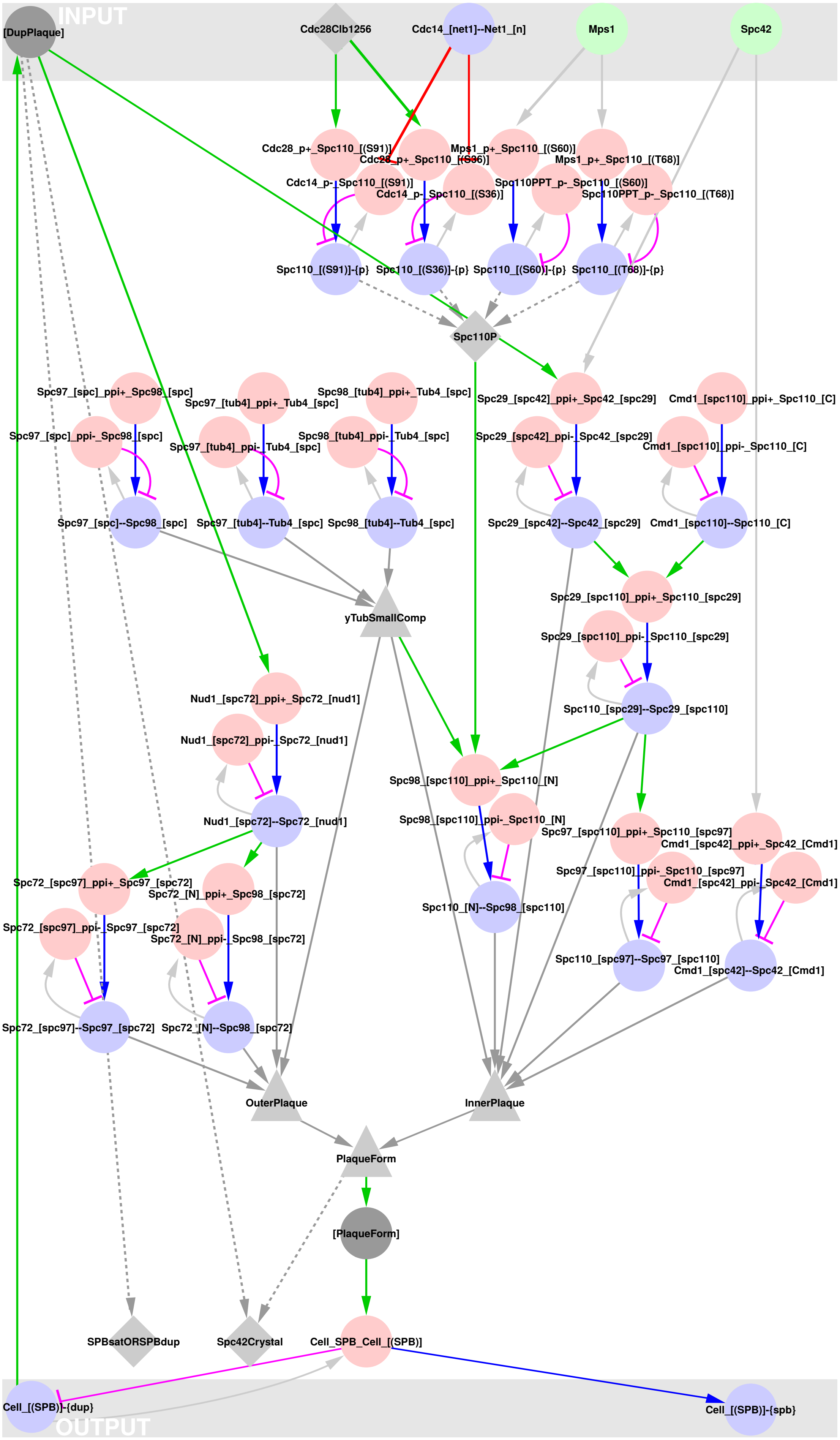

Supplementary Figure 21 *SPB duplication module.* During duplication plaque formation, proteins are recruited to the central plaque, forming the outer and inner plaques. Simultaneously, the growing second SPB is inserted into the nuclear pore and matures. The two SPBs remain connected to each other via the bridge.

4.6.4 SPB separation module

In the next step, the two SPBs must be separated and pushed away from each other for spindle elongation. The separation of the two mature SPBs requires disruption of the connecting bridge through phosphorylation of Sfi1 by Cdc28-Clb1,2 and/or Cdc5 [67, 174]. Additionally, pulling forces exerted by microtubules seem to contribute to SPB separation. However, according to [174], the two SPBs can separate without the involvement of microtubules.

The microtubules are formed by tubulin dimers. These dimers consisting of either Tub1-Tub2 or Tub2-Tub3 polymerise from Tub4 in the γ–tubulin complexes attached to the inner and outer plaques. This polymerisation is a highly dynamic process with polymerisation and depolymerisation occurring frequently.

Nuclear microtubules consist of spindle microtubules, which connect SPBs with centromeres, and of interpolar microtubules. The latter form an antiparallel bundle that overlaps in the so called midzone [143]. The protein Ase1 crosslinks these interpolar microtubules [145]. Cdc28-Clb1/2 phosphorylates Tub4 [72]. This phosphorylation has a negative influence on the formation of interpolar microtubules [106]. The formation of interpolar microtubules and their continuing polarisation is one force pushing the two SPBs away from each other. In addition to this mechanism, the kinesin motor proteins Cin8 and Kip1 bind to the midzone [44, 56, 143] where they can actively push the two SPBs away from each other. The relative contribution of these mechanisms is not clear [143].

The two SPBs continue their separation and spindle elongation with one SPB being pushed or pulled towards the daughter. In addition to nuclear microtubules, the astral microtubules contribute to this process. The astral microtubules attach to the cell cortex via Bim1 and Kar9, where Bim1 binds to the plus ends of the microtubules [93], and also recruits Kar9 [36]. This interaction requires phosphorylation of Kar9 by Cdc28-Clb5/6 [99] or Cdc28-Clb1/2 (hypothesised in the gap-filling process). Kar9 in turn binds to Myo2, which is connected to actin cables via Act1 [181, 87]. Astral microtubules connected to Bim1 and the actin cables connect the SPBs to the cell cortex and help position the spindle and nucleus. The Cin8 tetramer interacts with tubulin [56, 135].

Spindle formation can be disrupted by nocodazole treatment. Nocodazole inhibits the polymerisation of microtubules.

Implementation

The SPB separation module (Supplementary Figure 22) accounts for SPB separation, represented by the *SPB bipolar initiation* reaction *Cell_BIP_Cell_[(SPB)]*, producing the *Cell_[(SPB)]-{bipolar}* state. The model distinguishes between microtubules emanating from the outer plaques of the SPBs, forming astral microtubules (node *AstralMTPol*), and microtubules emanating from the inner plaques of the SPBs, the nuclear microtubules (node *NucMTPol*). This distinction allows to discriminate between the functions which the different microtubules are involved in.

The nuclear microtubules form, together with the microtubule crosslinking machinery (Ase1, Tub1 and Tub4, node *CosslinkedInterpolarMT*), and the kinesin motors (Kip1, and Cin8 tetramer (*Cin8HD*), node *KinesinMotors*), the nuclear pushing forces (node *NuclearForce*).

The astral microtubules form, together with the actin motor proteins Kar9 and Myo2 (node *ActinMotor*), the cytoplasmic forces (node *CytoplasmicForce*).

The nuclear and cytoplasmic forces together form the microtubular forces which push and pull the two SPBs apart, combined in the node *[MTForces]*, leading to the executing of the *SPB bipolar initiation* reaction.

The model represents microtubule polymerisation as the simultaneous existence of Tub1–Tub2 or Tub2–Tub3 bonds with the Tub1–Tub4 or Tub3–Tub4 bonds, indicating polymerisation from the γ–tubulin complexes (node *TubulinDimer*). The rxncon language cannot meaningfully describe the dynamics of (de)polymerisation and hence, the model does not account for these dynamics.

The model accounts for nocodazole inhibition by interrupting the dimerisation between Tub1 and Tub2, and Tub2 and Tub3, respectively.

Interfaces

The module depends on the duplication of the SPB (existence of a *[DaughterSPB]*), phosphorylation of Tub4 (by Cdc28_Clb1/2) and Kar9 (Cdc28–Clb1/2/5/6) to stabilise the spindle, and actin cables to generate cytoplasmic forces together with the astral microtubules. The separation of the SPBs and hence, the generation of the bipolar spindle requires that the bridge between the two SPBs is disintegrated.

The output of the module is the microtubule forces (*[MTForces]*) needed to create a bipolar spindle with tension (node *[TensionInitiation]*), and the cytoplasmic force needed to position the spindle with one pole in each of the mother and daughter cells (node *[SpindlePositioning]*).

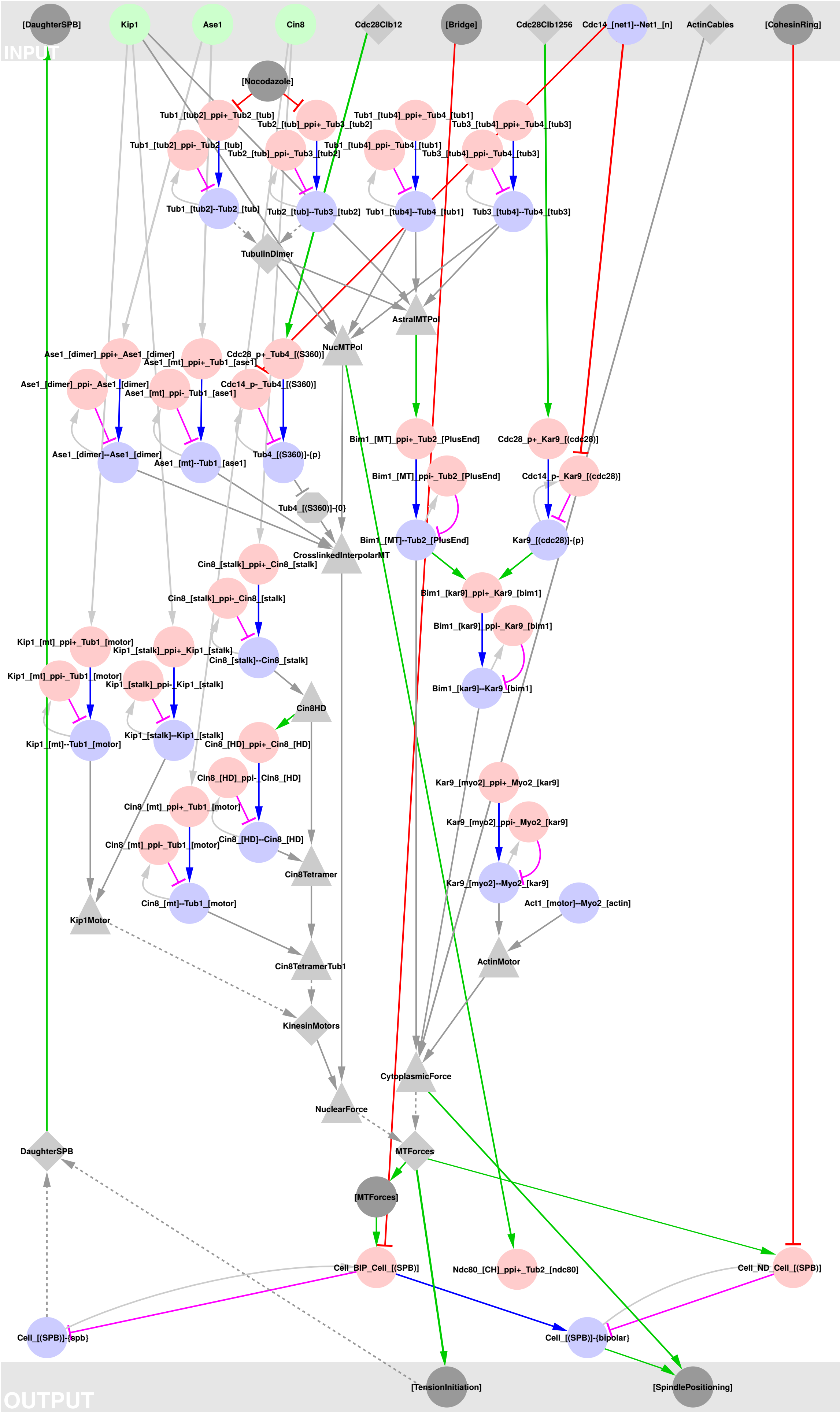

Supplementary Figure 22 *SPB separation module.* The two SPBs are separated by pushing and pulling forces, accomplished by interpolar microtubules and astral microtubules.

4.6.5 SPB spindle tension module

Biology

The bipolar spindle is the prerequisite for separation of the chromosomes. Bipolar attachment of nuclear microtubules attached to sister kinetochores triggers chromosome segregation and partitioning. The kinetochore is a protein structure consisting of several complexes attaching to the centromeric region of the DNA. The outer part of the kinetochore allows attachment to microtubules. Correct microtubule–kinetochore attachment requires that microtubules emanating from the two opposite SPBs attach to the same sister chromatid pair. This type of interaction is referred to as biorientation or amphitelic attachment [16]. This mechanism is tension sensitive. Tension sensing acts on the balance between phosphorylation and dephosphorylation of substrates involved in stabilisation of microtubule-kinetochore attachments. Unless amphitelic attachment is accomplished, tension-sensitive aurora kinase Ipl1 phosphorylates several subunits of the kinetochore, thereby destabilising microtubule-kinetochore attachment. Upon amphitelic attachment, the interaction between the microtubules and kinetochores exerts tension at the sister chromatids, leading to the deactivation of Ipl1, followed by dephosphorylation of its substrates. Ipl1 is part of the chromosomal passenger complex (CPC), which contains the subunits Nbl1, Sli15, Ipl1, Bir1 [103]. Tension establishment furthermore requires the condensin and cohesin protein complexes [16, 94]

The CPC substrate Dam1 is phosphorylated at several residues by Ipl1: S20, S64, S257 and S292 [21]. These Dam1 phosphorylations, together with Spc34 phosphorylation at T199 indicate low tension, thereby destabilising the interaction between Dam1 and the microtubule subunits Tub1 and Tub2 [21]. Furthermore, the C-terminal phosphorylation of Dam1 inhibits the interaction between Dam1 and Ndc80 [73].

The microtubule-kinetochore attachments and bioriented chromosomes are stabilised when the spindle is correctly assembled. At this stage, the cell can commit to segregate the chromosomes and partition them between the mother and daughter cell. In other words, the cell passes the spindle assembly checkpoint (SAC). SAC then initiates the release of Cdc20, a prerequisite for APC/C-Cdc20 activation.

Before passing SAC, Cdc20 is part of a kinetochore complex including Bub1, Bub3, Mad1, Mad2 and Ndc80. This complex allows Mad2 dimerisation [187], which is a prerequisite for the interaction between Cdc20 and Mad20, which traps Cdc20 at the kinetochores and to inhibit its interaction with the APC/C [16]. This complex assembles in the absence of stable kinetochore-microtubule attachment. Complex assembly is initiated by Mps1 interaction with Ndc80, allowing Mps1 to phosphorylate Spc105 [16]. Phosphorylated Spc105 recruits Bub1, Bub3, Mad1 and Mad2 [16]. This complex disassembles upon biorientation, initiated by the dissociation between Mps1 and Ndc80, which leads to the deactivation of Spc105 and its ability to interact with the remaining subunits. Cdc20 release allows it to interact with the APC/C [16], initiating a wave of degradation and eventually anaphase (Supplementary Figure 11).

Implementation

The SPB spindle tension module (Supplementary Figure 23) accounts for the integration of the molecular events required to stabilise tension at the chromosomes, representing correct kinetochore-microtubule attachment. The model also accounts for the Dam1, Ndc80 and CPC complexes, which are involved in tension stabilisation.

The model accounts for the two kinetochore complexes Ndc80 and Dam1. The model includes the Ndc80 complex subunits Spc24, Spc25, Nuf2 and Ndc80 [16], combined in the *Ndc80Comp* node. The Dam1 complex consists of the subunits Ask1, Dad4, Dam1, Duo1 and Spc34 [83], as accounted for and combined in the *Dam1Comp* node. Both complexes interact with each other via Dam1–Ndc80, Ask1–Ndc80, and Ndc80–Spc34 [73]. These protein complex interactions are accounted for and combined in the node *Ndc80Dam1Recruitment*.

The model accounts for the CPC. The CPC must be located in proximity to its substrates at the centromeres, accounted for by the requirement of Bir1 being bound to the kinetochore protein Cbf2 [182], which in turn is attached to centromeric regions of the DNA [16]. These conditions together with CPC (subunits Bir1, Nbl1, Ipl1, Sli15 forming the node *CDC*) resemble the active Ipl1 kinase complex which can reach its substrates, combined in the node *AuroraBActive*. Ipl1 phosphorylates Ndc80, subunit of the Ndc80 complex, and the Dam1 complex subunits Spc34 and Dam1 [21].

The model accounts for stable kinetochore-microtubule attachment in two steps: First, tension is initiated (node *[TensionInitiation]*). This requires the input from several modules: a bipolar spindle and microtubule forces (nodes *[BipolarSpindle]* and *MTForces* from Section 4.6.4), replicated DNA (node *[DNAReplicated]* from Section 4.5.5), an intact cohesin ring (node *[CohesinRing]* from Section 4.5.6), and condensin bound to the centromeres (node *CondensinAtCEN* from Section 4.8.1). The node *[TensionInitiation]* represents correct kinetochore-microtubule attachment, accounting for tension at the centromeres. The initially generated tension is inhibitory towards Ipl1 activity [88]. Now, the activity of Glc7, the Ipl1 antagonist, dominates, resulting in the dephosphorylation of Ipl1 substrates [43]. The activity of Glc7 is positively influenced by its interaction with Spc105 [136]. Ipl1 deactivation leads to a shift in phosphorylation of its substrates, of which Spc34, Dam1 and Ndc80 are accounted for.

Second, the wave of dephosphorylation allows the stabilisation of Dam1 complex interaction with microtubules, thereby strengthening kinetochore-microtubule attachment. Dephosphorylation of Dam1 allows its interaction with the tubulins Tub1 and Tub3 [21], combined in the node *Dam1MT*. Duo1 as Dam1 complex subunit is allowed to bind to tubulins Tub1, Tub2 and Tub3 [97], combined in the node *Duo1MT*. It is hypothesised that Duo1–microtubule attachment also requires kinetochore interaction via Ndc80 [83]. Ndc80 as part of the Ndc80 complex binds to Tub2. This interaction represents amphitelic attachment and requires that Ndc80 is unphosphorylated on residue S100 [178].

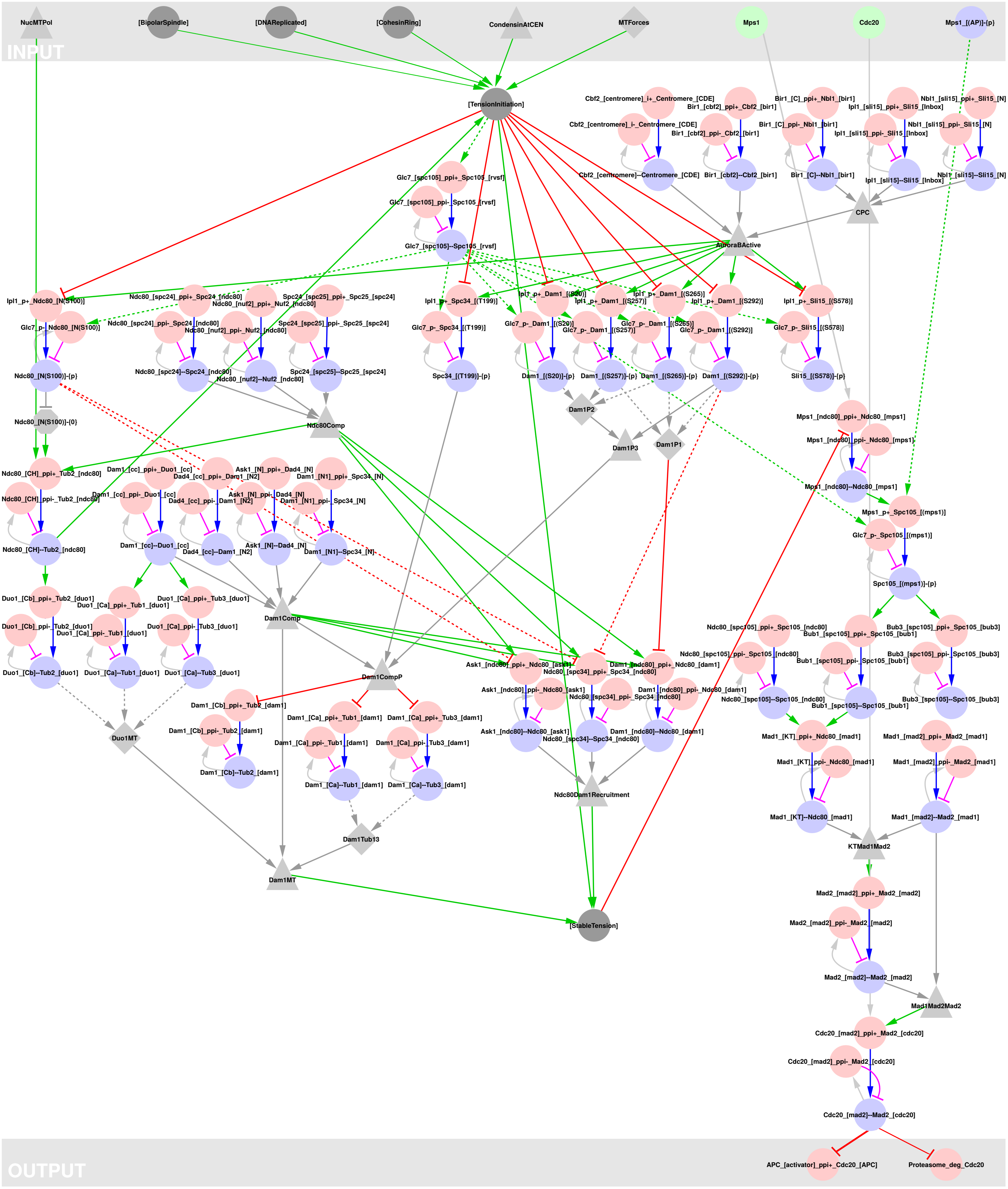

Supplementary Figure 23 *SPB spindle tension module.* In this step, the microtubules emanating from the SPB attach to the kinetochores which have formed around the sister chromatids. For each kinetochore, the microtubules attaching must be from opposite poles, and only upon correct attachment, tension is exerted, leading to the deactivation of Ipl1 and stabilising the bond between Ndc80 and Tub2, thereby reinforcing stable tension.

Dam1MT indicates the stabilisation of the interaction between the Dam1 complex and microtubules and, together with interaction between the Dam1 and Ndc80 complexes, hence, the formation of stable tension. Lastly, *[StableTension]* also requires the tension generating factors and hence, the node *[TensionInitiation]*.

Cdc20 release by SAC as implemented in the model is accomplished by a signalling cascade starting with the inhibition of the interaction between Mps1 and Ndc80 requiring *[StableTension]*.

Interfaces

The spindle tension module requires a correctly formed spindle, i.e. Bipolar spindle, microtubule force generation, and replicated DNA, that is held together by condensin and cohesin rings.

The output of the module is a stabilised spindle with tension that leads to a signal releasing Cdc20 and triggering the activation of APC/C.

4.7 Cell division

This part describes the morphological changes of the yeast cell during the CDC. This process is divided into three steps: First, the establishment of a bud site and bud emergence (Section 4.7.1), second, bud growth (Section 4.7.2), and third, the signalling of correct bud morphology to other parts of the network (Section 4.7.3). Lastly, this part also describes how these morphological changes allow for the separation of mother and daughter cell, resulting in cytokinesis (Section 4.7.4).

4.7.1 Bud emergence module

Biology

Bud emergence starts with membrane polarisation and assembly of the bud neck. Membrane polarisation requires local accumulation and activation of the GTPase Cdc42 [15]. Several hypotheses have been proposed to explain polarised Cdc42 accumulation and activation, as the exact mechanism is not entirely clear [15, 59]. In brief, the two major hypotheses rely on positive feedback mechanisms and explain membrane polarisation with either Cdc42 accumulation via landmark proteins, or an actin-cable based transport system, which sequesters Cdc42 to a spontaneously formed cluster.

Here, we focus on polarised accumulation and activation of Cdc42 via landmark proteins. This is thought to be accomplished by bringing Cdc42 in proximity with its GEF Cdc24, and the GAPs Bem2, Bem3, Rga1 and Rga2. In this view, it is assumed that Cdc24 becomes locally active, whereas the deactivating GTPase activating proteins (GAPs) Bem2, Bem3, Rga1 and Rga2 are equally distributed. This enables localised guanosine diphosphate/guanosine triphosphate (GDP/GTP) cycling of Cdc42, and hence, enables membrane polarisation.

Cdc24 localisation is accomplished by landmark proteins and Rsr1. Depending on the cell type, different landmark proteins are involved, resulting in different polarisation patterns. Haploid cells have an axial polarisation pattern, whereas diploid cells have a bipolar one [15]. However, the mechanistic role of the landmark proteins remains unknown [70]. Rsr1 is recruited to landmark proteins via its GTPase Bud5 [59].

Bud5 interaction with the landmark proteins stimulates Rsr1 activation, which in turn enhances Rsr1 interaction with Cdc42 and Cdc24. However, the cell is able to polarise Cdc42 in the absence of spatial markers, requiring Bem1. The interaction between Bem1 and Cdc24 is thought to depend on Cdc28-Cln1/2/3-mediated phosphorylation of Bem1 [15].

Localised Cdc42 activation enables assembly of a septin ring at the polarisation site. Recruitment and assembly of the septin ring subunits depends on Cla4 and Cdc42. First, Cla4 is activated by autophosphorylation [166], which is supposedly supported by its interaction with active Cdc42. Active Cla4 can now phosphorylate Cdc3 and Cdc10 [116, 166]. Cla4 is furthermore thought to be recruited to the cell membrane via its interaction with phosphatidylinositol (PI) 4-phosphate [173].

Phosphorylation of Cdc3 and Cdc10 enables them to interact with each other and with Cdc12. The Cdc3–Cdc12, Cdc10–Cdc12 and Cdc11-Cdc12 dimers are required to establish the septin ring [14]. The exact topology of the septin ring, however, is not entirely resolved. Recruitment of septin ring proteins to the site of polarisation was shown to require Gic1 and Gic2 [66].

Polarisation establishment is furthermore sensitive to pheromone treatment. Pheromone exposure triggers the mating signalling pathway, stabilising the Far1–Cdc24 interaction and Far1 dependent nuclear sequestration of Cdc24, thereby inhibiting Cdc24 interaction with Bem1 [18] and Rsr1 [36]. This ultimately inhibits the cell to establish a bud site.

Implementation

The bud emergence module (Supplementary Figure 25) accounts for local activation of Cdc42 and the assembly of the bud neck. The model accounts for local activation of Cdc42 by a mechanism involving landmark proteins. The model only accounts for a generic landmark protein, *LM*, as their mechanistic role in polarisation remains unknown [70]. The landmark protein interacts with the GEF Bud5, activating Rsr1 when in proximity to Bud5. This in turn is the prerequisite for Cdc24 and Cdc42 recruitment, forming a localised complex (node *Cdc42Rsr1Loc*). It is assumed that the landmark proteins are localised to a single site in the cell membrane. Bem1, however, has no distinct localisation pattern, and hence, the model cannot explain the establishment of a single polarisation site in the absence of Rsr1. Additionally, Bem1 forms two different complexes with Cdc42 and Cdc24, one with Ste20 and one with Cla4. In the absence of Rsr1, either of these two complexes is thought to establish a site of polarisation [59]. Cdc24 and Cdc42 bound to Bem1 (node *Cdc42Bem1Loc*), is thought to locally activate Cdc42, combined in the node *Cdc42LocAct*, which is a requirement for the GEF reaction of Cdc42 by Cdc24. This reaction is furthermore supported by Cdc24 and Cdc42 in complex with other proteins, such as Bem1 and Ste20 (node *Bem1Cdc24Ste20Cdc42*), as well as Bem1 and Cla4 (node *Bem1Cdc24Cla4Cdc42*). These two complexes are implemented as the requirement for membrane polarisation, represented by the node *[SymmetryBreakingBud]*. Symmetry breaking is the prerequisite for apical growth, represented by the node *[ApicalGrowth]*. Active Cdc42 together with symmetry breaking initiates the assembly of the septin ring. Here, the septin ring components Cdc3, Cdc10, Cdc11, Cdc12, as well as Gic1 and Gic2 are accounted for. The additional septin ring component Shs1 is omitted in the model due to its redundant role. The assembly of the septin ring at the site of polarisation is represented by the node *[SeptinPol]*, which is the prerequisite for the macroscopic *Bud emergence* reaction *Cell_EM_Cell_[(bud)]* to execute, producing the state *Cell_[(bud)]–{small}*.

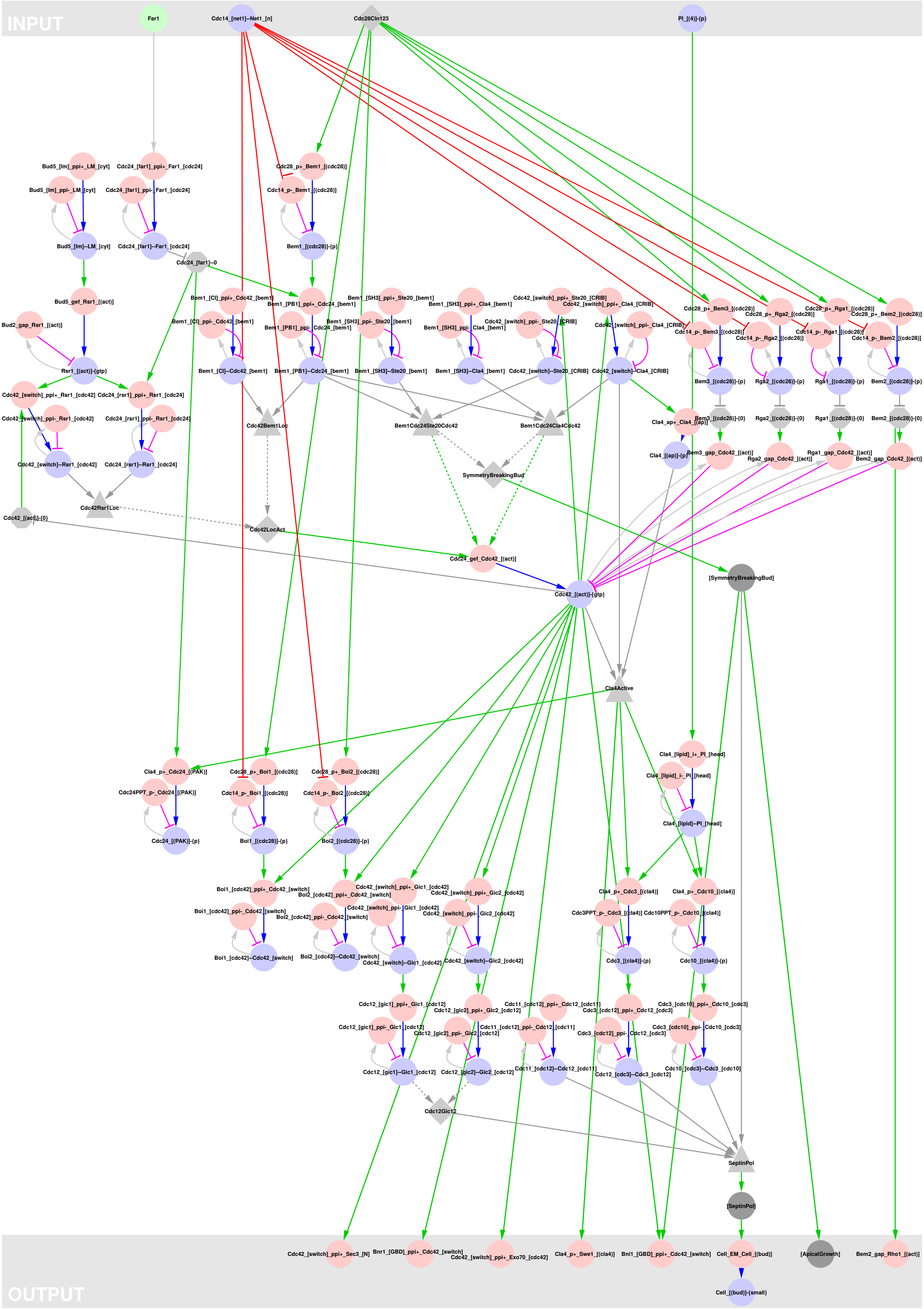

Supplementary Figure 24 *Bud emergence module.* Bud emergence requires the local accumulation and activation of Cdc42, leading to the symmetry breaking and polarisation of the cell membrane. At the same time, septin ring proteins required for bud neck establishment are recruited to the site of polarisation.

Interfaces

The bud emergence module depends on Cdc28-Cln1/2/3 mediated phosphorylation of Bem1, expression of Far1 and its binding to (and nuclear sequestration of) Cdc24, and the presence of PI4P.

The outputs of the module are symmetry breaking, septin polymerisation and apical growth, as well as the activation of a number of Cdc42 targets involved in polarity and secretion.

4.7.2 Bud growth module

Biology

Bud growth in *S. cerevisiae* is initially polarised, requiring that the material and enzymes needed to enlarge the daughter cell are targeted to the tip of the growing bud. The material is transported in vesicles to the plasma membrane, where the vesicles dock and fuse with the plasma membrane, accomplished by polarised exocytosis. Polarised exocytosis requires the localisation and activation of the exocyst complex at the site of polarisation to stimulate vesicle fusion [60]. Actin cables mediate polarised exocytosis. It was shown that Exo70 interacts with Cdc42-GTP [175], and the same study suggested that also Sec3 interacts with Cdc42 and Rho1 [49].

In addition to localisation through Sec3, exocytosis requires both the establishment of the polarisome and polymerisation of actin cables, which requires Cdc42-GTP and/or Rho1-GTP. Polarised secretion leads to cell wall integrity (CWI) signalling [185], which activates Rho1, stabilising the polarised secretion until the apical to isotropic switch.

Rho1 is activated upon membrane targeting by bringing it together with its GEF Rom2 [112, 138], which is localised to the membrane by interaction with PI 4,5-bisphosphate PI(4,5)P_2 [7]. PI phosphorylation is regulated via the kinase-phosphatase pairs Sst4/Mss4 and Sac1/Inp51 [184, 155, 42, 7]. Either of two CWI pathway sensors, Slg1 or Mid2, additionally interact with Rom2, thereby enhancing the activity of Rom2 [119]. Furthermore, Rho1 can be activated by Tus1 [142], another Rho1 GEF. Tus1 activation requires phosphorylation by Cdc5 or Cdc28-Cln1/2/3 [183, 78], and is likely the driving force in the repolarisation of the secretory machinery towards the bud neck during cytokinesis. Rho1 also activates the glucan synthesis complex at the site of polarised growth by binding to the subunits Fks2 and Gsc2. Either of these Rho1-glucan synthase complexes is required for glucan synthesis activity [124, 15].

Polarised bud growth requires actin cable mediated exocytosis. Actin cables orient towards the site of polarisation in a manner dependent on the formins Bni1 and Bnr1 [39]. The formins are required for actin cable orientation and nucleation, where Bni1 nucleates actin cables in the daughter cell at the bud tip, Bnr1 nucleates actin cables in the mother cell at the bud neck [15]. Bni1 was shown to interact intramolecularly with its C-terminal diaphanous autoregulatory domain (DAD) and GTP binding domain [3, 33]. This intramolecular interaction inhibits Bni1 activity. This autoinhibition is relieved by binding

of Rho1 or Cdc42 to Bni1 [76, 38], as discussed by [15]. Bni1 can then to interact with the polarisome via Spa2. The polarisome contains two more subunits, Bud6 and Pea2, both of which bind to the scaffold protein Spa2. The polarisome interacts via Spa2 with either Msb3 or Msb4, which are GAPs of Sec4.Together with the Sec4 GEF Sec2, the polarisome allows for vesicle tethering via active Sec4 [64], which is the prerequisite for active exocytosis at the site of polarisation.

Treatment with Latrunculin A (LatA) disrupts actin cable polymerisation. Hence, LatA inhibits polarised growth of the bud.

Implementation

The bud growth module (Supplementary Figure 25) accounts for the molecular mechanism of exocytosis at the polarised bud site. The model accounts for polarised growth in the following way: First, the exocyst complex is localised to the polarisation site through Sec3 interaction with Rho1-GTP or Cdc42-GTP. This requires Rho1 activation at the site of polarisation via active Cdc42 combined in the node *Rho1Activation*. The model accounts for the exocyst complex subunits Sec3 and Exo70; the remaining subunits of the exocyst are omitted. Second, the model accounts for exocyst membrane targeting via interaction of the exocyst subunits Exo70 and Sec3 with the membrane localised PI(4,5)P_2 [60, 52] (node *Sec3PM*). The model accounts for the polarisome subunit Pea2, Spa2, Bud6, Bni1 (node *Polarisome*), which interacts with either of Msb3 or Msb4 (nodes *PolarisomeMsb3* and *PolarisomeMsb4*).

In the model, it is hypothesised that Bni1 and Bnr1 interactions with Cdc42 or Rho1 require *[SymmetryBreaking]*, as discussed in [59], allowing polarised growth at the bud tip. We hypothesise that the Bni1 autoinhibitory mechanism also applies to Bnr1 ([3, 33], and structural similarities between Bni1 and Bnr1 [33]. Similarly, we account for Bnr1 relief of autoinhibition by Cdc42 or Rho1 binding, a model hypothesis based on [76] and [38] due to similarity between Bni1 and Bnr1. The actin cables (*BudTipCables* and *BudNeckCables*, combined in the node *ActinCables*) can be polymerised from either Bni1 or Bnr1 [101], which can compensate for each other [65].

The interactions between Cdc42 and Rho1 and the exocyst subunit Sec3, in combination with its docking to the membrane and polarisome, and actin cable polymerisation enables exocytosis [1, 138, 52] in the model (node *[Exocytosis]*).

The model implicitly describes the process of vesicle tethering. Exocytosis is accomplished by the combination of Sec3 (exocyst) plasma membrane localisation, Sec4-GDP, and actin cables. Actin cable polymerisation from the bud tip requires the dimerisation of Bni1 at its formin homology 2 (FH) domain [176], which allows it to nucleate actin cables [123].

Exocytosis is implemented as a prerequisite for *[ApicalGrowth]*, which also requires active Cdc42. The model also accounts for the activation of glucan synthesis, by the Rho1-mediated activation of Fks1 and Gsc1, combined in the node *[GlucanSynthesis]*. Apical growth together with active glucan synthesis enables the *Bud growth* reaction *Cell_Growth_Cell_[(bud)]* to trigger and activate the state *Cell_[(bud)]-{large}*.

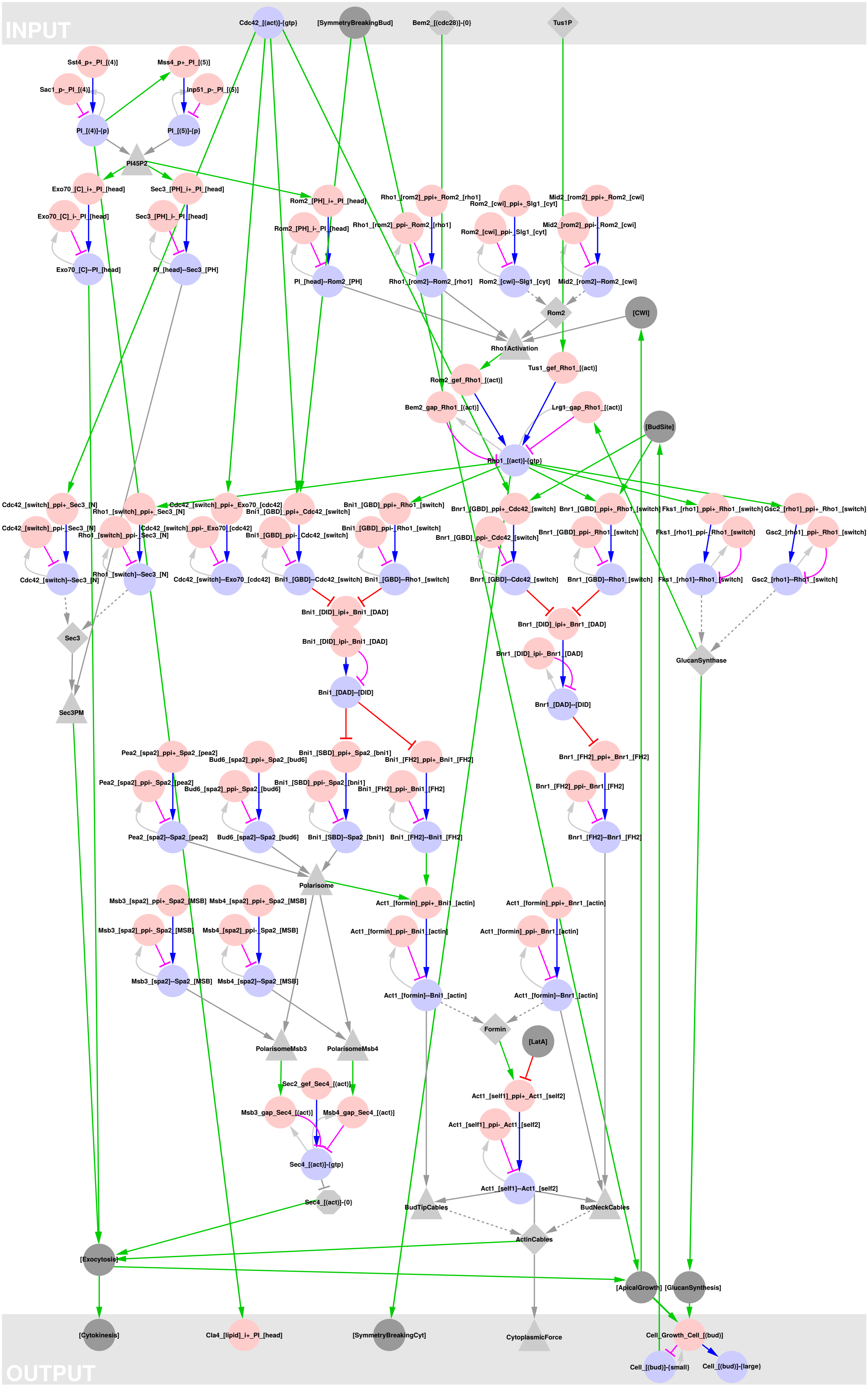

Supplementary Figure 25 *Bud growth module.* Cell membrane polarisation and recruitment of bud neck proteins leads to the formation of a bud. Polarised bud growth is enabled via actin cable mediated exocytosis.

Interfaces

The bud growth module depends on active Cdc42, Symmetry breaking and Tus1 phosphorylation.

The outputs of the module are apical growth and glucan synthesis, which are required for bud growth, and exocytosis, which is required for both apical growth and cytokinesis.

4.7.3 The bud morphology checkpoint module

Biology

Growth of the bud is required to overcome the morphogenesis checkpoint and to downregulate Swe1. Swe1 phosphorylates and inhibits Cdc28-Clb1/2 [71], delaying mitotic entry until bud size and/or morphology supports cell division. Swe1 phosphorylations are antagonised by the phosphatase Mih1 [59]. In the unperturbed cell cycle, the role of Swe1 seems to be minor. However, its role becomes more distinct and important when the cell is exposed to stresses (as discussed by [59]).

Implementation

The bud morphology checkpoint module (Supplementary Figure 26) accounts for Swe1 regulation in the following way: First, Hsl1 and Hsl7 are recruited to the bud neck via Cdc3 [59]. This recruitment depends on the input node *[LargeBud]*, capturing the currently unknown mechanism of size and/or morphology sensing. This allows Swe1 recruitment to the bud neck via Hsl7 and subsequent Cdc28-Clb1/2 mediated phosphorylation of Swe1 [82, 36, 59]. The Swe1 interaction with Hsl7 also primes Swe1 for Cdc5-mediated phosphorylation [6]. The CDK-mediated phosphorylation of Swe1 is thought to have a positive influence on Cdc5 phosphorylation [59]. Phosphorylation of Swe1 by Cdc5 or Cdc28 primes it for degradation [59], combined in the node *Swe1degP*. Phosphorylated Swe1 enables Met30-SCF mediated ubiquitylation and subsequent degradation (Section 4.3.1).

Interfaces

The module depends on the presence of a bud, Cdc28-Clb1/2/5/6 activity and Cdc5 activity. The output of the model is Swe1 degradation, which removes one of the checks on mitotic entry.

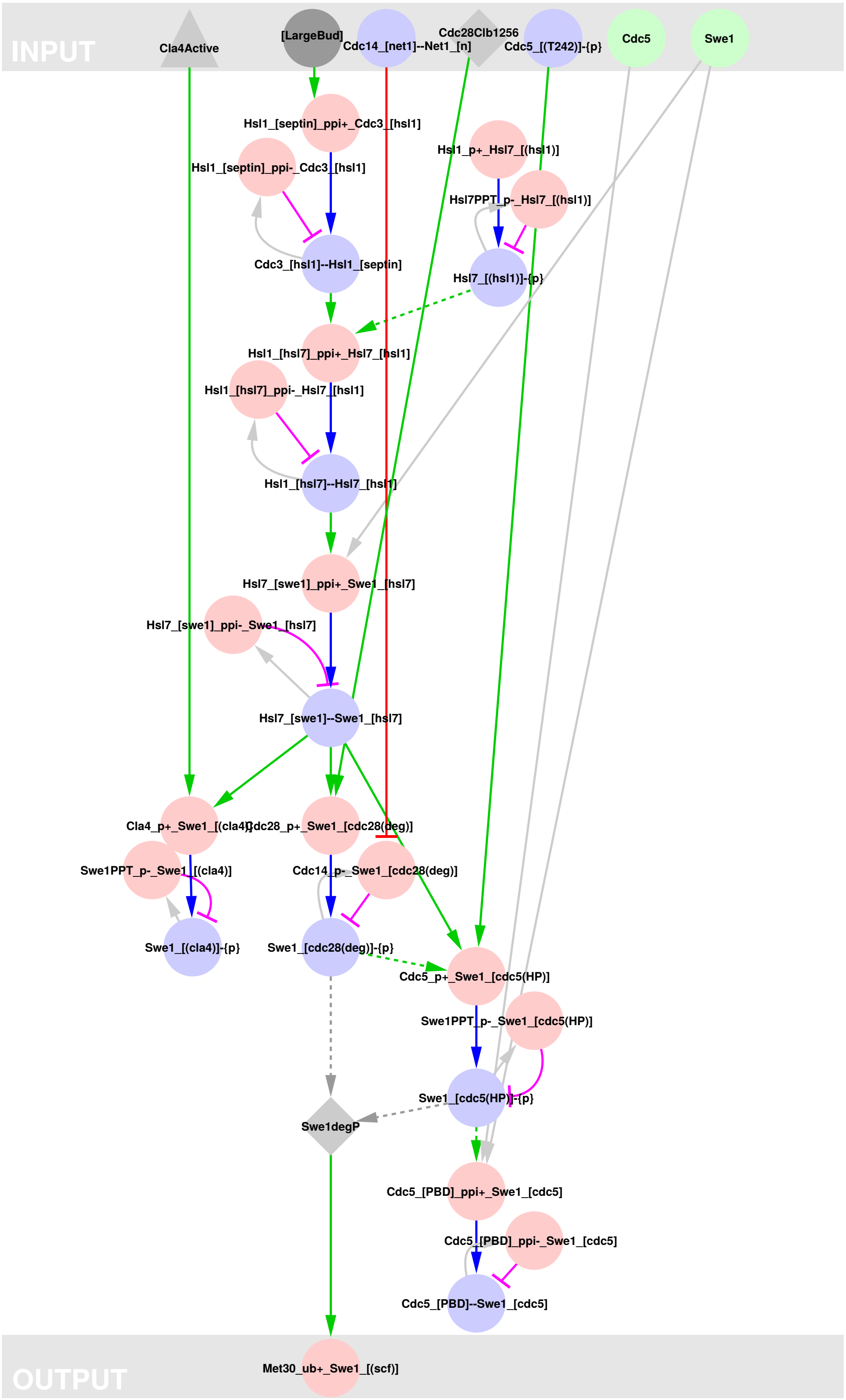

Supplementary Figure 26 *Bud morphology checkpoint module.* The neck of a mature bud recruits the septins Hsl1 and Hsl7, a prerequisite for Swe1 recruitment. Cdc28-Clb1/2 and Cdc5 phosphorylate bud neck localised Swe1 and prime it for degradation.

4.7.4 The cytokinesis module

Biology

The final step of the CDC is cytokinesis. Entry into cytokinesis requires the deactivation of the spindle orientation checkpoint (SPOC), which remains active until the daughter SPB enters the daughter cell. Upon passing SPOC, the mitotic exit network (MEN) is initiated, leading to the activation of the kinase Dbf2. Dbf2 activates Cdc14 (Supplementary Figure 14), and the chitinase Chs2.

MEN activation occurs via Tem1 and Cdc15. Tem1 is a GTPase which stimulates its own activity [47]. Tem1 in complex with Nud1 and Cdc15 activates Cdc15 [172]. It was hypothesised that the GTP-bound form of Tem1 is additionally required for Cdc15 activity [172]. Tem1 activity is antagonised by its GAP Bub2, which binds via Bfa1 to Tem1. It is hypothesised that Bfa1 must furthermore be phosphorylated by Kin4 to inhibit Tem1 activity [13]. Phosphorylation of Kin4 at T209 by Elm1 is crucial for Kin4 activity [19].

The molecular mechanism which regulates Elm1 activity remains unclear [19]. However, Elm1 phosphorylation of Kin4 depends on SPOC [19], and is inhibited by proper spindle positioning. In response to the correctly aligned spindle, phosphorylation of Kin4 is reversed through PP2A [20], leading to dephosphorylation of the Kin4 site(s) in Bfa1. Lack of Kin4-phosphorylation in Bfa1 allows Cdc5 to phosphorylate Bfa1 [172]. Bfa1 phosphorylated by Cdc5 forms a dimer with Tem1, enabling Tem1 GTPase activity [130, 92]. Active Tem1 can now stimulate Cdc15 kinase activity. Cdc15 is active in a complex consisting of Tem1, Nud1 and Cdc15.

Active Cdc15 phosphorylates Nud1 [172, 132], enabling Nud1 to bind Mob1 [132]. This dimer forms a complex with Dbf2. Dbf2 as part of this complex is phosphorylated by active Cdc15 [92, 172], enabling Dbf2 kinase activity. Dbf2 can now phosphorylate Cdc14 and contribute to Cdc14 activation (Supplementary Figure 14). Cdc14 activation initiates a wave of dephosphorylation of CDK targets, thus, resetting the cell to a state in which it can commence a new cell cycle.

One of these Cdc14 targets is the chitinase Chs2, which, upon Cdc28-Clb1/2-mediated phosphorylation is retained and kept inactive at the endoplasmic reticulum (ER) [159]. Chs2 dephosphorylation by Cdc14 releases Chs2, which is a prerequisite for Chs2 activation. ER release primes Chs2 for Dbf2 phosphorylation, resulting in the activation of Chs2. This node represents the active chitinase complex, which is required to separate the mother from the daughter cell.

Implementation

The cytokinesis module (Supplementary Figure 27) accounts for the integration of signals leading to cell division. First, cytokinesis (node *[Cytokinesis]* requires ongoing exocytosis. Second, the activation of Rho1 involved in symmetry breaking at the mother cell (node *[SymmetryBreakingCyt]*, is implemented as a requirement. Third, Chs2 must be released from the ER in order to activate Dbf2. Dbf2 activation is implemented as a signalling cascade involving Cdc5

phosphorylation of Bfa1, which activates Tem1. Active Tem1 forms a dimer with Cdc15, thus activating it (node *Cdc15Active*). Active Cdc15 phosphorylates Nud1, thus, priming Nud1 to form a a complex with Mob1, which in turn is bound to Dbf2 (node *Dbf2Mob1Nud1*). Active Cdc15 can now phosphorylate and activate Dbf2 (node *Dbf2Active*). Active Dbf2 phosphorylates and releases Chs2 from the ER, thus, removing the inhibitory node *Chs2ERRetention*. Symmetry breaking in the mother cell, exocytosis and active Chs2 are implemented as the three requirements to induce the *Cytokinesis* reaction *Cell_CYT_Cell_[(bud)]*, thereby resetting the status of the bud module to its neutral state.

Interfaces

The cytokinesis module responds to spindle positioning and Cdc5, Cdc28–Cln1/2/3, Cdc28–Clb1/2 and Cdc14 activity. In addition, cytokinesis requires exocytosis.

The output of the module is cytokinesis and phosphorylation and activation of Cdc14.

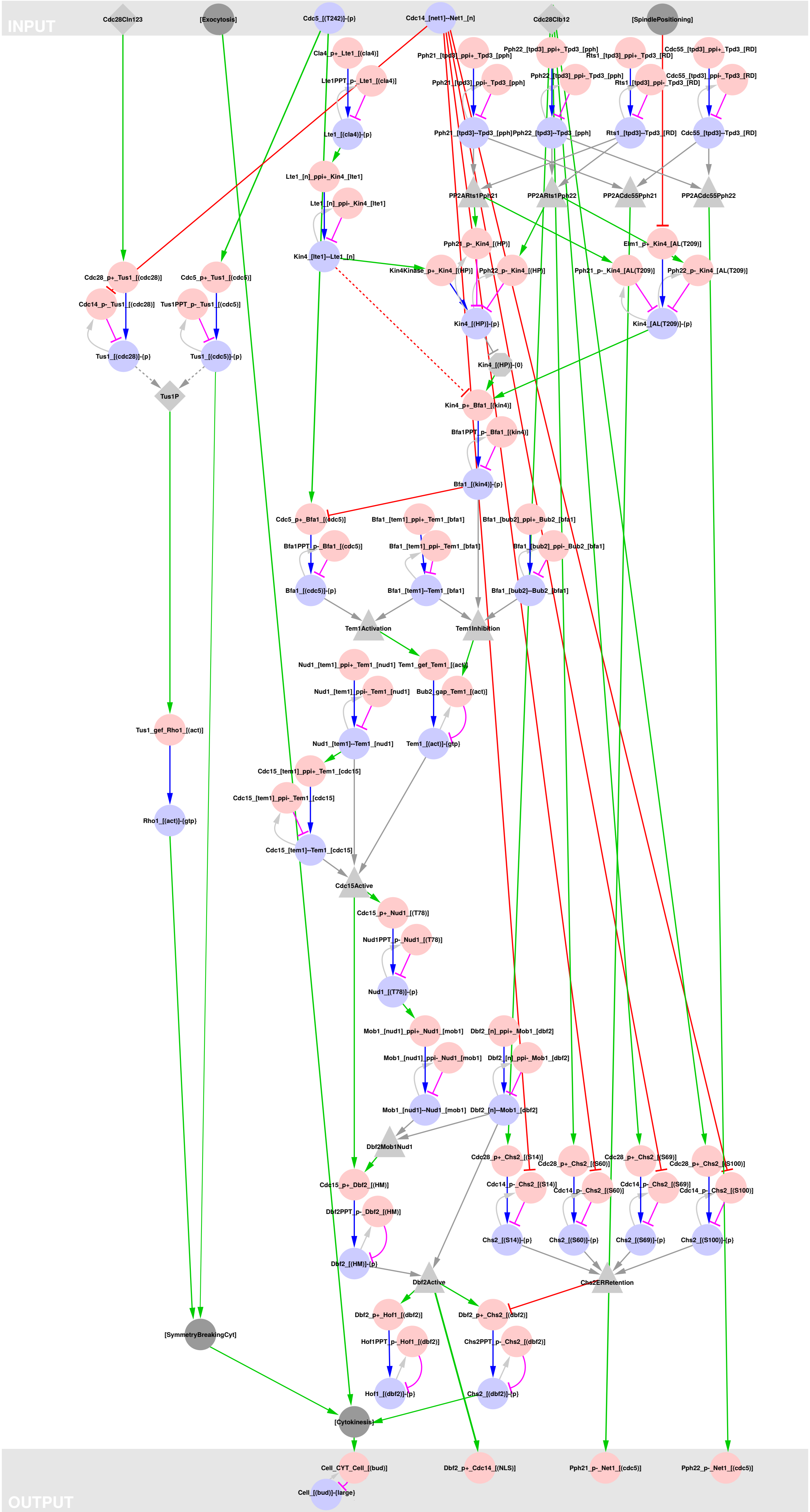

Supplementary Figure 27 *Cytokinesis module.* Correct spindle positioning inactivates the spindle position checkpoint (SPOC), and hence allows the activation of the mitotic exit network (MEN) and hence the Dbf2 kinase. Active Dbf2 reinforces the activation of Cdc14 and triggers release of Chs2, pushing the cell into cytokinesis.

4.8 Additional modules

The CDC model includes four additional modules. These modules have no reported regulation or their output is not connected to the rest of the network. However, the information was included and can serve as a seed for network extensions when more empirical findings become available. Section 4.8.1 contains the subunits of the condensin complex which forms at the centromeres. Section 4.8.2 contains CDK substrates for which no clear role in CDC could be found, yet. Section 4.8.3 contains reactions, which could not be embedded into the overall CDC progression network. The fourth and last module (Section 4.8.4) describes the part of the network which was used to identify certain types of errors in the network, which was helpful in the gap-filling and validation process of the network.

4.8.1 The condensin module

Biology

Condensin is a multimeric protein complex with a not yet well understood role in chromosome segregation [94]. However, condensin which has lost its integrity causes chromosome instability [94].

Implementation

The condensin module (Supplementary Figure 28) accounts for the assembly of the condensin complex at the centromeres. The module accounts for the subunits Brn1, Ycg1, Smc4, Smc2 and Ycs4, combined in the node *Condensin*. The module accounts for condensin binding to the centromere regions via Sgo1, which first binds to the centromeres, thereby recruiting Rts1, and the condensin subunit Smc2. Condensin recruited to the centromeres is then combined in the node *CondensinAtCEN*, which is one prerequisite for tension initiation (node *[TensionInitiation]*, used in the spindle tension module (Supplementary Figure 23).

Interfaces

The condensin complex bound to the centromeres is required for tension initiation (Supplementary Figure 23).

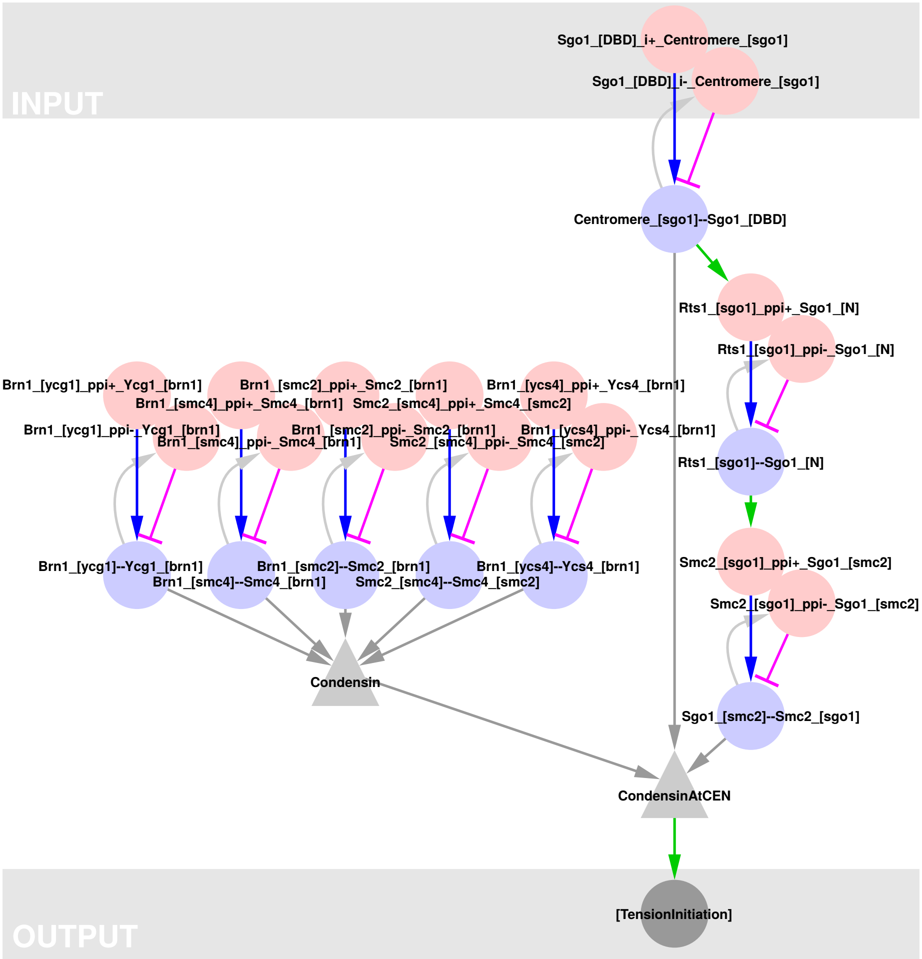

Supplementary Figure 28 *Condensin module.* The module accounts for the condensin subunits Brn1, Ycg1, Smc4, Smc2 and Ycs2. The condensin complex then binds to the centromeres via its subunit Smc2, which in turn binds to centromere bound Sgo1.

4.8.2 Unconnected CDK targets module

Biology

Several CDK Cdc28 and Pho85 substrates have been reported for which the role of CDK-mediated phosphorylation at these residues remains unclear. These substrates and phosphorylations include Cdc24 [15], Ssa1 [163], Smc4 [131], Dbf4 [36, 15], Tgl4 [36], Fin1 [36, 89], Lte1 [5] and Cdc5 [100].

Implementation

The unconnected CDK targets module accounts for the phosphorylation of the above mentioned substrates. For each substrate, the corresponding active CDK state is required.

Interfaces

The outputs of this module are the phosphorylated CDK substrates. In accordance with the dephosphorylation assumption (Section 4.1.2), the CDK substrates are dephosphorylated by Cdc14 upon Cdc14 activation.

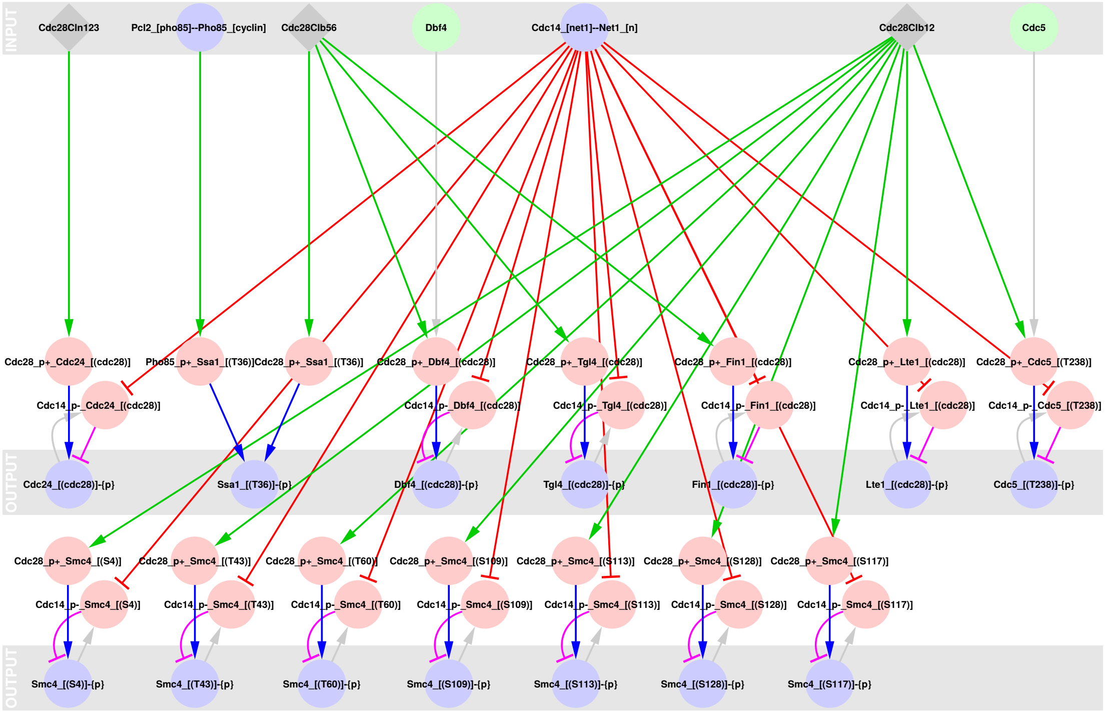

Supplementary Figure 29 *Unconnected CDK targets module.* The model accounts for Cdc28 substrates with no reported function. These substrates may later be functionally connected to the network.

4.8.3 Unconnected states module

Biology

There are several interactions and modification reactions reported in the literature which involves components thought to be involved in the CDC, but for for which neither regulatory constraints nor any effect of the output is known. Such reactions were also collected and added to the CDC model. In total, twelve protein-protein interactions were added: The dimerisation between Cbk1 and Mob2 [172], Cdc12 and Cdc5 [152], Cdc20 and Mad3 [187], Kic1 and Tao3 [172], Hym1 and Kic1 [172], Cdc11 and Cdc5 [152], Cbk1 and Tao3 [172], Hym1 and Sog2 [107], Axl1 and Bud3 [70], Bud3 and Bud4 [70], Cdc42 and Rdi1 [129], and Bud3 and Bud5 [70]. Two phosphorylation reactions were added: Ace2 phosphorylation by Cbk1, which requires Cbk1 to be bound to Mob2 [172], and Pds1 phosphorylation by Chk1 [171].

Implementation

The unconnected states module (Supplementary Figure 30) accounts for the reactions mentioned above which are thought to have a role in CDC progression, but whose mechanistic function remains to be explored. For the two phosphorylation reactions, two hypothesised dephosphorylation were added.

Interfaces

This module requires the existence of the proteins involved and generates the elemental states evolving from these reactions.

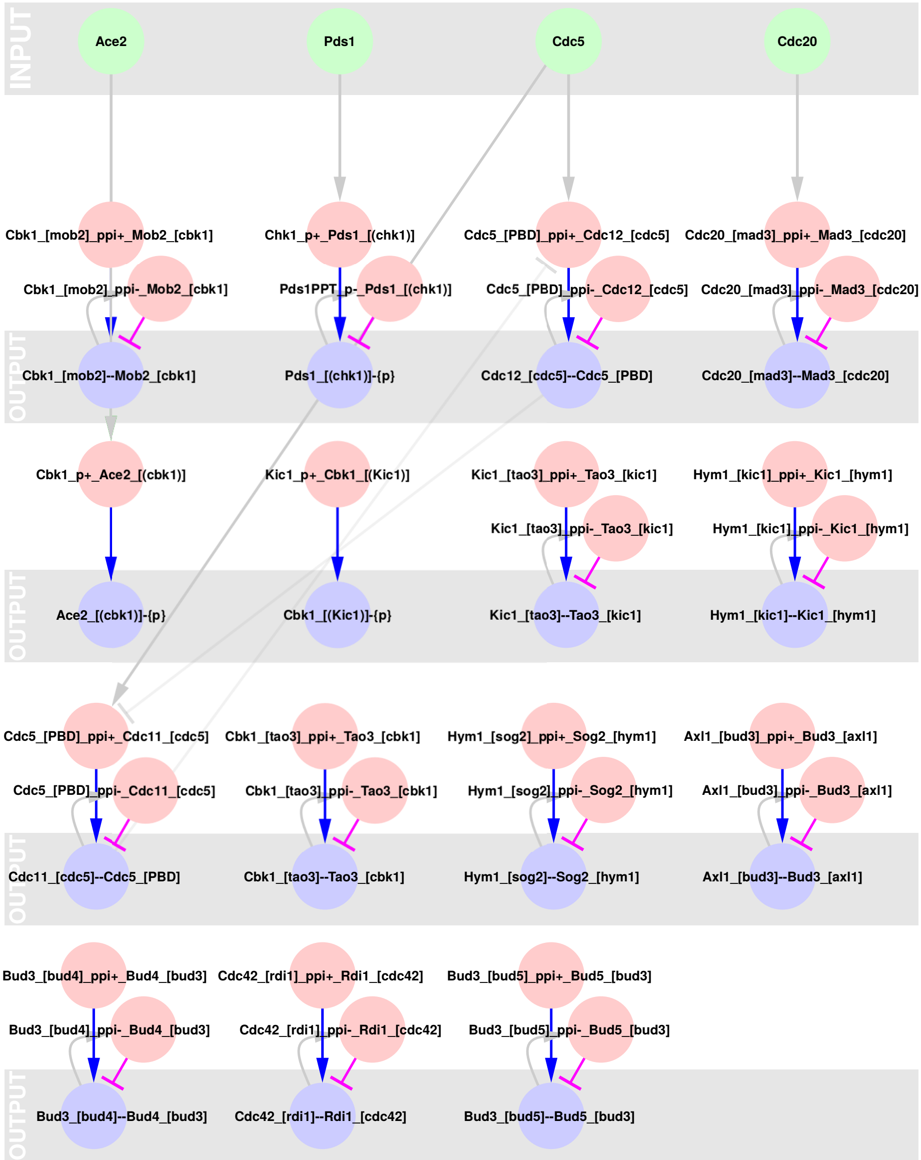

Supplementary Figure 30 *Unconnected states module.* This module takes several interaction and phosphorylation events into account, which concern components involved in CDC control, but for which no mechanistic function has been established.

4.8.4 Test module

Implementation

The test module (Supplementary Figure 31) accounts for the monitoring of several erroneous events during the CDC. These were implemented to monitor the overall state of the CDC during the gap-filling and validation steps. The model accounts for five types of error events. First, the loss of SPB error *[SPBloss]* monitors premature SPB separation before membrane insertion, leading to loss of the newly formed SPB. This refers to a state of the cell where the SPB destined for the daughter cell has started to form but disconnects from the mother SPB before maturing and hence, no tension can be established. Second, the segregation error *[SEGerror]* monitors if the DNA has been separated prior to cytokinesis, leading to chromosome instability. Third, the nuclear division error*[NDerror]* monitors if nuclear division has triggered without chromosome segregation, leading to loss of chromosomes in one nucleus and an increased ploidy of the other. Fourth, the cell division error *[CDerror]* monitors if cytokinesis has occurred before nuclear division or correct spindle alignment, leading to a binuclear cell and a nucleus free cell. Fifth and finally, the *[CriticalError]* monitors if any of these four errors occurs. All five error types have a positive feedback loop on themselves so that once triggered, the error will stay true.

Interfaces

The test module uses outputs from several modules and integrates them to monitor four different errors, and one combination these errors. The module has no effect on the rest of the model and is implemented purely as an analysis tool.

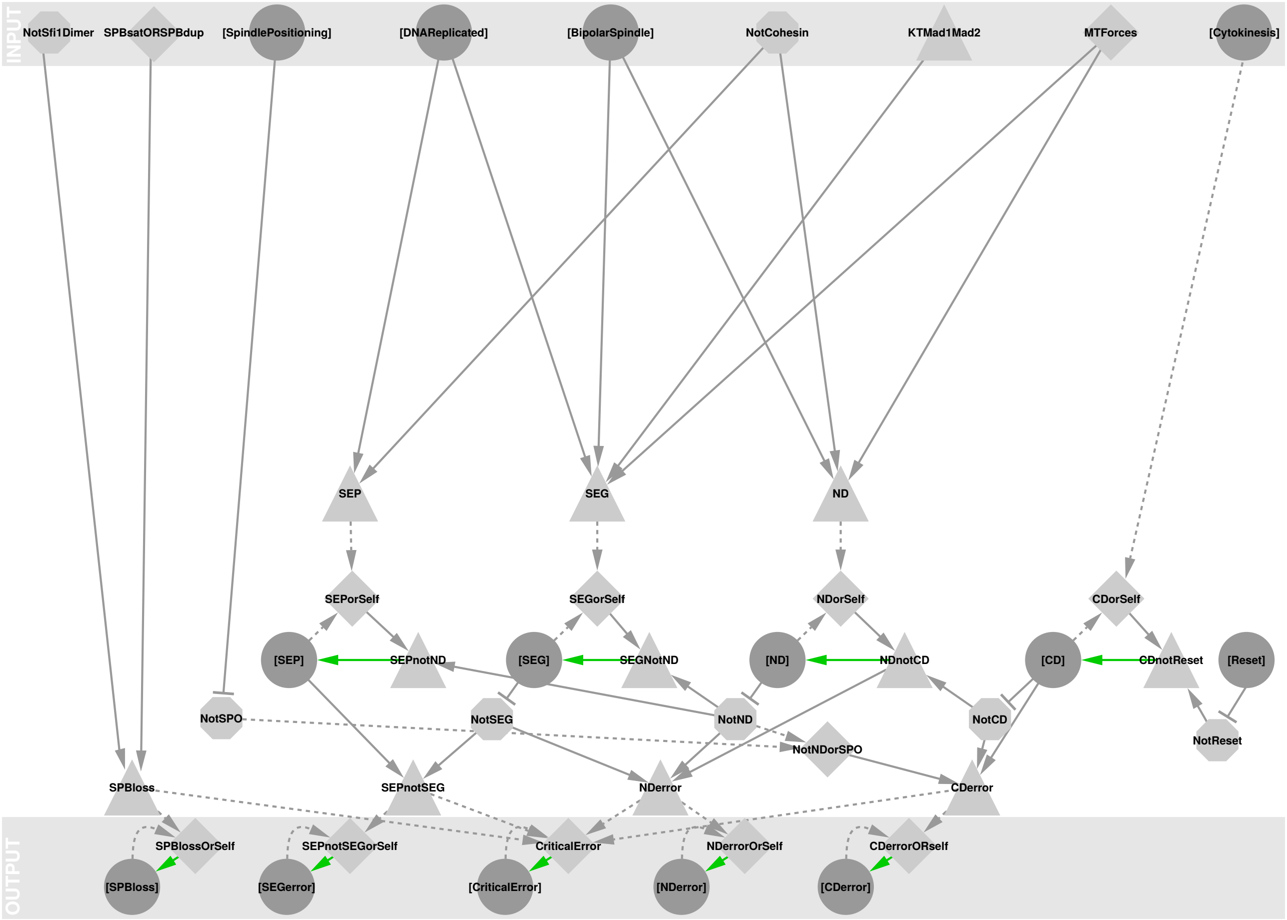

Supplementary Figure 31 *Test module.* The test module monitors and integrates CDC progress and reports the following errors: Loss of SPB (*[SPBloss]*), segregation error (*[SEGerror]*), nuclear division error (*[NDerror]*), cytokinesis error (*[CDerror]*), and finally, an error which monitors if any combination of these four error events occurs (*[CriticalError]*).

Supplementary References

[1] M. Abe, H. Qadota, A. Hirata, and Y. Ohya. Lack of gtp-bound rho1p in secretory vesicles of *Saccharomyces cerevisiae*. *The Journal of Cell Biology*, 162(1):85–97, 2003.

[2] R. Agarwal and O. Cohen-Fix. Phosphorylation of the mitotic regulator pds1/securin by cdc28 is required for efficient nuclear localization of esp1/separase. *Genes & Development*, 16(11):1371–1382, 2002.

[3] A. S. Alberts. Identification of a carboxyl-terminal diaphanous-related formin homology protein autoregulatory domain. *Journal of Biological Chemistry*, 276(4):2824–2830, 2001.

[4] G. Alexandru, F. Uhlmann, K. Mechtler, M.-A. Poupart, and K. Nasmyth. Phosphorylation of the cohesin subunit scc1 by polo/cdc5 kinase regulates sister chromatid separation in yeast. *Cell*, 105(4):459–472, 2001.

[5] V. Archambault, E. J. Chang, B. J. Drapkin, F. R. Cross, B. T. Chait, and M. P. Rout. Targeted proteomic study of the cyclin-cdk module. *Molecular Cell*, 14(6):699–711, 2004.

[6] S. Asano, J.-E. Park, K. Sakchaisri, L.-R. Yu, S. Song, P. Supavilai, T. D. Veenstra, and K. S. Lee. Concerted mechanism of swe1/wee1 regulation by multiple kinases in budding yeast. *The EMBO Journal*, 24(12):2194–2204, 2005.

[7] A. Audhya and S. D. Emr. Stt4 pi 4-kinase localizes to the plasma membrane and functions in the pkc1-mediated map kinase cascade. *Developmental Cell*, 2(5):593–605, 2002.

[8] R. Azzam, S. L. Chen, W. Shou, A. S. Mah, G. Alexandru, K. Nasmyth, R. S. Annan, S. A. Carr, and R. J. Deshaies. Phosphorylation by cyclin b-cdk underlies release of mitotic exit activator cdc14 from the nucleolus. *Science*, 305(5683):516–519, 2004.

[9] N. C. Barbet, U. Schneider, S. B. Helliwell, I. Stansfield, M. F. Tuite, and M. N. Hall. Tor controls translation initiation and early g1 progression in yeast. *Molecular Biology of the Cell*, 7(1):25–42, 1996.

[10] N. Bastajian, H. Friesen, and B. J. Andrews. Bck2 acts through the mads box protein mcm1 to activate cell-cycle-regulated genes in budding yeast. *PLoS Genetics*, 9(5):e1003507, 2013.

[11] J. M. Bean, E. D. Siggia, and F. R. Cross. High functional overlap between mlui cell-cycle box binding factor and swi4/6 cell-cycle box binding factor in the g1/s transcriptional program in *Saccharomyces cerevisiae*. *Genetics*, 171(1):49–61, 2005.

[12] S. P. Bell and K. Labib. Chromosome duplication in *Saccharomyces cerevisiae*. *Genetics*, 203(3):1027–1067, 2016.

[13] D. T. Bertazzi, B. Kurtulmus, and G. Pereira. The cortical protein lte1 promotes mitotic exit by inhibiting the spindle position checkpoint kinase kin4. *The Journal of Cell Biology*, 193(6):1033–1048, 2011.

[14] A. Bertin, M. A. McMurray, P. Grob, S.-S. Park, G. Garcia, I. Patanwala, H.-l. Ng, T. Alber, J. Thorner, and E. Nogales. *Saccharomyces cerevisiae* septins: supramolecular organization of heterooligomers and the mechanism of filament assembly. *Proceedings of the National Academy of Sciences*, 105(24):8274–8279, 2008.

[15] E. Bi and H.-O. Park. Cell polarization and cytokinesis in budding yeast. *Genetics*, 191(2):347–387, 2012.

[16] S. Biggins. The composition, functions, and regulation of the budding yeast kinetochore. *Genetics*, 194(4):817–846, 2013.

[17] E. Bullitt, M. P. Rout, J. V. Kilmartin, and C. W. Akey. The yeast spindle pole body is assembled around a central crystal of spc42p. *Cell*, 89(7):1077–1086, 1997.

[18] A.-C. Butty, N. Perrinjaquet, A. Petit, M. Jaquenoud, J. E. Segall, K. Hofmann, C. Zwahlen, and M. Peter. A positive feedback loop stabilizes the guanine-nucleotide exchange factor cdc24 at sites of polarization. *The EMBO Journal*, 21(7):1565–1576, 2002.

[19] A. K. Caydasi, B. Kurtulmus, M. I. Orrico, A. Hofmann, B. Ibrahim, and G. Pereira. Elm1 kinase activates the spindle position checkpoint kinase kin4. *The Journal of Cell Biology*, 190(6):975–989, 2010.

[20] L. Y. Chan and A. Amon. The protein phosphatase 2a functions in the spindle position checkpoint by regulating the checkpoint kinase kin4. *Genes & Development*, 23(14):1639–1649, 2009.

[21] I. M. Cheeseman, S. Anderson, M. Jwa, E. M. Green, J.-s. Kang, J. R. Yates, C. S. Chan, D. G. Drubin, and G. Barnes. Phospho-regulation of kinetochore-microtubule attachments by the aurora kinase ipl1p. *Cell*, 111(2):163–172, 2002.

[22] S.-h. Chen, M. B. Smolka, and H. Zhou. Mechanism of dun1 activation by rad53 phosphorylation in *Saccharomyces cerevisiae*. *Journal of Biological Chemistry*, 282(2):986–995, 2007.

[23] S.-h. Chen and H. Zhou. Reconstitution of rad53 activation by mec1 through adaptor protein mrc1. *Journal of Biological Chemistry*, 284(28):18593–18604, 2009.

[24] O. Chilkova, P. Stenlund, I. Isoz, C. M. Stith, P. Grabowski, E.-B. Lundström, P. M. Burgers, and E. Johansson. The eukaryotic leading and lagging strand dna polymerases are loaded onto primer-ends via separate mechanisms but have comparable processivity in the presence of pcna. *Nucleic Acids Research*, 35(19):6588–6597, 2007.

[25] R. J. Cho, M. J. Campbell, E. A. Winzeler, L. Steinmetz, A. Conway, L. Wodicka, T. G. Wolfsberg, A. E. Gabrielian, D. Landsman, D. J. Lockhart, et al. A genome-wide transcriptional analysis of the mitotic cell cycle. *Molecular Cell*, 2(1):65–73, 1998.

[26] R. Ciosk, W. Zachariae, C. Michaelis, A. Shevchenko, M. Mann, and K. Nasmyth. An esp1/pds1 complex regulates loss of sister chromatid cohesion at the metaphase to anaphase transition in yeast. *Cell*, 93(6):1067–1076, 1998.

[27] A. Colman-Lerner, T. E. Chin, and R. Brent. Yeast cbk1 and mob2 activate daughter-specific genetic programs to induce asymmetric cell fates. *Cell*, 107(6):739–750, 2001.

[28] M. Costanzo, O. Schub, and B. Andrews. G1 transcription factors are differentially regulated in *Saccharomyces cerevisiae* by the swi6-binding protein stb1. *Molecular and Cellular Biology*, 23(14):5064–5077, 2003.

[29] Z. Darieva, R. Bulmer, A. Pic-Taylor, K. S. Doris, M. Geymonat, S. G. Sedgwick, B. A. Morgan, and A. D. Sharrocks. Polo kinase controls cell cycle-dependent transcription by direct targeting of a coactivator protein. *Nature*, 444(7118):494, 2006.

[30] R. A. de Bruin, T. I. Kalashnikova, C. Chahwan, W. H. McDonald, J. Wohlschlegel, J. Yates, P. Russell, and C. Wittenberg. Constraining g1-specific transcription to late g1 phase: the mbf-associated corepressor nrm1 acts via negative feedback. *Molecular cell*, 23(4):483–496, 2006.

[31] R. A. de Bruin, T. I. Kalashnikova, and C. Wittenberg. Stb1 collaborates with other regulators to modulate the g1-specific transcriptional circuit. *Molecular and Cellular Biology*, 28(22):6919–6928, 2008.

[32] J. M. Dial, E. V. Petrotchenko, and C. H. Borchers. Inhibition of apccdh1 activity by cdh1/acm1/bmh1 ternary complex formation. *Journal of Biological Chemistry*, 282(8):5237–5248, 2007.

[33] Y. Dong, D. Pruyne, and A. Bretscher. Formin-dependent actin assembly is regulated by distinct modes of rho signaling in yeast. *The Journal of cell biology*, 161(6):1081–1092, 2003.

[34] M. Elserafy, M. Šarić, A. Neuner, T.-c. Lin, W. Zhang, C. Seybold, L. Sivashanmugam, and E. Schiebel. Molecular mechanisms that restrict yeast centrosome duplication to one event per cell cycle. *Current Biology*, 24(13):1456–1466, 2014.

[35] M. Enquist-Newman, M. Sullivan, and D. O. Morgan. Modulation of the mitotic regulatory network by apc-dependent destruction of the cdh1 inhibitor acm1. *Molecular Cell*, 30(4):437–446, 2008.

[36] J. M. Enserink and R. D. Kolodner. An overview of Cdk1-controlled targets and processes. *Cell Division*, 5:11, 2010.

[37] S. Erlemann, A. Neuner, L. Gombos, R. Gibeaux, C. Antony, and E. Schiebel. An extended γ-tubulin ring functions as a stable platform in microtubule nucleation. *The Journal of Cell Biology*, 197(1):59–74, 2012.

[38] M. Evangelista, K. Blundell, M. S. Longtine, C. J. Chow, N. Adames, J. R. Pringle, M. Peter, and C. Boone. Bni1p, a yeast formin linking cdc42p and the actin cytoskeleton during polarized morphogenesis. *Science*, 276(5309):118–122, 1997.

[39] M. Evangelista, D. Pruyne, D. C. Amberg, C. Boone, and A. Bretscher. Formins direct arp2/3-independent actin filament assembly to polarize cell growth in yeast. *Nature Cell Biology*, 4(1):32, 2002.

[40] C. Evrin, A. Fernández-Cid, A. Riera, J. Zech, P. Clarke, M. C. Herrera, S. Tognetti, R. Lurz, and C. Speck. The orc/cdc6/mcm2-7 complex facilitates mcm2-7 dimerization during prereplicative complex formation. *Nucleic Acids Research*, 42(4):2257–2269, 2013.

[41] D. Finley, H. D. Ulrich, T. Sommer, and P. Kaiser. The ubiquitin–proteasome system of *Saccharomyces cerevisiae*. *Genetics*, 192(2):319–360, 2012.

[42] M. Foti, A. Audhya, and S. D. Emr. Sac1 lipid phosphatase and stt4 phosphatidylinositol 4-kinase regulate a pool of phosphatidylinositol 4-phosphate that functions in the control of the actin cytoskeleton and vacuole morphology. *Molecular Biology of the Cell*, 12(8):2396–2411, 2001.

[43] L. Francisco, W. Wang, and C. Chan. Type 1 protein phosphatase acts in opposition to ipl1 protein kinase in regulating yeast chromosome segregation. *Molecular and Cellular Biology*, 14(7):4731–4740, 1994.

[44] V. Fridman, A. Gerson-Gurwitz, O. Shapira, N. Movshovich, S. Lakämper, C. F. Schmidt, and L. Gheber. Kinesin-5 kip1 is a bi-directional motor that stabilizes microtubules and tracks their plus-ends in vivo. *J Cell Sci*, 126(18):4147–4159, 2013.

[45] J. Fu, I. M. Hagan, and D. M. Glover. The centrosome and its duplication cycle. *Cold Spring Harbor Perspectives in Biology*, 7(2):a015800, 2015.

[46] L. J. García-Rodríguez, G. De Piccoli, V. Marchesi, R. C. Jones, R. D. Edmondson, and K. Labib. A conserved polϵ binding module in ctf18-rfc is required for s-phase checkpoint activation downstream of mec1. *Nucleic Acids Research*, 43(18):8830–8838, 2015.

[47] M. Geymonat, A. Spanos, G. de Bettignies, and S. G. Sedgwick. Lte1 contributes to bfa1 localization rather than stimulating nucleotide exchange by tem1. *The Journal of Cell Biology*, 187(4):497–511, 2009.

[48] P.-Y. Goh and U. Surana. Cdc4, a protein required for the onset of s phase, serves an essential function during g(2)/m transition in *Saccharomyces cerevisiae*. *Molecular and Cellular Biology*, 19(8):5512–5522, 1999.

[49] W. Guo, F. Tamanoi, and P. Novick. Spatial regulation of the exocyst complex by rho1 gtpase. *Nature Cell Biology*, 3(4):353–360, 2001.

[50] S. B. Haase and C. Wittenberg. Topology and control of the cell-cycle-regulated transcriptional circuitry. *Genetics*, 196(1):65–90, 2014.

[51] L. H. Hartwell, R. K. Mortimer, J. Culotti, and M. Culotti. Genetic control of the cell division cycle in yeast: V. genetic analysis of cdc mutants. *Genetics*, 74(2):267–286, 1973.

[52] B. He, F. Xi, X. Zhang, J. Zhang, and W. Guo. Exo70 interacts with phospholipids and mediates the targeting of the exocyst to the plasma membrane. *The EMBO Journal*, 26(18):4053–4065, 2007.

[53] A. M. Hegnauer, N. Hustedt, K. Shimada, B. L. Pike, M. Vogel, P. Amsler, S. M. Rubin, F. Van Leeuwen, A. Guénolé, H. Van Attikum, et al. An n-terminal acidic region of sgs1 interacts with rpa70 and recruits rad53 kinase to stalled forks. *The EMBO Journal*, 31(18):3768–3783, 2012.

[54] S. Hernández-Ortega, S. Bru, N. Ricco, S. Ramírez, N. Casals, J. Jiménez, M. Isasa, B. Crosas, and J. Clotet. Defective in mitotic arrest 1 (dma1) ubiquitin ligase controls g1 cyclin degradation. *Journal of Biological Chemistry*, 288(7):4704–4714, 2013.

[55] G. P. H. Heusden, D. J. Griffiths, J. C. Ford, P. A. Schrader, A. M. Carr, H. Y. Steensma, et al. The 14-3-3 proteins encoded by the bmh1 and bmh2 genes are essential in the yeast *Saccharomyces cerevisiae* and can be replaced by a plant homologue. *The FEBS Journal*, 229(1):45–53, 1995.

[56] E. R. Hildebrandt, L. Gheber, T. Kingsbury, and M. A. Hoyt. Homotetrameric form of cin8p, a *Saccharomyces cerevisiae* kinesin-5 motor, is essential for its in vivo function. *Journal of Biological Chemistry*, 281(36):26004–26013, 2006.

[57] L. J. Holt, A. N. Krutchinsky, and D. O. Morgan. Positive feedback sharpens the anaphase switch. *Nature*, 454(7202):353, 2008.

[58] C. E. Horak, N. M. Luscombe, J. Qian, P. Bertone, S. Piccirrillo, M. Gerstein, and M. Snyder. Complex transcriptional circuitry at the g1/s transition in *Saccharomyces cerevisiae*. *Genes & Development*, 16(23):3017–3033, 2002.

[59] A. S. Howell and D. J. Lew. Morphogenesis and the cell cycle. *Genetics*, 190(1):51–77, 2012.

[60] S.-C. Hsu, D. TerBush, M. Abraham, and W. Guo. The exocyst complex in polarized exocytosis. volume 233 of *International Review of Cytology*, pages 243 – 265. Academic Press, 2004.

[61] D. Huang, H. Friesen, and B. Andrews. Pho85, a multifunctional cyclin-dependent protein kinase in budding yeast. *Molecular Microbiology*, 66(2):303–314, 2007.

[62] D. Huang, S. Kaluarachchi, D. van Dyk, H. Friesen, R. Sopko, W. Ye, N. Bastajian, J. Moffat, H. Sassi, M. Costanzo, et al. Dual regulation by pairs of cyclin-dependent protein kinases and histone deacetylases controls g1 transcription in budding yeast. *PLoS Biology*, 7(9):e1000188, 2009.

[63] C. Iftode, Y. Daniely, and J. A. Borowiec. Replication protein a (rpa): the eukaryotic ssb. *Critical Reviews in Biochemistry and Molecular Biology*, 34(3):141–180, 1999.

[64] J. Imai, A. Toh-e, and Y. Matsui. Genetic analysis of the saccharomyces cerevisiae rho3 gene, encoding a rho-type small gtpase, provides evidence for a role in bud formation. *Genetics*, 142(2):359–369, 1996.

[65] H. Imamura, K. Tanaka, T. Hihara, M. Umikawa, T. Kamei, K. Takahashi, T. Sasaki, and Y. Takai. Bni1p and bnr1p: downstream targets of the rho family small g-proteins which interact with profilin and regulate actin cytoskeleton in *Saccharomyces cerevisiae*. *The EMBO Journal*, 16(10):2745–2755, 1997.

[66] M. Iwase, J. Luo, S. Nagaraj, M. Longtine, H. B. Kim, B. K. Haarer, C. Caruso, Z. Tong, J. R. Pringle, and E. Bi. Role of a cdc42p effector pathway in recruitment of the yeast septins to the presumptive bud site. *Molecular Biology of the Cell*, 17(3):1110–1125, 2006.

[67] S. L. Jaspersen, B. J. Huneycutt, T. H. Giddings, K. A. Resing, N. G. Ahn, and M. Winey. Cdc28/cdk1 regulates spindle pole body duplication through phosphorylation of spc42 and mps1. *Developmental Cell*, 7(2):263–274, 2004.

[68] D. I. Johnson and J. R. Pringle. Molecular characterization of cdc42, a *Saccharomyces cerevisiae* gene involved in the development of cell polarity. *The Journal of Cell Biology*, 111(1):143–152, 1990.

[69] P. Kaiser, R. A. Sia, E. G. Bardes, D. J. Lew, and S. I. Reed. Cdc34 and the f-box protein met30 are required for degradation of the cdk-inhibitory kinase swe1. *Genes & Development*, 12(16):2587–2597, 1998.

[70] P. J. Kang, M. E. Lee, and H.-O. Park. Bud3 activates cdc42 to establish a proper growth site in budding yeast. *The Journal of Cell Biology*, 206(1):19–28, 2014.

[71] M. A. Keaton, E. S. Bardes, A. R. Marquitz, C. D. Freel, T. R. Zyla, J. Rudolph, and D. J. Lew. Differential susceptibility of yeast s and m phase cdk complexes to inhibitory tyrosine phosphorylation. *Current Biology*, 17(14):1181–1189, 2007.

[72] J. M. Keck, M. H. Jones, C. C. Wong, J. Binkley, D. Chen, S. L. Jaspersen, E. P. Holinger, T. Xu, M. Niepel, M. P. Rout, et al. A cell cycle phosphoproteome of the yeast centrosome. *Science*, 332(6037):1557–1561, 2011.

[73] J. o. Kim, A. Zelter, N. T. Umbreit, A. Bollozos, M. Riffle, R. Johnson, M. J. MacCoss, C. L. Asbury, and T. N. Davis. The ndc80 complex bridges two dam1 complex rings. *eLife*, 6:e21069, 2017.

[74] C. Koch, T. Moll, M. Neuberg, H. Ahorn, and K. Nasmyth. A role for the transcription factors mbp1 and swi4 in progression from g1 to s phase. *Science*, 261(5128):1551–1558, 1993.

[75] C. Koch, A. Schleiffer, G. Ammerer, and K. Nasmyth. Switching transcription on and off during the yeast cell cycle: Cln/cdc28 kinases activate bound transcription factor sbf (swi4/swi6) at start, whereas clb/cdc28 kinases displace it from the promoter in g2. *Genes & Development*, 10(2):129–141, 1996.

[76] H. Kohno, K. Tanaka, A. Mino, M. Umikawa, H. Imamura, T. Fujiwara, Y. Fujita, K. Hotta, H. Qadota, T. Watanabe, et al. Bni1p implicated in cytoskeletal control is a putative target of rho1p small gtp binding protein in *Saccharomyces cerevisiae*. *The EMBO Journal*, 15(22):6060, 1996.

[77] M. Kõivomägi, E. Valk, R. Venta, A. Iofik, M. Lepiku, D. O. Morgan, and M. Loog. Dynamics of cdk1 substrate specificity during the cell cycle. *Molecular Cell*, 42(5):610–623, 2011.

[78] K. Kono, S. Nogami, M. Abe, M. Nishizawa, S. Morishita, D. Pellman, and Y. Ohya. G1/s cyclin-dependent kinase regulates small gtpase rho1p through phosphorylation of rhogef tus1p in *Saccharomyces cerevisiae*. *Molecular Biology of the Cell*, 19(4):1763–1771, 2008.

[79] P. Kraikivski, K. C. Chen, T. Laomettachit, T. Murali, and J. J. Tyson. From start to finish: computational analysis of cell cycle control in budding yeast. *Npj Systems Biology And Applications*, 1:15016, 2015.

[80] C. Kühne and P. Linder. A new pair of b-type cyclins from *Saccharomyces cerevisiae* that function early in the cell cycle. *The EMBO Journal*, 12(9):3437, 1993.

[81] B. D. Landry, J. P. Doyle, D. P. Toczyski, and J. A. Benanti. F-box protein specificity for g1 cyclins is dictated by subcellular localization. *PLoS Genetics*, 8(7):e1002851, 2012.

[82] K. S. Lee, S. Asano, J.-E. Park, K. Sakchaisri, and R. L. Erikson. Monitoring the cell cycle by multi-kinase-dependent regulation of swe1/wee1 in budding yeast. *Cell Cycle*, 4(10):1346–1349, 2005.

[83] T. Legal, J. Zou, A. Sochaj, J. Rappsilber, and J. P. Welburn. Molecular architecture of the dam1 complex–microtubule interaction. *Open Biology*, 6(3):150237, 2016.

[84] M. E. Liku, V. Q. Nguyen, A. W. Rosales, K. Irie, and J. J. Li. Cdk phosphorylation of a novel nls-nes module distributed between two subunits of the mcm2-7 complex prevents chromosomal rereplication. *Molecular Biology of the Cell*, 16(10):5026–5039, 2005.

[85] H. H. Lim, C. J. Loy, S. Zaman, and U. Surana. Dephosphorylation of threonine 169 of cdc28 is not required for exit from mitosis but may be necessary for start in *Saccharomyces cerevisiae*. *Molecular and Cellular Biology*, 16(8):4573–4583, 1996.

[86] T.-c. Lin, A. Neuner, Y. T. Schlosser, E. Schiebel, A. N. Scharf, and L. Weber. Cell-cycle dependent phosphorylation of yeast pericentrin regulates γ-tusc-mediated microtubule nucleation. *Elife*, 3:e02208, 2014.

[87] B. Liu, L. Larsson, A. Caballero, X. Hao, D. Öling, J. Grantham, and T. Nyström. The polarisome is required for segregation and retrograde transport of protein aggregates. *Cell*, 140(2):257–267, 2010.

[88] D. Liu, G. Vader, M. J. Vromans, M. A. Lampson, and S. M. Lens. Sensing chromosome bi-orientation by spatial separation of aurora b kinase from kinetochore substrates. *Science*, 323(5919):1350–1353, 2009.

[89] M. Loog and D. O. Morgan. Cyclin specificity in the phosphorylation of cyclin-dependent kinase substrates. *Nature*, 434(7029):104–108, 2005.

[90] M. Lõoke, M. F. Maloney, and S. P. Bell. Mcm10 regulates dna replication elongation by stimulating the cmg replicative helicase. *Genes & Development*, 31(3):291–305, 2017.

[91] K. D. MacIsaac, T. Wang, D. B. Gordon, D. K. Gifford, G. D. Stormo, and E. Fraenkel. An improved map of conserved regulatory sites for *Saccharomyces cerevisiae*. *BMC Bioinformatics*, 7(1):113, 2006.

[92] A. S. Mah, J. Jang, and R. J. Deshaies. Protein kinase cdc15 activates the dbf2-mob1 kinase complex. *Proceedings of the National Academy of Sciences*, 98(13):7325–7330, 2001.

[93] S. M. Markus, K. A. Kalutkiewicz, and W.-L. Lee. Astral microtubule asymmetry provides directional cues for spindle positioning in budding yeast. *Experimental Cell Research*, 318(12):1400–1406, 2012.

[94] A. L. Marston. Chromosome segregation in budding yeast: sister chromatid cohesion and related mechanisms. *Genetics*, 196(1):31–63, 2014.

[95] V. Measday, L. Moore, R. Retnakaran, J. Lee, M. Donoviel, A. Neiman, and B. Andrews. A family of cyclin-like proteins that interact with the pho85 cyclin-dependent kinase. *Molecular and Cellular Biology*, 17(3):1212–1223, 1997.

[96] M. D. Mendenhall and A. E. Hodge. Regulation of cdc28 cyclin-dependent protein kinase activity during the cell cycle of the yeast *Saccharomyces cerevisiae*. *Microbiology and Molecular Biology Reviews*, 62(4):1191–1243, 1998.

[97] J. L. Miranda, D. S. King, and S. C. Harrison. Protein arms in the kinetochore-microtubule interface of the yeast dash complex. *Molecular Biology of the Cell*, 18(7):2503–2510, 2007.

[98] D. A. Mohl, M. J. Huddleston, T. S. Collingwood, R. S. Annan, and R. J. Deshaies. Dbf2–mob1 drives relocalization of protein phosphatase cdc14 to the cytoplasm during exit from mitosis. *The Journal of Cell Biology*, 184(4):527–539, 2009.

[99] J. K. Moore and R. K. Miller. The cyclin-dependent kinase cdc28p regulates multiple aspects of kar9p function in yeast. *Molecular Biology of the Cell*, 18(4):1187–1202, 2007.

[100] E. M. Mortensen, W. Haas, M. Gygi, S. P. Gygi, and D. R. Kellogg. Cdc28-dependent regulation of the cdc5/polo kinase. *Current Biology*, 15(22):2033–2037, 2005.

[101] J. B. Moseley and B. L. Goode. The yeast actin cytoskeleton: from cellular function to biochemical mechanism. *Microbiology and Molecular Biology Reviews*, 70(3):605–645, 2006.

[102] P. Müller, S. Park, E. Shor, D. J. Huebert, C. L. Warren, A. Z. Ansari, M. Weinreich, M. L. Eaton, D. M. MacAlpine, and C. A. Fox. The conserved bromo-adjacent homology domain of yeast orc1 functions in the selection of dna replication origins within chromatin. *Genes & Development*, 24(13):1418–1433, 2010.

[103] Y. Nakajima, R. G. Tyers, C. C. Wong, J. R. Yates, D. G. Drubin, and G. Barnes. Nbl1p: a borealin/dasra/csc-1-like protein essential for aurora/ipl1 complex function and integrity in *Saccharomyces cerevisiae*. *Molecular Biology of the Cell*, 20(6):1772–1784, 2009.

[104] P. Nash, X. Tang, S. Orlicky, Q. Chen, F. B. Gertler, M. D. Mendenhall, F. Sicheri, T. Pawson, and M. Tyers. Multisite phosphorylation of a cdk inhibitor sets a threshold for the onset of dna replication. *Nature*, 414(6863):514–521, 2001.

[105] K. Nasmyth and L. Dirick. The role of swi4 and swi6 in the activity of g1 cyclins in yeast. *Cell*, 66(5):995–1013, 1991.

[106] E. Nazarova, E. O'Toole, S. Kaitna, P. Francois, M. Winey, and J. Vogel. Distinct roles for antiparallel microtubule pairing and overlap during early spindle assembly. *Molecular Biology of the Cell*, 24(20):3238–3250, 2013.

[107] B. Nelson, C. Kurischko, J. Horecka, M. Mody, P. Nair, L. Pratt, A. Zougman, L. D. McBroom, T. R. Hughes, C. Boone, et al. Ram: a conserved signaling network that regulates ace2p transcriptional activity and polarized morphogenesis. *Molecular Biology of the Cell*, 14(9):3782–3803, 2003.

[108] V. Q. Nguyen, J. J. Li, et al. Cyclin-dependent kinases prevent dna re-replication through multiple mechanisms. *Nature*, 411(6841):1068–1073, 2001.

[109] M. Nishizawa, M. Kawasumi, M. Fujino, and A. Toh-e. Phosphorylation of sic1, a cyclin-dependent kinase (cdk) inhibitor, by cdk including pho85 kinase is required for its prompt degradation. *Molecular Biology of the Cell*, 9(9):2393–2405, 1998.

[110] M. Nishizawa, K. Suzuki, M. Fujino, T. Oguchi, and A. Toh-e. The pho85 kinase, a member of the yeast cyclin-dependent kinase (cdk) family, has a regulation mechanism different from cdks functioning throughout the cell cycle. *Genes to Cells*, 4(11):627–642, 1999.

[111] D. Ostapenko, J. L. Burton, R. Wang, and M. J. Solomon. Pseudosubstrate inhibition of the anaphase-promoting complex by acm1: regulation by proteolysis and cdc28 phosphorylation. *Molecular and Cellular Biology*, 28(15):4653–4664, 2008.

[112] K. Ozaki, K. Tanaka, H. Imamura, T. Hihara, T. Kameyama, H. Nonaka, H. Hirano, Y. Matsuura, and Y. Takai. Rom1p and rom2p are gdp/gtp exchange proteins (geps) for the rho1p small gtp binding protein in *Saccharomyces cerevisiae*. *The EMBO Journal*, 15(9):2196, 1996.

[113] G. Palou, R. Palou, F. Zeng, A. A. Vashisht, J. A. Wohlschlegel, and D. G. Quintana. Three different pathways prevent chromosome segregation in the presence of dna damage or replication stress in budding yeast. *PLoS Genetics*, 11(9):e1005468, 2015.

[114] P. Palumbo, M. Vanoni, V. Cusimano, S. Busti, F. Marano, C. Manes, and L. Alberghina. Whi5 phosphorylation embedded in the g1/s network dynamically controls critical cell size and cell fate. *Nature Communications*, 7, 2016.

[115] B. Pardo, L. Crabbé, and P. Pasero. Signaling pathways of replication stress in yeast. *FEMS Yeast Research*, 17(2), 2017.

[116] A. M. Perez, G. C. Finnigan, F. M. Roelants, and J. Thorner. Septin-associated protein kinases in the yeast *Saccharomyces cerevisiae*. *Frontiers in Cell and Developmental Biology*, 4, 2016.

[117] G. Perkins, L. S. Drury, and J. F. Diffley. Separate scf cdc4 recognition elements target cdc6 for proteolysis in s phase and mitosis. *The EMBO Journal*, 20(17):4836–4845, 2001.

[118] M. Peter and I. Herskowitz. Direct inhibition of the yeast cyclin-dependent kinase cdc28-cln by far1. *Science*, 265(5176):1228–1232, 1994.

[119] B. Philip and D. E. Levin. Wsc1 and mid2 are cell surface sensors for cell wall integrity signaling that act through rom2, a guanine nucleotide exchange factor for rho1. *Molecular and Cellular Biology*, 21(1):271–280, 2001.

[120] A. Pic-Taylor, Z. Darieva, B. A. Morgan, and A. D. Sharrocks. Regulation of cell cycle-specific gene expression through cyclin-dependent kinase-mediated phosphorylation of the forkhead transcription factor fkh2p. *Molecular and Cellular Biology*, 24(22):10036–10046, 2004.

[121] T. Pramila, S. Miles, D. GuhaThakurta, D. Jemiolo, and L. L. Breeden. Conserved homeodomain proteins interact with mads box protein mcm1 to restrict ecb-dependent transcription to the m/g1 phase of the cell cycle. *Genes & Development*, 16(23):3034–3045, 2002.

[122] T. Pramila, W. Wu, S. Miles, W. S. Noble, and L. L. Breeden. The forkhead transcription factor hcm1 regulates chromosome segregation genes and fills the s-phase gap in the transcriptional circuitry of the cell cycle. *Genes & Development*, 20(16):2266–2278, 2006.

[123] D. Pruyne, M. Evangelista, C. Yang, E. Bi, S. Zigmond, A. Bretscher, and C. Boone. Role of formins in actin assembly: nucleation and barbed-end association. *Science*, 297(5581):612–615, 2002.

[124] H. Qadota, C. P. Python, S. B. Inoue, M. Arisawa, Y. Anraku, Y. Zheng, T. Watanabe, D. E. Levin, and Y. Ohya. Identification of yeast rho1p gtpase as a regulatory subunit of 1, 3-β-glucan synthase. *Science*, pages 279–281, 1996.

[125] E. Queralt, C. Lehane, B. Novak, and F. Uhlmann. Downregulation of pp2a cdc55 phosphatase by separase initiates mitotic exit in budding yeast. *Cell*, 125(4):719–732, 2006.

[126] G. J. Reynard, W. Reynolds, R. Verma, and R. J. Deshaies. Cks1 is required for g1cyclin–cyclin-dependent kinase activity in budding yeast. *Molecular and Cellular Biology*, 20(16):5858–5864, 2000.

[127] D. Reynolds, B. J. Shi, C. McLean, F. Katsis, B. Kemp, and S. Dalton. Recruitment of thr 319-phosphorylated ndd1p to the fha domain of fkh2p requires clbkinase activity: a mechanism for clb cluster gene activation. *Genes & Development*, 17(14):1789–1802, 2003.

[128] H. E. Richardson, C. Wittenberg, F. Cross, and S. I. Reed. An essential g1 function for cyclin-like proteins in yeast. *Cell*, 59(6):1127–1133, 1989.

[129] T. J. Richman, K. A. Toenjes, S. E. Morales, K. C. Cole, B. T. Wasserman, C. M. Taylor, J. A. Koster, M. F. Whelihan, and D. I. Johnson. Analysis of cell-cycle specific localization of the rdi1p rhogdi and the structural determinants required for cdc42p membrane localization and clustering at sites of polarized growth. *Current Genetics*, 45(6):339–349, Jun 2004.

[130] H.-S. Ro, S. Song, and K. S. Lee. Bfa1 can regulate tem1 function independently of bub2 in the mitotic exit network of *Saccharomyces cerevisiae*. *Proceedings of the National Academy of Sciences*, 99(8):5436–5441, 2002.

[131] X. Robellet, Y. Thattikota, F. Wang, T.-L. Wee, M. Pascariu, S. Shankar, É. Bonneil, C. M. Brown, and D. D'Amours. A high-sensitivity phospho-switch triggered by cdk1 governs chromosome morphogenesis during cell division. *Genes & Development*, 29(4):426–439, 2015.

[132] J. M. Rock, D. Lim, L. Stach, R. W. Ogrodowicz, J. M. Keck, M. H. Jones, C. C. Wong, J. R. Yates, M. Winey, S. J. Smerdon, et al. Activation of the yeast hippo pathway by phosphorylation-dependent assembly of signaling complexes. *Science*, 340(6134):871–875, 2013.

[133] J.-A. Rodriguez-Rodriguez, Y. Moyano, S. Játiva, and E. Queralt. Mitotic exit function of polo-like kinase cdc5 is dependent on sequential activation by cdk1. *Cell Reports*, 15(9):2050–2062, 2016.

[134] J. Romers, S. Thieme, U. Münzner, and M. Krantz. A scalable method for parameter-free simulation and validation of mechanistic cellular signal transduction network models. *bioRxiv*, 2017.

[135] J. Roostalu, C. Hentrich, P. Bieling, I. A. Telley, E. Schiebel, and T. Surrey. Directional switching of the kinesin cin8 through motor coupling. *Science*, 332(6025):94–99, 2011.

[136] J. S. Rosenberg, F. R. Cross, and H. Funabiki. Knl1/spc105 recruits pp1 to silence the spindle assembly checkpoint. *Current Biology*, 21(11):942–947, 2011.

[137] K. E. Ross, P. Kaldis, and M. J. Solomon. Activating phosphorylation of the *Saccharomyces cerevisiae* cyclin-dependent kinase, cdc28p, precedes cyclin binding. *Molecular Biology of the Cell*, 11(5):1597–1609, 2000.

[138] O. Roumanie, H. Wu, J. N. Molk, G. Rossi, K. Bloom, and P. Brennwald. Rho gtpase regulation of exocytosis in yeast is independent of gtp hydrolysis and polarization of the exocyst complex. *The Journal of Cell Biology*, 170(4):583–594, 2005.

[139] J. Rouse and S. P. Jackson. Lcd1p recruits mec1p to dna lesions in vitro and in vivo. *Molecular Cell*, 9(4):857–869, 2002.

[140] D. Rüthnick and E. Schiebel. Duplication of the yeast spindle pole body once per cell cycle. *Molecular and Cellular Biology*, 36(9):1324–1331, 2016.

[141] M. Sbia, E. J. Parnell, Y. Yu, A. E. Olsen, K. L. Kretschmann, W. P. Voth, and D. J. Stillman. Regulation of the yeast ace2 transcription factor during the cell cycle. *Journal of Biological Chemistry*, 283(17):11135–11145, 2008.

[142] T. Schmelzle, S. B. Helliwell, and M. N. Hall. Yeast protein kinases and the rho1 exchange factor tus1 are novel components of the cell integrity pathway in yeast. *Molecular and Cellular Biology*, 22(5):1329–1339, 2002.

[143] J. M. Scholey, G. Civelekoglu-Scholey, and I. Brust-Mascher. Anaphase b. *Biology*, 5(4):51, 2016.

[144] A. Schreiber, F. Stengel, Z. Zhang, R. I. Enchev, E. H. Kong, E. P. Morris, C. V. Robinson, P. C. da Fonseca, and D. Barford. Structural basis for the subunit assembly of the anaphase-promoting complex. *Nature*, 470(7333):227, 2011.

[145] S. C. Schuyler, J. Y. Liu, and D. Pellman. The molecular function of ase1p. *The Journal of Cell Biology*, 160(4):517–528, 2003.

[146] E. Schwob and K. Nasmyth. Clb5 and clb6, a new pair of b cyclins involved in s phase and mitotic spindle formation in *S. cerevisiae*. *Genes & Development*, 7:1160–1175, 1993.

[147] J. H. Seol, R. R. Feldman, W. Zachariae, A. Shevchenko, C. C. Correll, S. Lyapina, Y. Chi, M. Galova, J. Claypool, S. Sandmeyer, et al. Cdc53/cullin and the essential hrt1 ring–h2 subunit of scf define a ubiquitin ligase module that activates the e2 enzyme cdc34. *Genes & Development*, 13(12):1614–1626, 1999.

[148] W. Shou, J. H. Seol, A. Shevchenko, C. Baskerville, D. Moazed, Z. S. Chen, J. Jang, A. Shevchenko, H. Charbonneau, and R. J. Deshaies. Exit from mitosis is triggered by tem1-dependent release of the protein phosphatase cdc14 from nucleolar rent complex. *Cell*, 97(2):233–244, 1999.

[149] R. Sia, H. A. Herald, and D. J. Lew. Cdc28 tyrosine phosphorylation and the morphogenesis checkpoint in budding yeast. *Molecular Biology of the Cell*, 7(11):1657–1666, 1996.

[150] I. Simon, J. Barnett, N. Hannett, C. T. Harbison, N. J. Rinaldi, T. L. Volkert, J. J. Wyrick, J. Zeitlinger, D. K. Gifford, T. S. Jaakkola, et al. Serial regulation of transcriptional regulators in the yeast cell cycle. *Cell*, 106(6):697–708, 2001.

[151] D. Skowyra, K. L. Craig, M. Tyers, S. J. Elledge, and J. W. Harper. F-box proteins are receptors that recruit phosphorylated substrates to the scf ubiquitin-ligase complex. *Cell*, 91(2):209–219, 1997.

[152] S. Song and K. S. Lee. A novel function of *Saccharomyces cerevisiae* cdc5 in cytokinesis. *The Journal of Cell Biology*, 152(3):451–470, 2001.

[153] P. K. Sorger and A. W. Murray. S-phase feedback control in budding yeast independent of tyrosine phosphorylation of p34cdc28. *Nature*, 355:365–368, 1992.

[154] P. T. Spellman, G. Sherlock, M. Q. Zhang, V. R. Iyer, K. Anders, M. B. Eisen, P. O. Brown, D. Botstein, and B. Futcher. Comprehensive identification of cell cycle–regulated genes of the yeast *Saccharomyces cerevisiae* by microarray hybridization. *Molecular Biology of the Cell*, 9(12):3273–3297, 1998.

[155] L. E. Stolz, W. J. Kuo, J. Longchamps, M. K. Sekhon, and J. D. York. Inp51, a yeast inositol polyphosphate 5-phosphatase required for phosphatidylinositol 4, 5-bisphosphate homeostasis and whose absence confers a cold-resistant phenotype. *Journal of Biological Chemistry*, 273(19):11852–11861, 1998.

[156] U. Surana, H. Robitsch, C. Price, T. Schuster, I. Fitch, A. B. Futcher, and K. Nasmyth. The role of cdc28 and cyclins during mitosis in the budding yeast *S. cerevisiae*. *Cell*, 65(1):145–161, 1991.

[157] Y.-S. Tak, Y. Tanaka, S. Endo, Y. Kamimura, and H. Araki. A cdk-catalysed regulatory phosphorylation for formation of the dna replication complex sld2–dpb11. *The EMBO Journal*, 25(9):1987–1996, 2006.

[158] S. Tanaka, T. Umemori, K. Hirai, S. Muramatsu, Y. Kamimura, and H. Araki. Cdk-dependent phosphorylation of sld2 and sld3 initiates dna replication in budding yeast. *Nature*, 445(7125):328, 2007.

[159] E. M. Teh, C. C. Chai, and F. M. Yeong. Retention of chs2p in the er requires n-terminal cdk1-phosphorylation sites. *Cell Cycle*, 8(18):2965–2976, 2009.

[160] C. N. Tennyson, J. Lee, and B. J. Andrews. A role for the pcl9-pho85 cyclin–cdk complex at the m/g1 boundary in *Saccharomyces cerevisiae*. *Molecular Microbiology*, 28(1):69–79, 1998.

[161] A. Travesa, T. I. Kalashnikova, R. A. de Bruin, S. R. Cass, C. Chahwan, D. E. Lee, N. F. Lowndes, and C. Wittenberg. Repression of g1/s transcription is mediated via interaction of the gtb motifs of nrm1 and whi5 with swi6. *Molecular and Cellular Biology*, 33(8):1476–1486, 2013.

[162] A. Travesa, D. Kuo, R. A. De Bruin, T. I. Kalashnikova, M. Guaderrama, K. Thai, A. Aslanian, M. B. Smolka, J. R. Yates, T. Ideker, et al. Dna replication stress differentially regulates g1/s genes via rad53-dependent inactivation of nrm1. *The EMBO Journal*, 31(7):1811–1822, 2012.

[163] A. W. Truman, K. Kristjansdottir, D. Wolfgeher, N. Hasin, S. Polier, H. Zhang, S. Perrett, C. Prodromou, G. W. Jones, and S. J. Kron. Cdk-dependent hsp70 phosphorylation controls g1 cyclin abundance and cell-cycle progression. *Cell*, 151(6):1308–1318, 2012.

[164] F. Uhlmann, F. Lottspeich, and K. Nasmyth. Sister-chromatid separation at anaphase onset is promoted by cleavage of the cohesin subunit scc1. *Nature*, 400(6739):37, 1999.

[165] X. Varelas, D. Stuart, M. J. Ellison, and C. Ptak. The cdc34/scf ubiquitination complex mediates *Saccharomyces cerevisiae* cell wall integrity. *Genetics*, 174(4):1825–1839, 2006.

[166] M. Versele and J. Thorner. Septin collar formation in budding yeast requires gtp binding and direct phosphorylation by the pak, cla4. *The Journal of Cell Biology*, 164(5):701–715, 2004.

[167] R. Visintin, S. Prinz, and A. Amon. Cdc20 and cdh1: A family of substrate-specific activators of apc-dependent proteolysis. *Science*, 278(5337):460–463, 1997.

[168] M. V. Wagner, M. B. Smolka, R. A. De Bruin, H. Zhou, C. Wittenberg, and S. F. Dowdy. Whi5 regulation by site specific cdk-phosphorylation in *Saccharomyces cerevisiae*. *PLoS One*, 4(1):e4300, 2009.

[169] T. Wakayama, T. Kondo, S. Ando, K. Matsumoto, and K. Sugimoto. Pie1, a protein interacting with mec1, controls cell growth and checkpoint responses in *Saccharomyces cerevisiae*. *Molecular and Cellular Biology*, 21(3):755–764, 2001.

[170] H. Wang, E. Garí, E. Verges, C. Gallego, and M. Aldea. Recruitment of cdc28 by whi3 restricts nuclear accumulation of the g1 cyclin–cdk complex to late g1. *The EMBO Journal*, 23(1):180–190, 2004.

[171] H. Wang, D. Liu, Y. Wang, J. Qin, and S. J. Elledge. Pds1 phosphorylation in response to dna damage is essential for its dna damage checkpoint function. *Genes & Development*, 15(11):1361–1372, 2001.

[172] E. L. Weiss. Mitotic exit and separation of mother and daughter cells. *Genetics*, 192(4):1165–1202, 2012.

[173] A. C. Wild, W. Y. Jong, M. A. Lemmon, and K. J. Blumer. The p21-activated protein kinase-related kinase cla4 is a coincidence detector of signaling by cdc42 and phosphatidylinositol 4-phosphate. *Journal of Biological Chemistry*, 279(17):17101–17110, 2004.

[174] M. Winey and K. Bloom. Mitotic spindle form and function. *Genetics*, 190(4):1197–1224, 2012.

[175] H. Wu, C. Turner, J. Gardner, B. Temple, and P. Brennwald. The exo70 subunit of the exocyst is an effector for both cdc42 and rho3 function in polarized exocytosis. *Molecular biology of the cell*, 21(3):430–442, 2010.

[176] Y. Xu, J. B. Moseley, I. Sagot, F. Poy, D. Pellman, B. L. Goode, and M. J. Eck. Crystal structures of a formin homology-2 domain reveal a tethered dimer architecture. *Cell*, 116(5):711–723, 2004.

[177] J. A. Yaglom, A. L. Goldberg, D. Finley, and M. Y. Sherman. The molecular chaperone ydj1 is required for the p34cdc28-dependent phosphorylation of the cyclin cln3 that signals its degradation. *Molecular and Cellular Biology*, 16(7):3679–3684, 1996.

[178] Y. Yamagishi, T. Sakuno, Y. Goto, and Y. Watanabe. Kinetochore composition and its function: lessons from yeasts. *FEMS Microbiology Reviews*, 38(2):185–200, 2014.

[179] J. T. Yeeles, T. D. Deegan, A. Janska, A. Early, and J. F. Diffley. Regulated eukaryotic dna replication origin firing with purified proteins. *Nature*, 519(7544):431–435, 2015.

[180] S. K. Yelamanchi, J. Veis, D. Anrather, H. Klug, and G. Ammerer. Genotoxic stress prevents ndd1-dependent transcriptional activation of g2/m-specific genes in *Saccharomyces cerevisiae*. *Molecular and Cellular Biology*, 34(4):711–724, 2014.

[181] H. Yin, D. Pruyne, T. C. Huffaker, and A. Bretscher. Myosin v orientates the mitotic spindle in yeast. *Nature*, 406(6799):1013, 2000.

[182] H.-J. Yoon and J. Carbon. Participation of bir1p, a member of the inhibitor of apoptosis family, in yeast chromosome segregation events. *Proceedings of the National Academy of Sciences*, 96(23):13208–13213, 1999.

[183] S. Yoshida, K. Kono, D. M. Lowery, S. Bartolini, M. B. Yaffe, Y. Ohya, and D. Pellman. Polo-like kinase cdc5 controls the local activation of rho1 to promote cytokinesis. *Science*, 313(5783):108–111, 2006.

[184] S. Yoshida, Y. Ohya, M. Goebl, A. Nakano, and Y. Anraku. A novel gene, stt4, encodes a phosphatidylinositol 4-kinase in the pkc1 protein kinase pathway of *Saccharomyces cerevisiae*. *Journal of Biological Chemistry*, 269(2):1166–1172, 1994.

[185] P. Zarzov, C. Mazzoni, and C. Mann. The slt2(mpk1) map kinase is activated during periods of polarized cell growth in yeast. *The EMBO Journal*, 15(1):83–91, 1996.

[186] P. Zheng, D. Fay, J. Burton, H. Xiao, J. Pinkham, and D. Stern. Spk1 is an essential s-phase-specific gene of *Saccharomyces cerevisiae* that encodes a nuclear serine/threonine/tyrosine kinase. *Molecular and Cellular Biology*, 13(9):5829–5842, 1993.

[187] J. Zich and K. G. Hardwick. Getting down to the phosphorylated 'nuts and bolts' of spindle checkpoint signalling. *Trends in Biochemical Sciences*, 35(1):18–27, 2010.
