# Supplementary material for: A comprehensive, mechanistically detailed, and executable model of the cell division cycle in Saccharomyces cerevisiae
Source: Nat Commun. 2019 Mar 21;10:1308. doi: 10.1038/s41467-019-08903-w (PMC6428898; doi:10.1038/s41467-019-08903-w)
Supplement: Supplementary file 2 — Description of Additional Supplementary Files [file 41467_2019_8903_MOESM2_ESM.docx]

**Description of Additional Supplementary Files**

File Name: Supplementary Data 1

Description: The rxncon model. The rxncon network definition consists of four sheets: (i) A reaction list defining all elemental reactions - and implicitly all elemental states - of both the CGM and the MRN, (ii) a contingency list defining the contextual constraints on the elemental reactions, (iii) a reaction definition which gives the semantic meaning of all elemental reactions, and (iv) a modification definition which lists all valid modifiers. The structure and use of the rxncon model file is described in detail elsewhere [22, 44]. The GSM is also available at https://github.com/rxncon/models/ as CDC_S_cerevisiae.xls.

File Name: Supplementary Data 2

Description: Cytoscape file. The cytoscape file containing the regulatory graph of the complete network as well as of the individual modules presented in the supplementary figures.

File Name: Supplementary Data 3-5

Description: Boolean model, initial vector, label key. These three files where used to simulate the network model, as described in detail elsewhere [21]. Supplementary Data 3 contains the model code, formatted for simulation using BoolNet. Supplementary Data 4 contains the initial vector. Supplementary Data 5 defines the mapping between the short names used in 3 and 4 to the names used in the rxncon file.
